# Satellite gravity gradient grids for geophysics

**DOI:** 10.1038/srep21050

**Published:** 2016-02-11

**Authors:** Johannes Bouman, Jörg Ebbing, Martin Fuchs, Josef Sebera, Verena Lieb, Wolfgang Szwillus, Roger Haagmans, Pavel Novak

**Affiliations:** 1 Deutsches Geodätisches Forschungsinstitut der Technischen Universität München (DGFI-TUM), Munich, Germany; 2 CAU Kiel, Kiel, Germany; 3 Astronomical Institute of the Czech Academy of Sciences, Ondrejov, Czech Republic; 4 Research Institute of Geodesy, Cartography and Topography, Zbidy, Czech Republic; 5 ESA-ESTEC, Noordwijk, the Netherlands; 6 UWB, Plzeň, Czech Republic

## Abstract

The Gravity field and steady-state Ocean Circulation Explorer (GOCE) satellite aimed at determining the Earth’s mean gravity field. GOCE delivered gravity gradients containing directional information, which are complicated to use because of their error characteristics and because they are given in a rotating instrument frame indirectly related to the Earth. We compute gravity gradients in grids at 225 km and 255 km altitude above the reference ellipsoid corresponding to the GOCE nominal and lower orbit phases respectively, and find that the grids may contain additional high-frequency content compared with GOCE-based global models. We discuss the gradient sensitivity for crustal depth slices using a 3D lithospheric model of the North-East Atlantic region, which shows that the depth sensitivity differs from gradient to gradient. In addition, the relative signal power for the individual gradient component changes comparing the 225 km and 255 km grids, implying that using all components at different heights reduces parameter uncertainties in geophysical modelling. Furthermore, since gravity gradients contain complementary information to gravity, we foresee the use of the grids in a wide range of applications from lithospheric modelling to studies on dynamic topography, and glacial isostatic adjustment, to bedrock geometry determination under ice sheets.

The Gravity Field and Steady-State Ocean Circulation Explorer (GOCE) was the European Space Agency’s (ESA) first satellite gravity mission that delivered scientific data from November 2009 until October 2013. The aim of the mission was to determine the Earth’s mean gravity field with unprecedented accuracy at a spatial resolution of 100 km or better^1^. The main on-board instrument was the gradiometer that provided gravity gradients, i.e., the Cartesian second spatial derivatives of the gravitational potential^2^. In combination with data from the on-board Global Positioning System (GPS) receiver, the gradients have been used to recover global gravity field models in terms of Stokes coefficients, [e.g.^3^]. These models also allow computing arbitrary quantities of the gravitational potential everywhere on or above the Earth’s surface. Nevertheless, it may be more convenient to use gravity gradients instead of a set of Stokes coefficients, and dedicated regional gravity field solutions may be able to represent the local high-resolution signal more accurately than global models do^4^.

The six GOCE gradients are given in the Gradiometer Reference Frame (GRF), an instrument frame that co-rotates with the satellite. The V_XX_, V_YY_, V_ZZ_ and V_XZ_ gradients have high accuracy in the Measurement Bandwidth (MBW), whereas V_XY_ and V_YZ_ have not and the errors are about two orders of magnitude worse than the accurate gradients. The MBW roughly corresponds to a spatial resolution of 40–750 km half-wavelength. Outside the MBW, however, the gradients are less accurate and may contain systematic errors, and it is not straightforward to use the gradients in the GRF. Nonetheless, the GRF gradients have been used directly or in regional gravity field recovery with different applications^5^,^6^,^7^,^8^,^9^. Alternatively, gradients in the Local North-Oriented Frame (LNOF) are given^10^,^11^,^12^. These gradients are rotated to the LNOF after replacement of the long wavelength signal below the MBW with gradients from a global gravity field model, where also V_XY_ and V_YZ_ are computed from such a model^12^. The LNOF gradients are a compromise between ease of access/application and keeping as much as possible the original GOCE information. The LNOF gradients have been used in regional as well as global applications [e.g.^13^,^14^,^15^,^16^].

The goal of this study is to present global gravity gradient grids at GOCE satellite altitude, which can be used in global and regional geophysical applications. As input we use the accurate gradient data in the GRF where the signal below the MBW has been replaced with information from Gravity Recovery And Climate Experiment (GRACE) that is known to be accurate at these wavelengths [e.g.^17^]. The GRF and LNOF gradients vary tens of kilometres in height above the Earth’s surface^7^. Our global gravity gradient grids have a constant height above the oblate reference ellipsoid calculated in two heights: 255 and 225 km, which correspond to the GOCE nominal and lower orbit phase, respectively. Their advantage over global models in terms of Stokes coefficients is that the gravity gradients are readily available for geophysical modelling and may contain more detailed signal. Their advantage over GRF and LNOF gradients or spherical grids^18^ is that they are at relatively constant height with respect to the Earth’s topography because they are given on homothetic ellipsoids (i.e. with WGS84 eccentricity but different semi-major axis). In addition, they are non-rotating with the satellite in contrast to the GRF gradients and contain only measurement information in the MBW different from the LNOF gradients that contain model information. We study grids at two different heights with the idea that lithospheric models might be better validated using data at different levels and compare the gradient sensitivity with that of gravity, which is conventionally used.

## From GOCE data to gravity gradient grids

We first briefly summarize the data processing to obtain the gravity gradient grids, which should aid in properly interpreting the results. Details are given in the Methods section.

The GOCE satellite collected scientific data from November 2009 until the end of the mission in October 2013. These data were used to compute gravity gradient grids at 225 km and 255 km altitude, which correspond to the satellite perigee height in the nominal and lower orbit phases respectively. Data from the first two and a half months were not used as the accuracy of the vertical gravity gradient is degraded in the initial stages of the mission^8^. Because the GOCE gravity gradients in the GRF are known to be poor at a spatial resolution (half-wavelength) of 750 km and longer^11^ they were high-pass filtered and combined with low-pass filtered gravity gradients derived from GRACE global gravity field models that are known to be accurate at long wavelengths, retaining the accurate GOCE information at shorter wavelengths.

The enhanced gradients were used to compute the grids at the two different altitudes with the help of tesseroids. A tesseroid is a volume element usually defined on a sphere. When a density is assigned to a tesseroid, one can compute its gravitational potential, gravity and gravity gradients^19^. Conversely, given along-track GOCE gravity gradients as observations, one can estimate the unknown density of a tesseroid or the densities of number of tesseroids^20^,^21^. We used as input the four accurate GOCE/GRACE V_XX_, V_YY_, V_ZZ_, V_XZ_ gradients in the GRF. The tesseroid grids are not meant to represent the gravity field at the Earth’s surface, rather we aim to maximize the gravity gradient signal content at 225 km and 255 km. We therefore did not apply regularization and used tesseroids of 55 km × 55 km (0.5° at the equator), which would correspond to spherical harmonic degree L = 360. GOCE-based global gravity field models are regularized and have a maximum spherical harmonic degree of L = 300 or less^22^,^23^. Thus, the tesseroid grids may contain additional gravity gradient signal compared with global models at the expense of increased noise in the grids. The noise in the tesseroid grids is estimated and corrected for as good as possible using the Poisson integral equation (PIE). Briefly, with the PIE one can upward continue gravity functionals given in spherical grids close the Earth’s surface to satellite height. In an iterative procedure the signal at the Earth’s surface is adapted to get a best fit at satellite altitude. The difference between the best fit and the original grids is a measure for the noise in the gradients at satellite altitude. The signal and error content of the original and noise-reduced grids are assessed in the next section.

In principle, one could estimate global gradient grids in a single adjustment. GOCE, however, left two polar caps of a few degrees unobserved and a global adjustment would be unstable and requires regularization. Furthermore, a regional approach has the advantage that one can adjust to the regional signal and error characteristics. A disadvantage of such an approach is that one cannot reliably estimate long wavelength signals beyond the extension of the regional setup. We therefore subtracted from the enhanced gradients reference gradients from the background model GOCO03s^17^ as part of a remove-compute-restore procedure and estimated residual densities in pseudo equal-area blocks of 15° × 15° in a regional approach. A global grid is obtained by a patchwork of the regional grids, after which the background model – containing the long wavelengths – is restored. We computed the gradients on homothetic ellipsoids that have the same eccentricity as the WGS84 ellipsoid and a semi-major axis 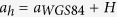, where 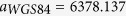 km and *H* is 225 km or 255 km, respectively.

## Results

### Global gravity gradient grids at GOCE satellite altitude

**Figure 1:**
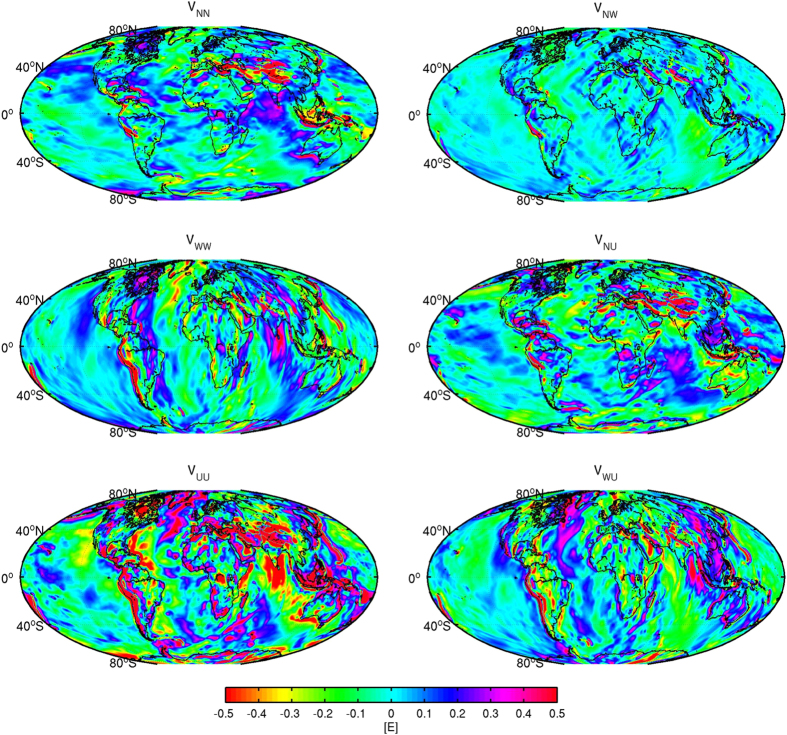
Gravity gradients at 225 km above the Earth’s surface with respect to WGS84. The X-axis points to the north, the Y-axis points west and Z-axis points up and the gradients V_XX_, V_XY_, V_XZ_, V_YY_, V_YZ_ and V_ZZ_ are denoted as V_NN_, V_NW_, V_NU_, V_WW_, V_WU_ and V_UU_ respectively. Colour scales saturated at ± 0.5 E (1 E = 10^−9^ s^−2^). Figure created using the M_Map mapping package^48^.

Global gravity gradient grids at 225 km above the Earth’s surface are shown in Fig. 1, where the gradients in the LNOF are in the North, West, Up (N, W, U) frame, which is the convention adopted for GOCE^12^. Pre-GOCE gravity gradients are visually quite similar^24^, but may contain systematic errors as we will see below. The different gradients have different directional sensitivity. For example, the north-south V_NN_ gradient is sensitive to east-west oriented structures, whereas for the V_WW_ gradient this is the other way around. The radial pointing V_UU_ gradient is isotropic as the Laplace equation holds, that is, V_UU_ = −V_NN_ − V_WW_, and thus observes the strongest gravitational signal. Similar to conventional gravity maps, the gradients although at satellite altitude show remarkable details mainly related to topography and bathymetry. Furthermore, deeper structures are visible as well, which are more clearly seen than in near-surface gravity maps Ref. 16 presented similar figures, although not the complete tensor and along the orbit with its varying height, and showed how satellite gradients relate to deeper mantle sources. In general, the gradients help to delineate the individual features and show clearer the segmentation within and between the oceanic and continental plates.

**Figure 2:**
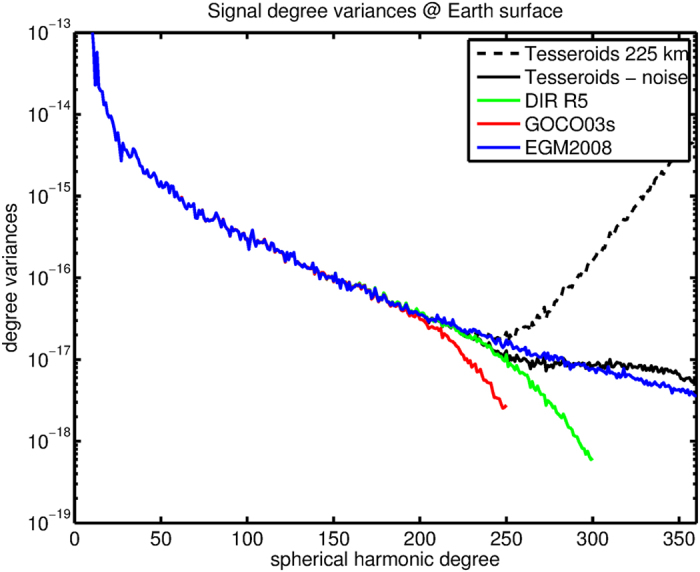
Signal degree variances of global gravity field models and tesseroid grids at the Earth’s surface.

Signal degree variances derived from the global gravity field models EGM2008, GOCO03s and DIR R5 are shown in Fig. 2. EGM2008 is a state-of-the-art high resolution global gravity field model that does not contain GOCE data. Instead, it combines GRACE, terrestrial gravity data and satellite altimeter data^25^, and therefore has in principle full signal variance, which gradually decreases for increasing degree. The GOCE-based satellite models GOCO03s and DIR R5 are truncated at degree L = 250 and L = 300 respectively. In addition, the models employ regularization above degree 180 constraining the highest degrees, which is required when using only satellite data because the downward continuation from satellite altitude to the Earth’s surface amplifies errors especially at high degrees. This explains the lower signal power above spherical harmonic degree L = 200 in both models compared with EGM2008. Consequently, the satellite models are affected by an omission error that depends on the truncation degree and the amount of regularization.

The signal degree variances at the Earth’s surface derived from the tesseroid grids at 225 km with and without noise reduction are shown as well in Fig. 2. If the grids without noise reduction would be evaluated at the Earth’s surface, signal degree variances would become unrealistic above degree L = 250 (dashed black line). Nevertheless, we see that – in contrast to GOCO03s that was used as background model – the grids are close to the full signal power as represented by EGM2008 up to degree L = 250. The signal power of the noise reduced grids stays close to that of EGM2008 up to degree L = 360 (solid black line). Some signal loss is visible roughly between degree L = 220 and L = 280, which is caused by the smoothing effect of the PIE procedure. Above degree L = 300 the denoised grids contain more power than EGM2008, most likely an expression of the increased noise level in the grids at these spatial resolutions. We assessed the omission error in the vertical gravity gradient at 225 km using EGM2008 from L = 361–2190, which gave a signal of 0.1 mE or less. The omission error in the tesseroid grids is therefore small compared with the gravity gradient signal and estimated errors as we will see next.

**Figure 3:**
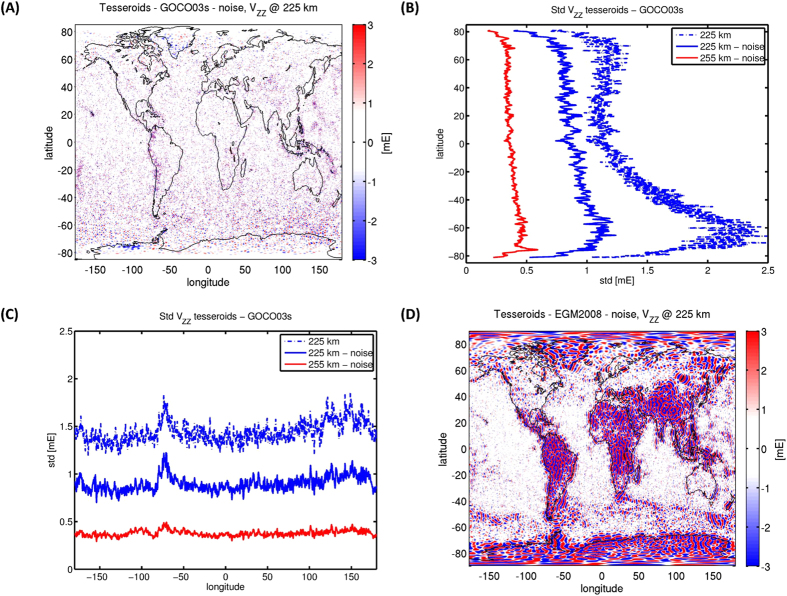
V_ZZ_ differences to GOCO03s and EGM2008 (**A**) Noise reduced tesseroids – GOCO03s @ 225 km; (**B**) Standard deviation as function of latitude @ 225 with and without noise reduction; (**C**) Standard deviation as function of longitude @ 225 km with and without noise reduction; (**D**) Noise reduced tesseroids – EGM2008. Colour scales in (**A**,**D**) saturated at ± 3 mE. Figure created using MATLAB^49^.

The differences between the noise reduced grids and GOCO03s are shown in Fig. 3A for the vertical gravity gradient at 225 km. The differences are a combination of noisy and coherent patterns, which is explained by the low-pass filtering in GOCO03s (truncation at L = 250 as well as regularization) that is largely absent in the tesseroid grids. The coherent patterns consist on the one hand of regions where the omission error in GOCO03s is apparent, and on the other hand of regions, Greenland and West Antarctica, where the difference in reference epoch between GOCO03s (2005.0) and the tesseroid grids (data from 2010–2013) plays a role. Indeed, West Antarctic ice mass imbalance has been determined from a combination of GRACE and GOCE data^6^. Although roughly twice the data amount was used for the tesseroids compared with GOCO03s, it is reasonable to assume that the noisy patterns are mainly caused by the tesseroids as they were much less low-pass filtered. Also note that GOCO03s was used as background model, which means that certain errors of GOCO03s will be contained in the tesseroid grids. At long wavelengths, for example, the grids cannot improve upon the reference model as we perform regional gravity field analysis, and in the Polar Regions, where no GOCE data are available, the tesseroid grids reproduce GOCO03s (roughly above 80° latitude).

Figure 3B shows the standard deviation of the GOCO03s – tesseroid V_UU_ differences as function of latitude. The standard deviation that includes the original tesseroid grids at 225 km (dashed blue line) is small around the equator, increases for higher latitudes and becomes again smaller towards the Polar Regions. In addition, there is a north-south asymmetry. This behaviour can be explained by the GOCE orbit characteristics. The orbit perigee height was located around 15°N^7^ and the orbit height increased towards the north and the south, where the increase to the south was much more prominent. As a result the along-track gradient data are downward continued more at mid latitudes than for low latitudes to produce grids at 225 km above the ellipsoid, and the errors will be amplified more. The standard deviation of the differences decreases for high latitudes, as the data density per square km significantly increases because of meridian convergence and reduces the error in both GOCO03s and the tesseroid grids. If we reduce the noise from the grids we see that the standard deviation of the differences become more homogeneous from north to south (solid blue line in Fig. 3B). At 255 km the standard deviation is even smaller because by upward continuation both signal and noise are reduced (red line in Fig. 3B). Figure 3C shows the standard deviations of the GOCO03s – tesseroid V_UU_ differences as function of longitude. The local maxima for the 225 km and 255 km grids (with and without noise reduction) can be associated with the additional signal that is contained in the grids. If we take the standard deviation of the differences as a measure for the accuracy of the tesseroid grids, then this error is around 1 mE for the denoised grids at 225 km and 0.5 mE or less at 255 km.

There are large differences between EGM2008 and the tesseroid grids (or other GOCE-based information) over the continents in regions where terrestrial gravity data are sparse (Fig. 3D). Also the ocean areas neighbouring areas with poor ground data can be significantly affected as the land error leaks into the ocean. Furthermore, also in coastal areas with presumably good ground data larger differences may occur, for example Southern Norway, which may point to differences in vertical datums that were used for the terrestrial gravity data sets in EGM2008^26^. In addition, the signature of major ocean currents is visible, which is caused by the imperfect separation of geoid and dynamic ocean topography signal from satellite altimetry in EGM2008. This emphasizes the significance of GOCE for improved gravity field determination.

### North Atlantic

**Figure 4:**
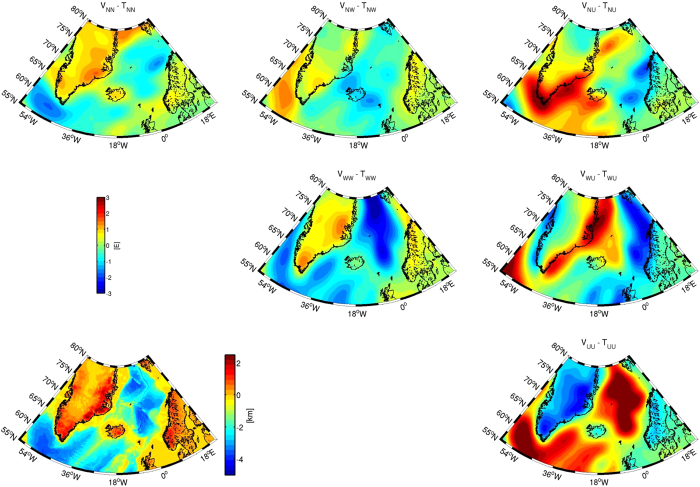
Topographic reduced gravity gradients for the North Atlantic region and topography 50 (lower left). See Methods section for details on reduction. Figure created using the M_Map mapping package^48^.

The gravity gradients in satellite height have the fortunate advantage that they are limited to wavelengths larger than 50 km, which makes them ideal to study the regional crustal or lithospheric setting [e.g. 7,13,14]. As an example, we show in Fig. 4 gravity gradients for the North Atlantic Region reduced for the effect of topography, bathymetry and ice thickness (see Methods for details). As opposed to near-surface or altimetry data that can be used to delineate local features of the spreading ridge and transform faults [e.g.^27^,^28^], the reduced gradients enhance the main structural elements of the area, which are as well reflected in the lithospheric architecture. For example, the UU-component shows the division between the oceanic and continental shelf domains and the transition to the stable cratons of Greenland and Fennoscandia and reflects hereby the changes in lithospheric and crustal thickness [e.g.^29^]. Over Greenland, especially the diagonal gradients show clear changes from the Atlantic coastal area to the interior, which probably reflects changes in lithospheric architecture as a response to ice loading and changing tectonic regime.

**Figure 5:**
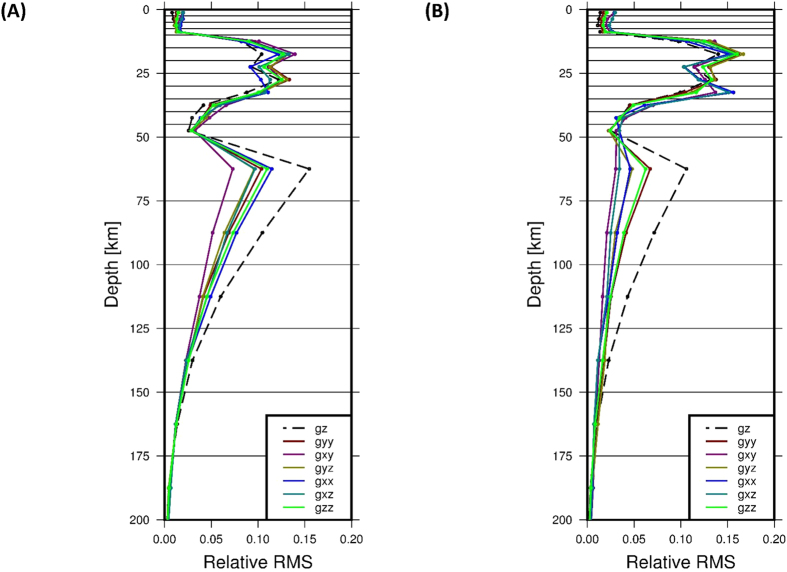
Examples of signal behaviour for lithospheric model of NE Atlantic (**A**) Relative signal for each depth slice for all gravity gradients and the vertical gravity field in 255 km height. (**B**) Relative difference in signal content between calculations in 225 km and 255 km height. In both figures the horizontal lines indicate the thickness of each depth slice.

Ref. 30 developed the theoretical sensitivity kernel for gravity gradients, but of course in practice, the location of sources in depth controls the response function. For example Ref. 16 showed how to potentially retrieve the signal of a subducting slab in the long-wavelength component of the gradients. Previously, the signal for depth slices of a lithospheric-scale model of the North-East Atlantic region has been presented as well [^7^ and supplementary material therein]. For the area, where the transition from a passive margin to the stable Fennoscandian shield dominates the lithospheric architecture, the earlier analysis showed that the gravity components have an individual distinctive depth sensitivity, which is summarized in the left panel of Fig. 5 for gradients at 255 km. The relative power or signal content shows three distinctive maxima for the North-East Atlantic region. They can be interpreted to reflect the density contrast between the oceanic crust and mantle, the high-density continental lower crust and the continental crust-mantle transition. The depth sensitivity differs from gravity gradient to gravity gradient – most notably from the vertical V_UU_ to the mixed horizontal V_NW_ gravity gradient – and this can be exploited in geophysical modelling to place masses with higher confidence in the lithospheric column. Instead of adjusting a model to a single component, the use of all tensor components may limit model uncertainties, even though the ambiguity in the solution remains. We also see that the normal, vertical gravity is more sensitive to deeper sources. Thus, a model optimised to gravity gradients consequently helps to estimate the non-lithospheric sources in the underlying mantle, which might be associated to dynamic topography^31^.

An even more controlled modelling set-up can potentially be achieved by using the data from both heights (225 km and 255 km). Even though the absolute amplitude difference between the two grids is small, the distribution of the relative signal power is again different for the individual components (Fig. 5, right panel). This implies that, instead of adjusting a model to a single data set, an optimum model has to be adjusted to multiple data sets, which helps to increase confidence in the interpretation. While this doesn’t overcome the non-uniqueness of the gravity method, Ref. 32 have shown that the uncertainty in geological interpretations can be reduced, when considering the full signal of the gravity tensor and the field.

## Discussion

In the computation of the gravity gradient grids we chose an almost constant height with respect to the reference ellipsoid. This minimizes the distance to the Earth’s surface as compared with spherical grids or the original height of the GOCE data. This is important in order to reduce the errors caused by planar earth approximation^5^. Furthermore, we use a regional approach and we have to rely on the long wavelength information of the background global gravity field model (GOCO03s). Possibly the tesseroid grids can be improved by using more recent GRACE/GOCE global models, but their accuracy needs to be carefully assessed.

The GOCE gravity gradients will help to establish more realistic models of the lithosphere and upper mantle. This is interesting for a vast series of applications, here foremost to mention are studies on dynamic topography or the composition of the upper mantle. Estimates of dynamic topography as induced by mantle convection rely on separation of the lithospheric and sub-lithospheric gravity field. A separation in the spectral or spatial domain is not straightforward as different effects superpose each other [e.g.^33^]. Therefore, reliable lithospheric models have to be established to be able to identify the sub-lithospheric contribution. This need has been identified in the geophysical community and resulted for example in the lithospheric model Litho1.0^34^. This velocity model cannot directly be transformed into a reference density model and here the GOCE gravity gradients potentially will play an important role. For airborne data, Ref. 35 investigated the information content gravity gradients carry and concluded that for shallow sources the vertical gradient is the best choice, whereas for deeper sources differential curvature components might be the best choice. The satellite grids presented here provide the possibility to test such ideas and concepts in inverse and forward modelling.

Precise knowledge of the bedrock geometry, ice sheet thickness and surface topography are important to better understand Greenland and Antarctic ice sheet mass fluxes and associated dynamical behaviour. Gravity data derived from GOCE have been used to validate Antarctic bedrock geometry at a spatial resolution of roughly 90 km^36^. The gravity gradient grids allow extending such analysis to the Greenland ice sheet and other regions. We speculate that using gradients instead of gravity results in a more reliable validation or even estimate of bedrock geometry, possibly at a resolution down to 50 km.

One of the largest uncertainties in the determination of Antarctic ice-mass balance is glacial isostatic adjustment (GIA)^37^, that is, the changes in the Earth’s shape and gravitational field caused by slow viscous mantle flow as a consequence of the Earth’s response to its deviation from gravitational hydrostatic equilibrium caused by ice melt since the last glacial maximum. The large GIA uncertainty for Antarctica is a consequence of the poor data constraint of, e.g., the effective elastic thickness and other parameters of GIA models^38^. One of the GOCE mission goals was that GOCE-based gravity information might aid in better constraining GIA models and the reduction of the uncertainty of Antarctic ice-mass imbalance and its contribution to global sea level rise^24^. In particular, Ref. 39, assess that gravity data from GOCE can be used to estimate the elastic lithospheric thickness in regions where it is greater than 15 km, and where large topographic loads produce large gravity anomalies. This is typically the case for Antarctica; see^38^ and the Supplementary Material. We believe that, in addition to gravity, the gravity gradient grids at satellite altitude may help to better resolve these parameters.

Finally, as a result of our study, unique, new and accurate global gravity gradient grids at GOCE satellite altitude are made available to the geophysical community. This new gravitational potential field data source has been used here and in other feasibility studies^7^,^13^,^14^ in a novel way to assess its potential and limitations for lithospheric modelling. Based on these initial experiences it can be expected that these grids can contribute to the future development of full 3D or even 4D Earth models. These demanding future modelling developments will certainly benefit from joint analysis of complementary geometric (topography, seismic) and potential field related satellite, airborne, *in-situ* (gravity and magnetic) data and laboratory results. A joint analysis goes beyond the scope of our study but the data provided and the results obtained can be regarded as a necessary step in this direction.

## Methods

The nominal phase of the GOCE mission lasted until July 2012 in which the satellite had a perigee height of 255 km above the Earth. From August 2012 onward a number of orbit lowerings were carried out until the satellite had a perigee height of 225 km in May 2013. Thus, the so-called lower orbit phase contains data from August 2012 until October 2013, and the nominal phase from November 2009 until July 2012. The data from the nominal and lower orbit phases are used to compute grids at 225 km and 255 km. Data from the first two and a half months were not used as the accuracy of the vertical gravity gradient is roughly 40% worse in the initial stages of the mission compared with the rest of the data^8^.

**Figure 6:**
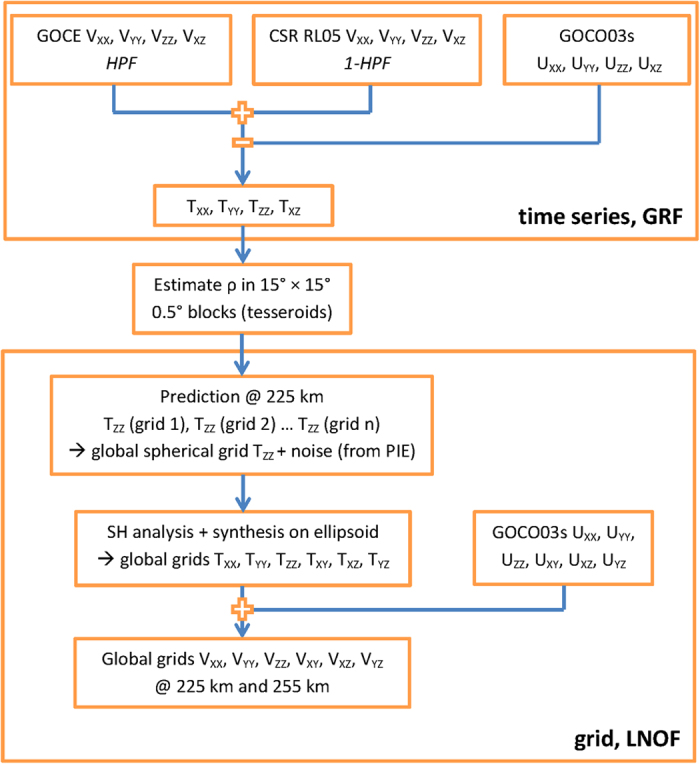
Flowchart of the computational procedure to arrive at gravity gradient grids.

The computational procedure is summarized in Fig. 6, with the following steps:

1. Compute enhanced gradients from a combination of GOCE data at high spatial resolution and GRACE-based gradients for low spatial resolution. The GOCE data are high-pass filtered (HPF), the GRACE gradients are low-pass filtered (1-HPF). Above the GOCE MBW (spatial resolution <40 km) additional filtering is applied to suppress noise.
2. We use a remove-compute-restore technique, which minimizes, e.g., edge effects in the estimated regional grids. Gradients derived from GOCO03s are used to reduce the enhanced gradients.
3. Estimate residual densities in 0.5° tesseroids from T_XX_, T_YY_, T_ZZ_, and T_XZ_.
4. Predict T_ZZ_ in a global spherical grid at 225 km above the reference sphere from a patch work of regional grids, and estimate the noise using the Poisson integral equation. A spherical grid is used here because both the tesseroids and the PIE are formulated in spherical coordinates. Furthermore, this allows an exact representation – up to rounding errors – in terms of spherical harmonic coefficients.
5. Use spherical harmonic synthesis to compute gradient grids for all gradients @ 225 km and 255 km above the ellipsoid and add back GOCO03s (the restore step).

We first discuss in more detail the input gravity gradients that are obtained by combining GOCE gradient data with existing gravity field information from global gravity field models at long wavelengths to circumvent the systematic errors in the GOCE gradients there. Next, we present a method that uses tesseroids for regional gravity field recovery and a patchwork of regional solutions that build the global gradient grids. Further the reduction of the grid errors through the use of the Poisson integral equation is discussed. Finally, we discuss the topographic mass reduction and the lithospheric sensitivity computation.

### Enhanced along-track gravity gradients

**Figure 7:**
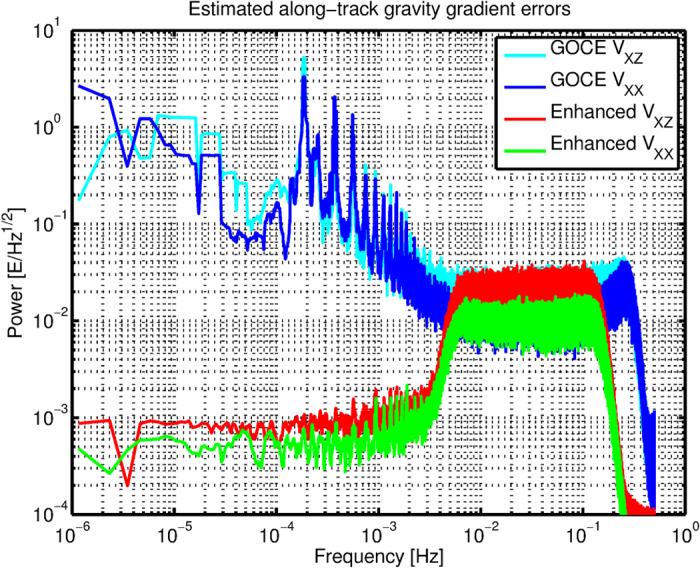
Spectral density of estimated V_XX_ and V_XZ_ along-track gravity gradient errors for a 10 day period.

The GOCE gravity gradients are given in an instrumental frame, the so-called gradiometer reference frame (GRF), which co-rotated with the satellite in orbit^11^. Four of the six gravity gradients (V_XX_, V_YY_, V_ZZ_ and V_XZ_) have high accuracy in the measurement bandwidth (MBW) between 5 mHz and 100 mHz – or a spatial resolution of 750 km to 40 km – with error increase above and below the MBW^11^,^12^. Estimated errors are exemplarily shown for the V_XX_ and V_XZ_ gradients in Fig. 7 (blue and cyan lines). Shown are the differences between GOCO03s and the GOCE gradients for a period of 10 days starting 22 December 2011 at 00:00:00 UTC and ending 31 December 2011 at 23:59:59 UTC. As 1.5 years of data are averaged in GOCO03s, the differences are mainly caused by the single point errors in the along-track data. The error levels of V_YY_ and V_ZZ_ are roughly the same as for V_XX_ and V_XZ_ respectively. The large errors at *k*·1.9·10^−4^ Hz (*k* an integer) are related to the orbital frequency of the GOCE satellite in combination with the Earth’s flattening and the orbital eccentricity, which generate gradient signals with large amplitude. Because of imperfections in the gradiometer instrument, e.g., part of the V_ZZ_ signal leaks to V_XX_^10^,^40^.

We computed enhanced along-track gradients in the GRF by replacing the original GOCE signal below the MBW with that from existing GRACE-based global gravity field models^41^ that are known to be very accurate at long wavelengths. Specifically, we computed gravity gradients in the GRF from the global gravity field models and low-pass filtered these with a cut-off frequency of 5 mHz. The GOCE gravity gradients were filtered with the complement of the low-pass filter, and the sum of the low-pass filtered model and high-pass filtered GOCE gradients gives the enhanced gravity gradients in the GRF. An additional filtering above the GOCE MBW suppresses noise. The estimated errors of the enhanced along-track gradients are shown in Fig. 7 in green and red for V_XX_ and V_XZ_ respectively. In the MBW the GOCE original gravity gradient signal and error are kept, whereas below the MBW the errors are small because of the high accuracy of the GRACE information. Although highly accurate, this information has mainly 1D character and including more and more GRACE data for higher frequencies would lead to the occurrence of the typical GRACE stripes error pattern^21^.

### Tesseroid patchwork

A tesseroid is a volume element usually defined on a sphere. When a density is assigned to a tesseroid, one can compute its gravitational potential, gravity and gravity gradients^19^. Conversely, given along-track GOCE gravity gradients as observations, one can estimate the unknown density of a tesseroid or the densities of number of tesseroids^20^,^21^. We used as input the four accurate GOCE/GRACE V_XX_, V_YY_, V_ZZ_, V_XZ_ gradients in the GRF. The linear observation equation system is solved using least squares where the a priori weights are adjusted using variance-component estimation^42^. We used the GOCO03s global gravity field model^17^ to compute along the GOCE orbit reference gradients in the GRF with which the measured along-track gravity gradients are reduced.

We estimated residual densities in pseudo equal-area blocks of 15° × 15° in a regional approach. The blocks are defined at the equator and shifted around the Earth maintaining their size. This avoids potential problems for high latitudes that might occur when using equiangular tesseroids. The tesseroids have a resolution of 0.5° at the equator and there are therefore 900 tesseroids for each regional solution. The tesseroids are located at 100 km above the surface, which avoids downward continuation to the Earth’s surface and associated numerical instabilities. Locating the tesseroids even closer to the observation points may give numerical instabilities as well^43^. A global grid is obtained by a patchwork of regional 15° × 15° grids shifted by steps of 10° in latitude and longitude. The minimum overlap between adjacent grids is therefore 2.5°.

The estimated densities are used to predict the vertical gravity gradient T_ZZ_ in 225 km or 255 km altitude in the local-north-oriented frame (LNOF). The vertical gradients are interpolated on an equiangular 0.2° grid in 255 km (or 225 km) using natural neighbour interpolation. To avoid edge effects, blocks of 12° × 12° are used, discarding 1.5° on all sides. Next, Stokes coefficients are estimated from the interpolated T_ZZ_ values using spherical harmonic analysis, which are finally used to compute all six gravity gradients in the LNOF frame at a height of 255 km (or 225 km) above the reference ellipsoid. More precisely, we computed the gradients on a homothetic ellipsoid that has the same eccentricity as the WGS84 ellipsoid and a semi-major axis 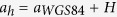, where 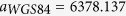 km and *H* is 225 km or 255 km, respectively. In practice this means that the height *h* above the WGS84 ellipsoid slightly varies from equator to the poles. For the lower grids, for example, the height is *h* = 225 km at the equator and 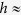 224.25 km at the poles. In the restore step the GOCO03s contribution is added back. The polar gaps without GOCE data were not covered with tesseroids and the grids reproduce GOCO03s above 83°.

### Downward continuation and error estimation

In order to reduce the high-frequency noise that remained in spherical gradient grids for T_ZZ_, we made use of the iterative strategy based on the Poisson integral equation (PIE) as described in^44^. In this strategy, a global grid was iteratively downward continued using 4500 iterations. Basically, the procedure provides the signal at two altitudes since it employs both the upward and downward continuation in each iteration. However, we are interested only in the noise estimate at the satellite altitude where the noise is obtained from a difference of the final iteration and the original (input) grid. The high-frequency information (noise) is missing in this final iteration because the Poisson kernel acts as a low-pass filter, which reproduces only those frequencies that are in agreement with the altitude difference used. This difference was set to be 225 km as this value represents a realistic distance from the GOCE satellite to the Earth masses. The obvious advantage of this iterative procedure is that the error estimate can be found without using any a priori signal.

### Topographic mass reduction and lithospheric sensitivity

Figure 4 and the supplementary images show gravity gradients after topographic mass reduction. The topographic mass reduction has been done using the spherical harmonic model RWI_TOPO_2012 for rock, water and ice density^45^, where we used a maximum spherical harmonic degree of L = 360 to be consistent with the gravity gradient resolution. This global correction enhances the signal of the internal structure of the Earth and is the equivalent to a Bouguer gravity anomaly. RWI_TOPO_2012 is complete to spherical harmonic degree and order 1800 and applies a three-layer decomposition of the topography using the 5′ × 5′ topographic database DTM2006.0^46^. Rock, water, and ice masses are separately modelled with layer-specific density values of 2670 kg m^−3^, 1000 kg m^−3^, and 920 kg m^−3^ for rock, water and ice respectively.

The relative gravity gradient signal content has been calculated by dividing the signal of each individual depth slice by the total signal from all slices. The North-East Atlantic model has an extension to 300 km depth with a horizontal resolution of 0.1° in East and North direction. The depth slices are selected with respect to the depths and uncertainties of the individual model geometries. For the upper 10 km depth slices of 2.5 km are used, whereas 5 km thick depth slices are used from 10 km to 50 km depth. This is in line with the uncertainties of seismic estimates, which are at least ±2 km for the region^47^. From 50 km to 300 km depth the gravity gradients are calculated for depth slices of 25 km thickness, similar to the vertical resolution of seismic tomography (~30 km). The signal has been calculated for the spherical geometry with the software Tesseroids^43^. For more details see^7^.

## Additional Information

**Data availability**: The gravity gradient grids at 225 km and 255 km height are available from https://earth.esa. int/web/guest/missions/esa-operational-eo-missions/goce or via anonymous ftp from ftp://ftp.dgfi.tum.de/ pub/goce2/Gradient_Grids/. A MATLAB-tool is provided to extract, from the global grids, grids in regions of interest. In addition, topographic gravity gradient grids at the same altitudes are available from these websites. The topographic reduced gravity gradient grids in the Supplementary Information have been computed using the difference between the GOCE gravity gradients grids and the topographic gradient grids.

**How to cite this article**: Bouman, J. *et al.* Satellite gravity gradient grids for geophysics. *Sci. Rep.* **6**, 21050; doi: 10.1038/srep21050 (2016).

## Supplementary Material

Supplementary Information
