# Supplementary material for: Satellite gravity gradient grids for geophysics
Source: Sci Rep. 2016 Feb 11;6:21050. doi: 10.1038/srep21050 (PMC4750030; doi:10.1038/srep21050)

Supplementary Material
to "Satellite gravity gradient grids for geophysics"

Johannes Bouman[1], Jörg Ebbing[2], Martin Fuchs[1], Josef Sebera[3,4], Verena Lieb[1], Wolfgang Szwillus[2], Roger Haagmans[5], Pavel Novak[6]

1: Deutsches Geodätisches Forschungsinstitut der Technischen Universität München (DGFI-TUM)
2: CAU Kiel, Kiel, Germany
3: Astronomical Institute of the Czech Academy of Sciences, Ondrejov, Czech Republic
4: Research Institute of Geodesy, Cartography and Topography, Zbidy, Czech Republic
5: ESA-ESTEC, Noordwijk, the Netherlands
6: UWB, Plzeň, Czech Republic

Contains:

- Validation of regional gravity field recovery using MSR
- Spherical and ellipsoidal LNOF
    - LNOF – Local North Oriented Frame (spherical)
    - LNOF – Local North Oriented Frame (ellipsoidal)
    - Transformation from spherical to ellipsoidal LNOF
    - Gradient differences ellipsoidal normal and radial direction
- Zooming in on gravity gradient grids around the world @ 225 km altitude
- Gravity gradient grids @ 225 km altitude with topographic reduction

The figures in this supplement were created using the M_Map mapping package. 48. R Pawlowicz (2014). M_Map: A mapping package for Matlab. UBC Department of Earth and Ocean Sciences, Vancouver, Canada. URL http://www.eos.ubc.ca/~rich/map.html

Validation of regional gravity field recovery using spherical basis functions

The tesseroid grids were validated with an alternative method: We applied a regional modeling approach using spherical basis functions (SBF) [2] based on series expansions in terms of Legendre polynomials up to spherical harmonic degree L = 560. The related unknown scaling coefficients were estimated by relative weighting of the six GOCE/GRACE gravity gradients, composing each a separate observation group, using variance component estimation. The less accurate gradients V_{XY} and V_{YZ} are included here and get low relative weights compared with the accurate gradients. In advance, the GOCE/GRACE observations were reduced by the reference model GOCO03S up to degree L = 60. Rotating the observation equations for the analysis process into the GRF enables the use of the original GOCE/GRACE gravity gradients [1]. We estimated the coefficients up to degree L = 560, and multiplied them in the synthesis with Blackman SBFs, where the signal is represented up to degree L = 511, but smoothed between degree L = 256 and 511 to avoid erroneous edge effects. We located them finally in the LNOF on a regular grid at mean orbit height of 225 km.

Differences SBF (up to degree L = 511) – GOCO03s (L = 250) are shown in Figure S1 for V_{NN}, V_{WW}, and V_{UU} for a region east of the Philippines. The differences are a few mE at 225 km height, comparable with the case for the tesseroid grids, and we see that the SBF grids add the same spatial detail to GOCO03s. The differences between tesseroids and SBFs are small and generally below 1 mE, see the right column in Figure S1. We therefore conclude that the SBF and tesseroid grids are in good agreement with each other.

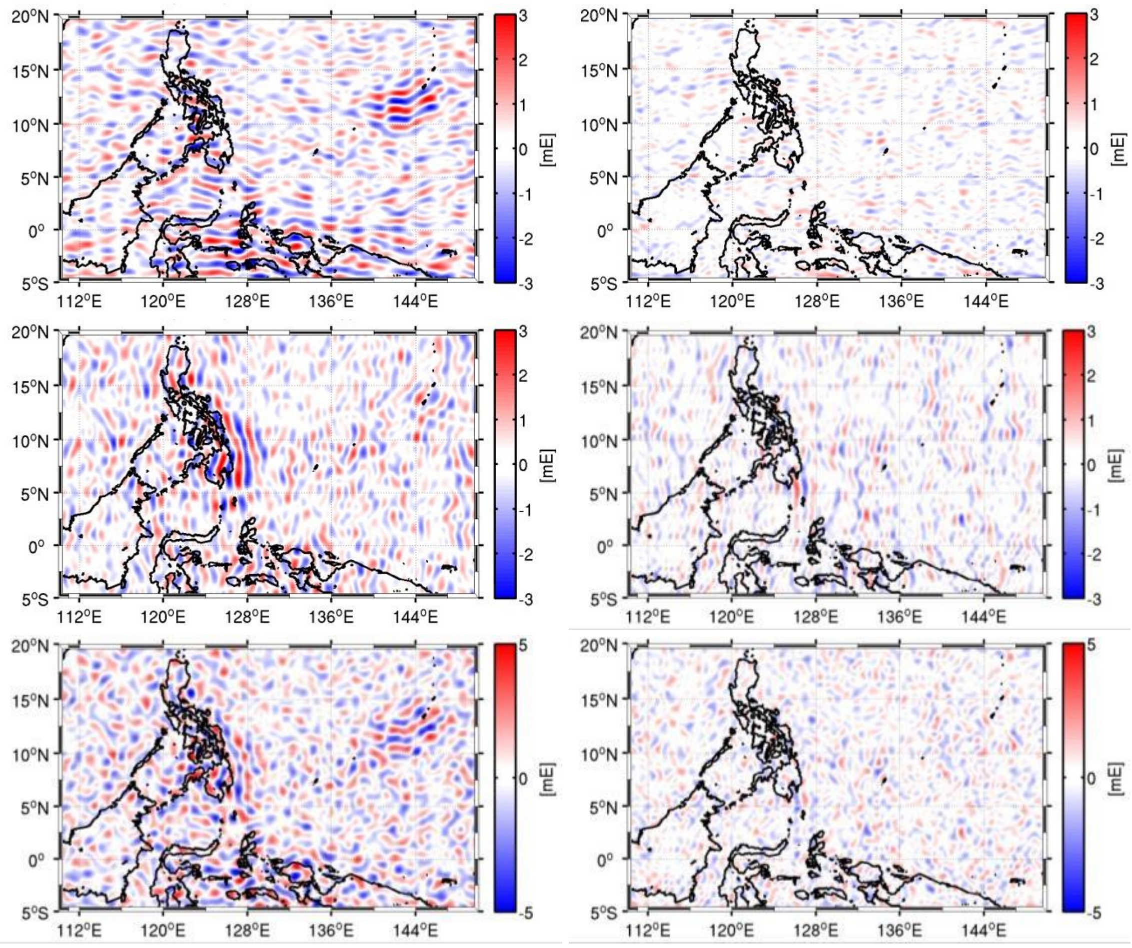

Figure S1: Gravity gradient differences SBF – GOCO03s (left column) and tesseroids – SBFs (right column) at 225 km above the Earth's surface with respect to WGS84. First row: T_{XX}, second row: T_{YY}, third row: T_{ZZ}

References

1. Lieb, V., Bouman, J., Dettmering, D., Fuchs, M. J. & Schmidt M. Combination of GOCE gravity gradients in regional gravity field modelling using radial basis functions in *International Association of Geodesy Symposia*, Chapter 51, Springer, doi: 10.1007/1345_2015_71 (2015).
2. Schmidt, M. et al. Regional gravity modeling in terms of spherical base functions. *J. Geodesy* **81**, 17-38, doi: 10.1007/s00190-006-0101-5 (2007).

Spherical and ellipsoidal LNOF

Grids of gravity gradients are provided in the Local North-Oriented Frame (LNOF) above the ellipsoid. The gradient grids are given on a homothetic ellipsoid that has the same eccentricity as the WGS84 ellipsoid and a semi-major axis $a_H = a_{WGS84} + H$, where a_{WGS84} = 6378.137 km and H is 225 km or 255 km. It should be noted that different LNOF definitions are possible, which depend on whether the vertical axis is taken along the direction of the ellipsoidal normal or along the direction of the spherical normal. The GOCE gravity gradient grids are given in the spherical LNOF but can easily be transformed to the ellipsoidal LNOF. The exact definitions are given below as well as an assessment of the differences. How the transformation can be done is shown as well.

LNOF – Local North Oriented Frame (spherical)

The Local North Oriented Frame (LNOF) is a right-handed North-West-Up frame with the X-axis pointing North, the Y-axis pointing West and the Z-axis Up.

- The origin O_{LNOF} is located at a grid point,
- Z_{LNOF} is defined as the vector from the geocenter to the origin O_{LNOF} (grid point), pointing radially outward,
- Y_{LNOF} is parallel to the normal vector to the plane of the geocentric meridian of the satellite center of mass, pointing westward,
- X_{LNOF} is parallel to the normal vector to the plane defined by Y_{LNOF} and Z_{LNOF} and forms a right-handed system.

(X, Y, Z) is therefore (N, W, U) = North, West, Up. In geocentric latitude and East longitude (ϕ, λ) the 3 axes are defined as follows:

$$Z_{LNOF} = \begin{pmatrix} \cos\phi\cos\lambda \\ \cos\phi\sin\lambda \\ \sin\phi \end{pmatrix}; Y_{LNOF} = \begin{pmatrix} \sin\lambda \\ -\cos\lambda \\ 0 \end{pmatrix}; X_{LNOF} = \begin{pmatrix} -\sin\phi\cos\lambda \\ -\sin\phi\sin\lambda \\ \cos\phi \end{pmatrix}$$

LNOF – Local North Oriented Frame (ellipsoidal)

The ellipsoidal LNOF uses geographic longitude λ and latitude φ to define these grids and we have

$$Z_{LNOF} = \begin{pmatrix} \cos\varphi\cos\lambda \\ \cos\varphi\sin\lambda \\ \sin\varphi \end{pmatrix}; Y_{LNOF} = \begin{pmatrix} \sin\lambda \\ -\cos\lambda \\ 0 \end{pmatrix}; X_{LNOF} = \begin{pmatrix} -\sin\varphi\cos\lambda \\ -\sin\varphi\sin\lambda \\ \cos\varphi \end{pmatrix},$$

which thus slightly differs from the spherical definition because geocentric and geographic latitude are slightly different.

Transformation from spherical to ellipsoidal LNOF

The relation between geocentric latitude ϕ and geographic latitude φ is

$$\varphi = \operatorname{atan}\left(\frac{\tan\phi}{1-e^2}\right)$$

with the inverse relation

$$\phi = \operatorname{atan}\big((1-e^2)\tan\varphi\big)$$

where e is the eccentricity.

In each grid point one can then rotate the gradient tensor from the spherical LNOF to the ellipsoidal LNOF using

$$\begin{pmatrix} V_{XX} & V_{XY} & V_{XZ} \\ V_{XY} & V_{YY} & V_{YZ} \\ V_{XZ} & V_{YZ} & V_{ZZ} \end{pmatrix}_e = R_2(\varphi-\phi)\begin{pmatrix} V_{XX} & V_{XY} & V_{XZ} \\ V_{XY} & V_{YY} & V_{YZ} \\ V_{XZ} & V_{YZ} & V_{ZZ} \end{pmatrix}_s \cdot R_2^T(\varphi-\phi)$$

with

$$R_2(\varphi-\phi) = \begin{pmatrix} \cos(\varphi-\phi) & 0 & -\sin(\varphi-\phi) \\ 0 & 1 & 0 \\ \sin(\varphi-\phi) & 0 & \cos(\varphi-\phi) \end{pmatrix}.$$

Given the geographic latitude φ of the grid points, the geocentric latitude ϕ can be computed and the gradient tensor can be rotated.

Gradient differences ellipsoidal normal and radial direction

When computing gravity gradients in a regular ellipsoidal grid, the direction of the ellipsoidal normal and the direction of the spherical normal are not equal. To assess the these differences, we computed the spherical coordinates of a grid with 0.2° spacing at 225 km above the ellipsoid. The North-East Atlantic region was used for the test computations. The figure below shows the difference between the two latitudes.

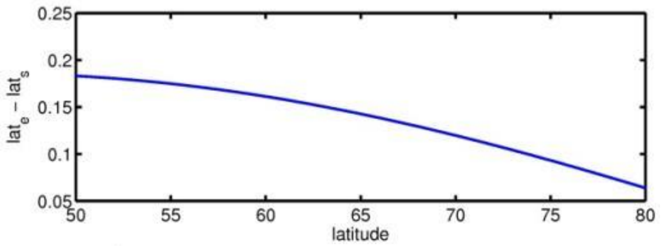

Next we computed gravity gradients at 225 km above the ellipsoid for the NEA region using GOCO03s with respect to GRS80. The lower left panel in the figure below shows the vertical gravity gradient, which has maximum amplitude of about 0.6 E. The other panels show the difference between the gradients in the LNOF (ellipsoidal) and LNOF (spherical). Because the rotation is around the y-axis (east-west) the T_{YY} differences are zero. Largest differences occur for T_{ZZ} and these are 3 mE or less. Thus, although there may be correlation between these differences and the signal, the maximum amplitude is small, but may be above the accuracy of the gradient grids.

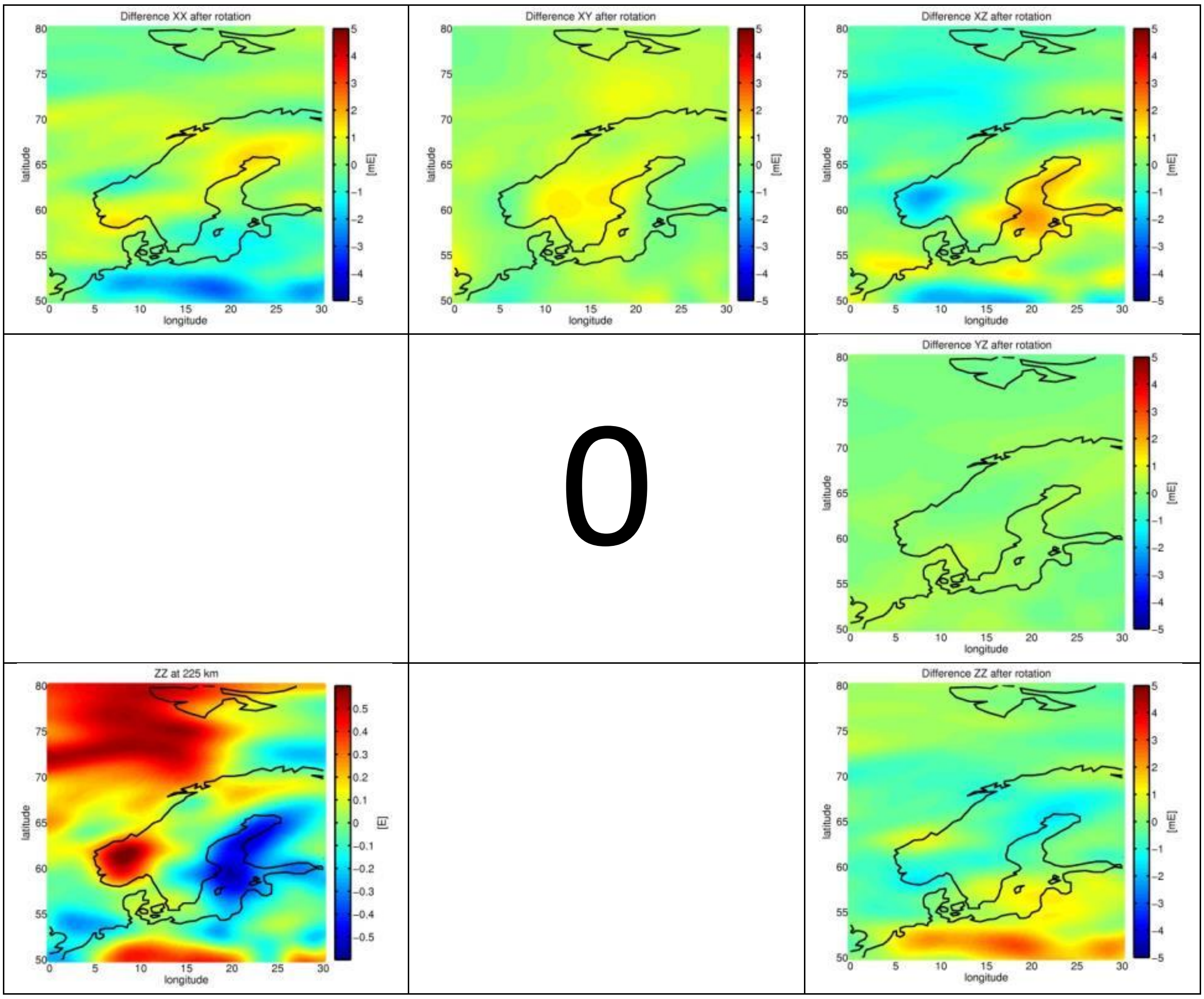
Difference XX after rotation
Difference XY after rotation
Difference XZ after rotation
0
Difference YZ after rotation
ZZ at 225 km
Difference ZZ after rotation
latitude
longitude
[mE]
[E]

Zooming in on gravity gradient grids around the world @ 225 km altitude

The gravity gradient signal at 225 km altitude is shown for longitude-latitude blocks of 60° × 30° for longitudes -180° ≤ λ ≤ 180° and latitudes -75° ≤ ϕ ≤ 75°. The North and South Pole are shown separately. The gradient signal with respect to the WGS84 reference ellipsoid is shown in the order as shown in the table below, starting in the south-west. The colour scale in each region and for each gradient is adapted to the min/max values in that region. Topography and bathymetry contour lines are shown every 1000 m and were derived from ETOPO1 [47]. The Lambert projection is used for the patches centred at ϕ = ±60°, a stereographic projection is used for the North and South Pole, and all other patches use the Mercator projection.

V_{XX}	V_{XY}
V_{YY}	V_{XZ}
V_{ZZ}	V_{YZ}

The geolocation of all patches is shown in the figure below.

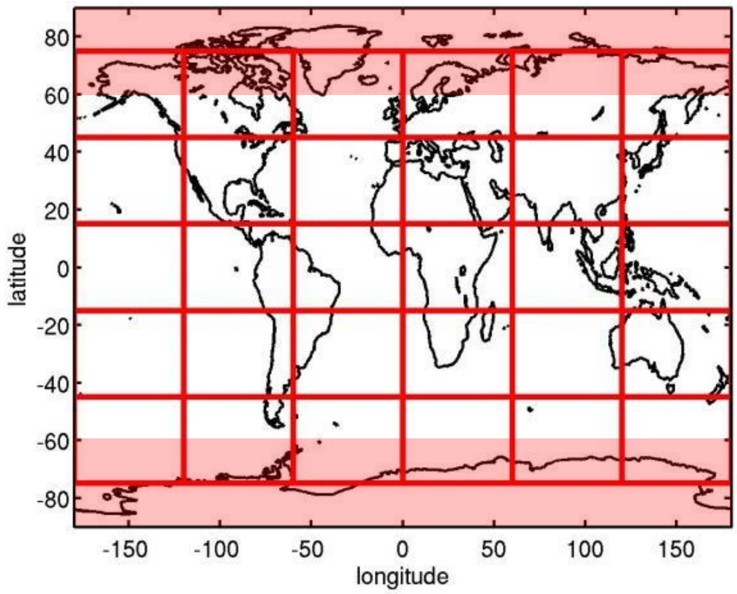

-180° ≤ λ ≤ -120°, -75° ≤ φ ≤ -45°

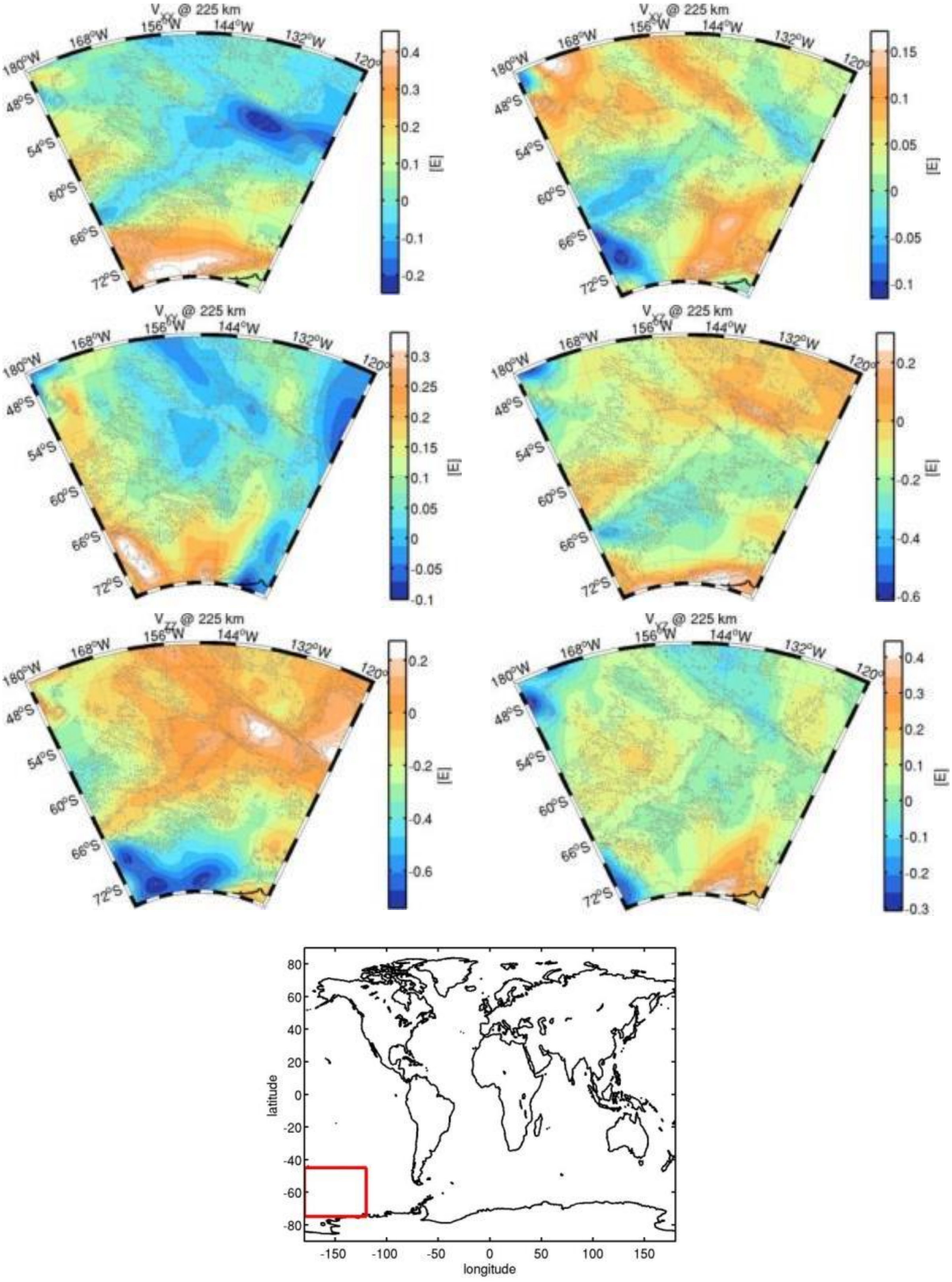

$-120° \leq \lambda \leq -60°, -75° \leq \varphi \leq -45°$

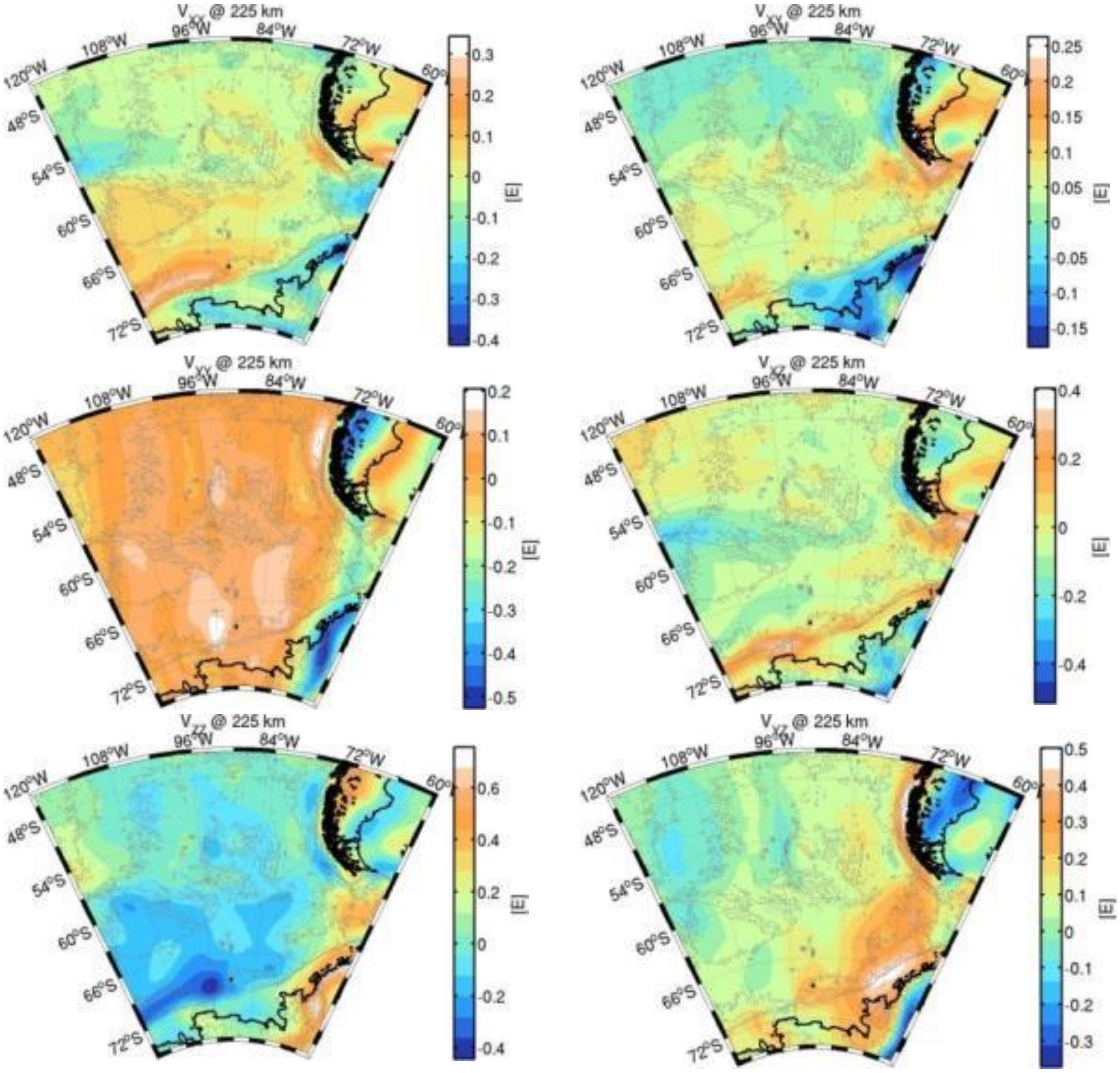

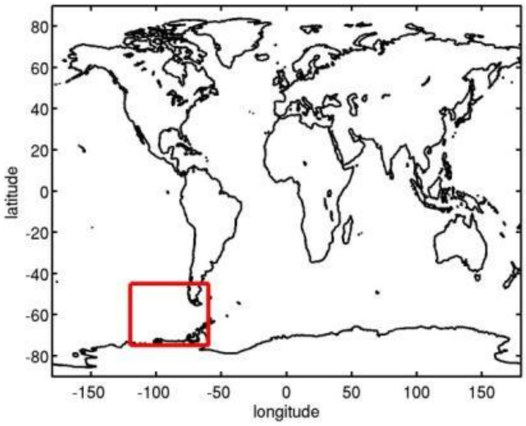

-60° ≤ λ ≤ 0°, -75° ≤ φ ≤ -45°

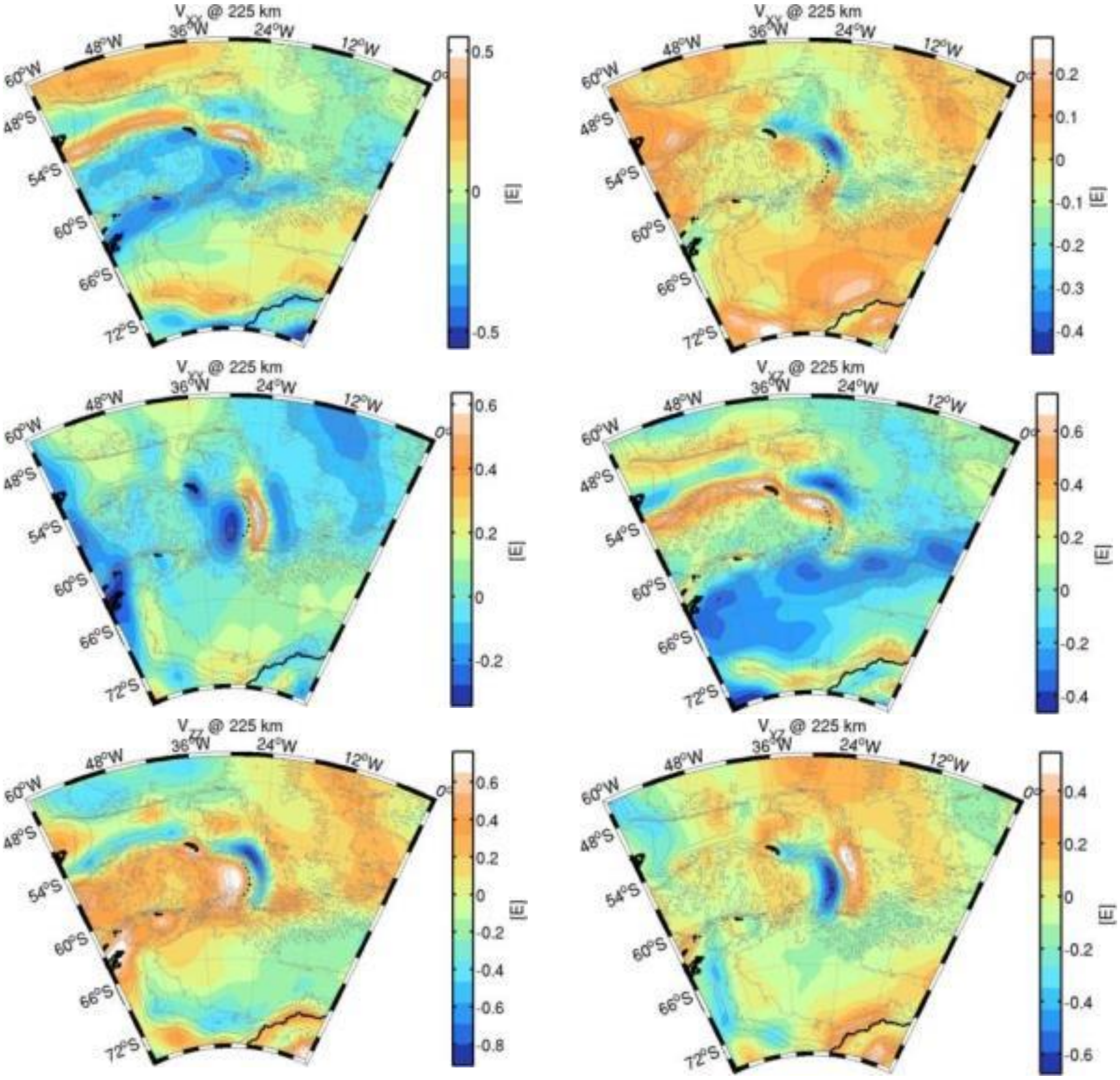
Vxx @ 225 km
Vxy @ 225 km
Vyy @ 225 km
Vxz @ 225 km
Vzz @ 225 km
Vyz @ 225 km
60°W
48°W
36°W
24°W
12°W
0°
48°S
54°S
60°S
66°S
72°S
[E]

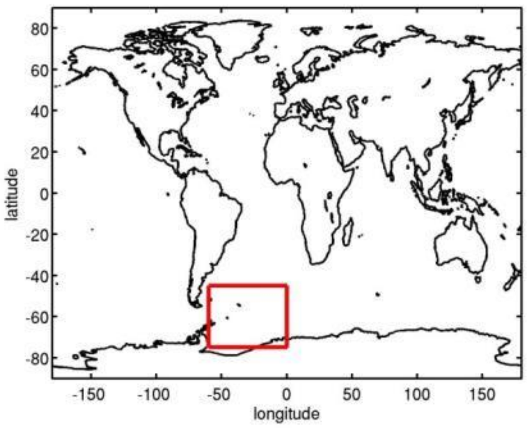
latitude
longitude
80
60
40
20
0
-20
-40
-60
-80
-150
-100
-50
0
50
100
150

0° ≤ λ ≤ 60°, -75° ≤ φ ≤ -45°

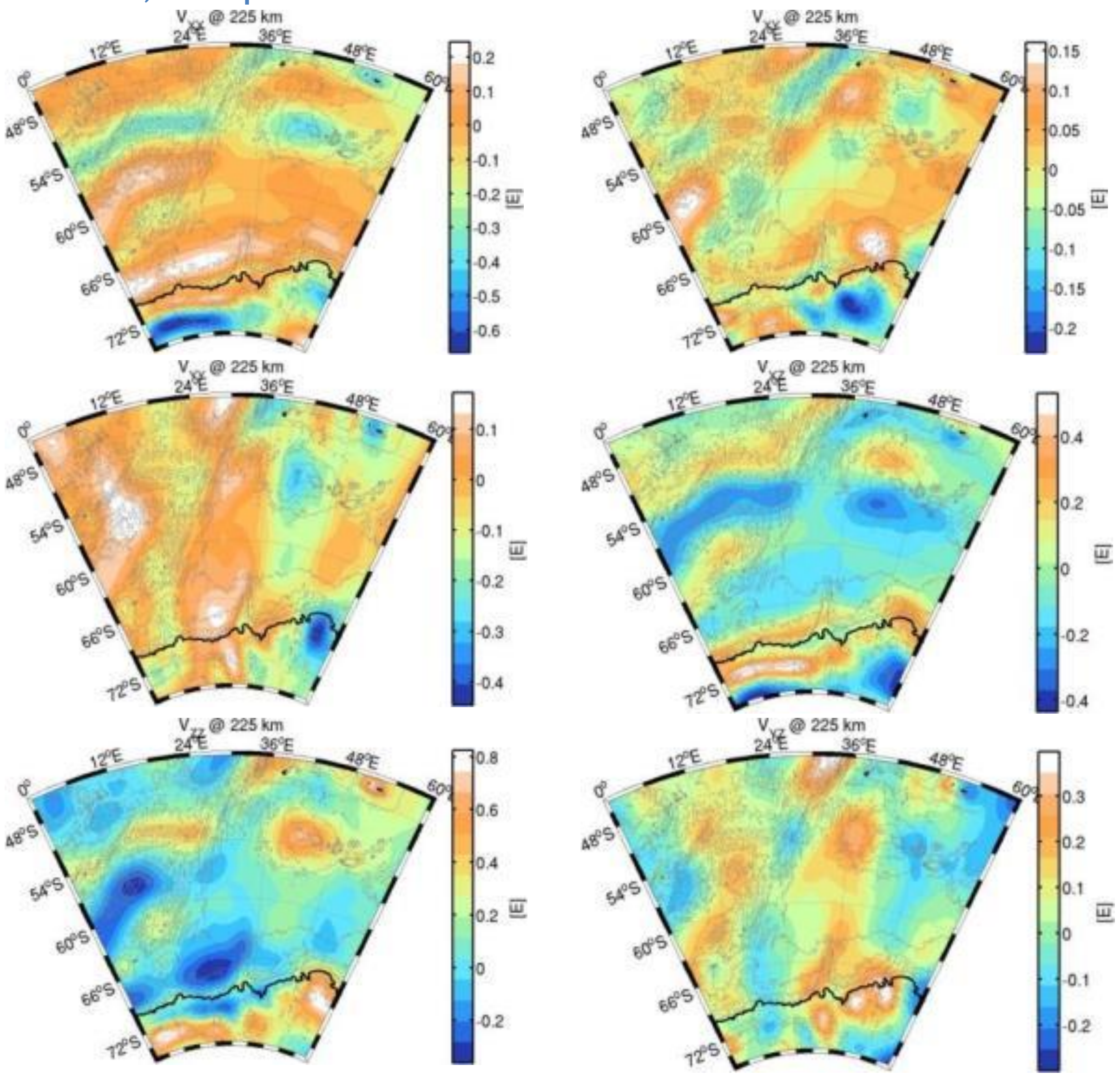

@ 225 km
0°
12°E
24°E
36°E
48°E
60°
48°S
54°S
60°S
66°S
72°S
[E]

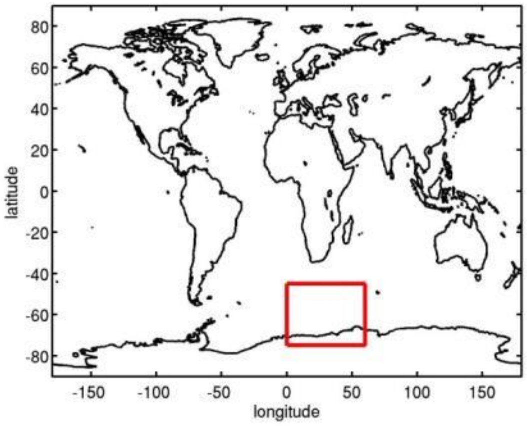

latitude
longitude
80
60
40
20
0
-20
-40
-60
-80
-150
-100
-50
0
50
100
150

60° ≤ λ ≤ 120°, -75° ≤ φ ≤ -45°

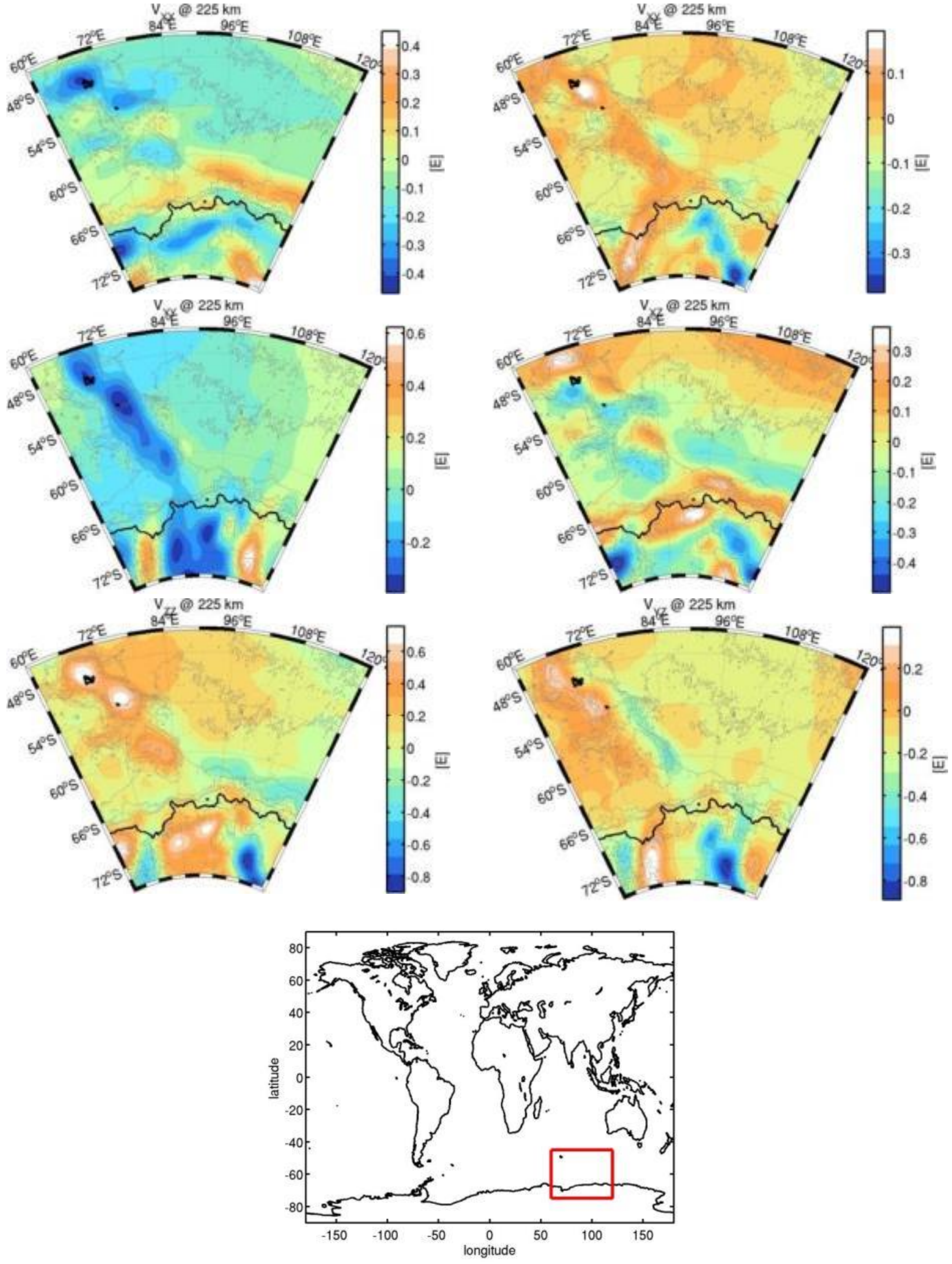

120° ≤ λ ≤ 180°, -75° ≤ φ ≤ -45°

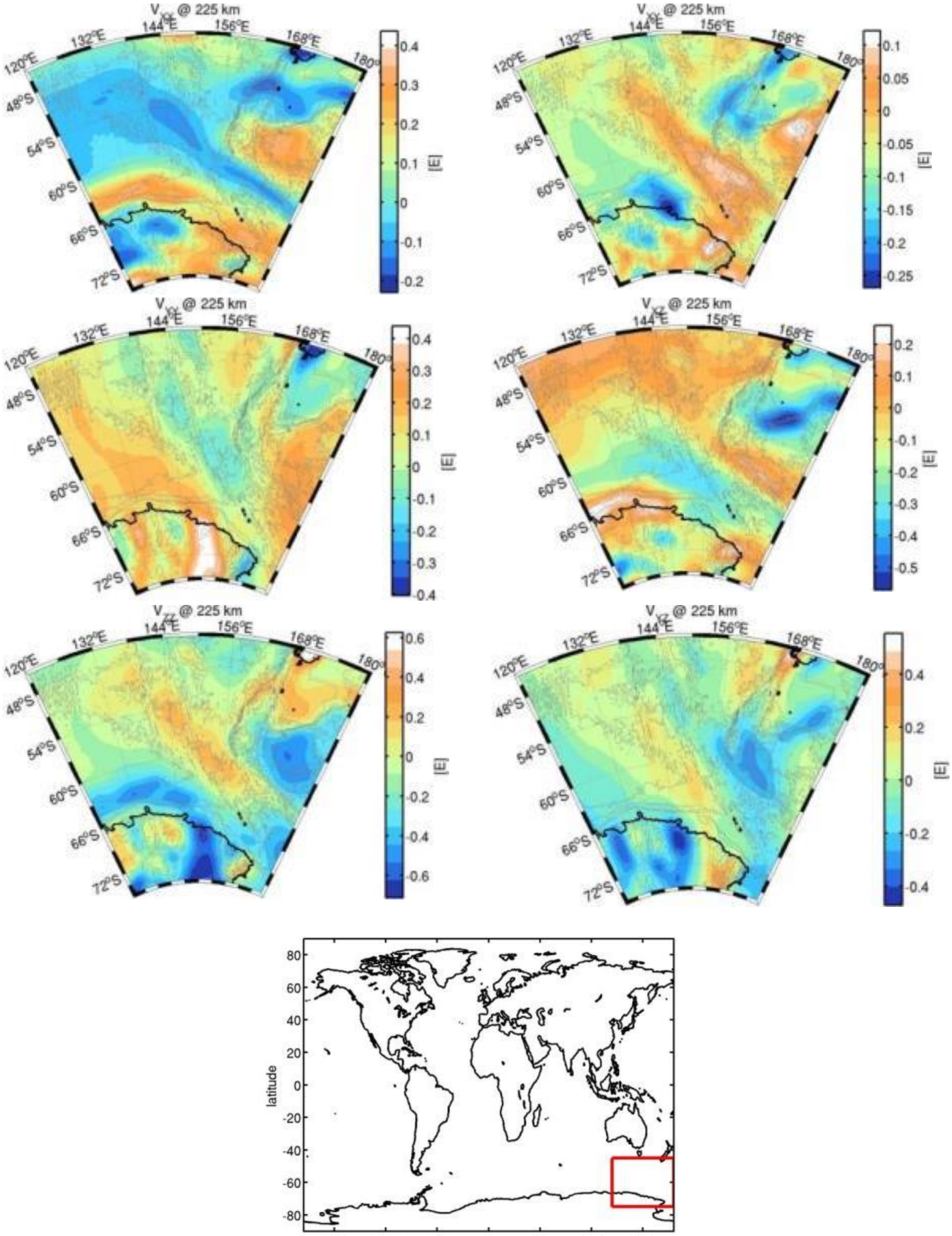

-180° ≤ λ ≤ -120°, -45° ≤ φ ≤ -15°

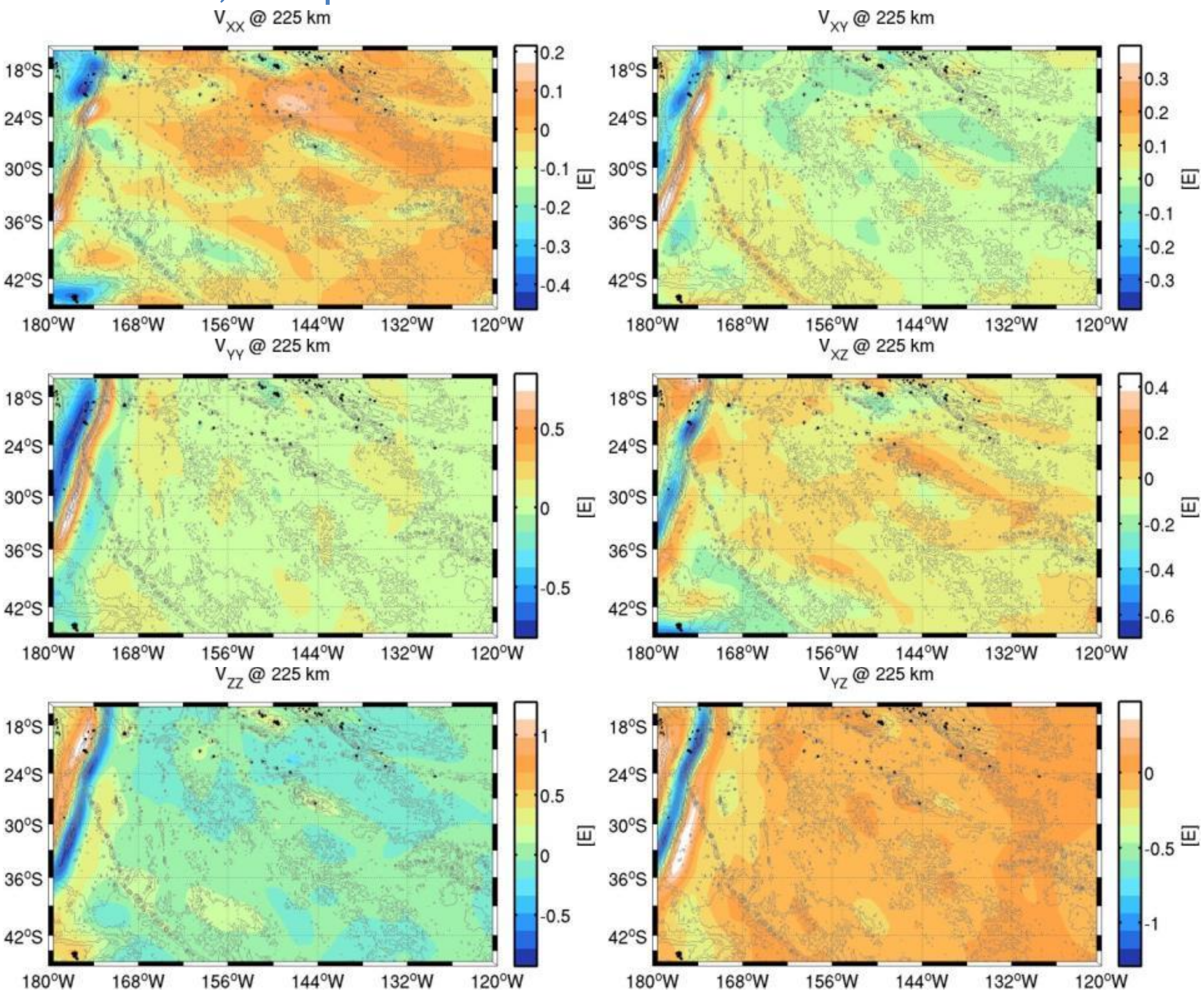

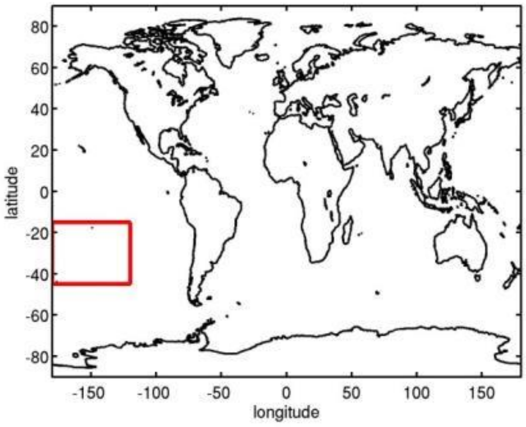

-120° ≤ λ ≤ -60°, -45° ≤ φ ≤ -15°

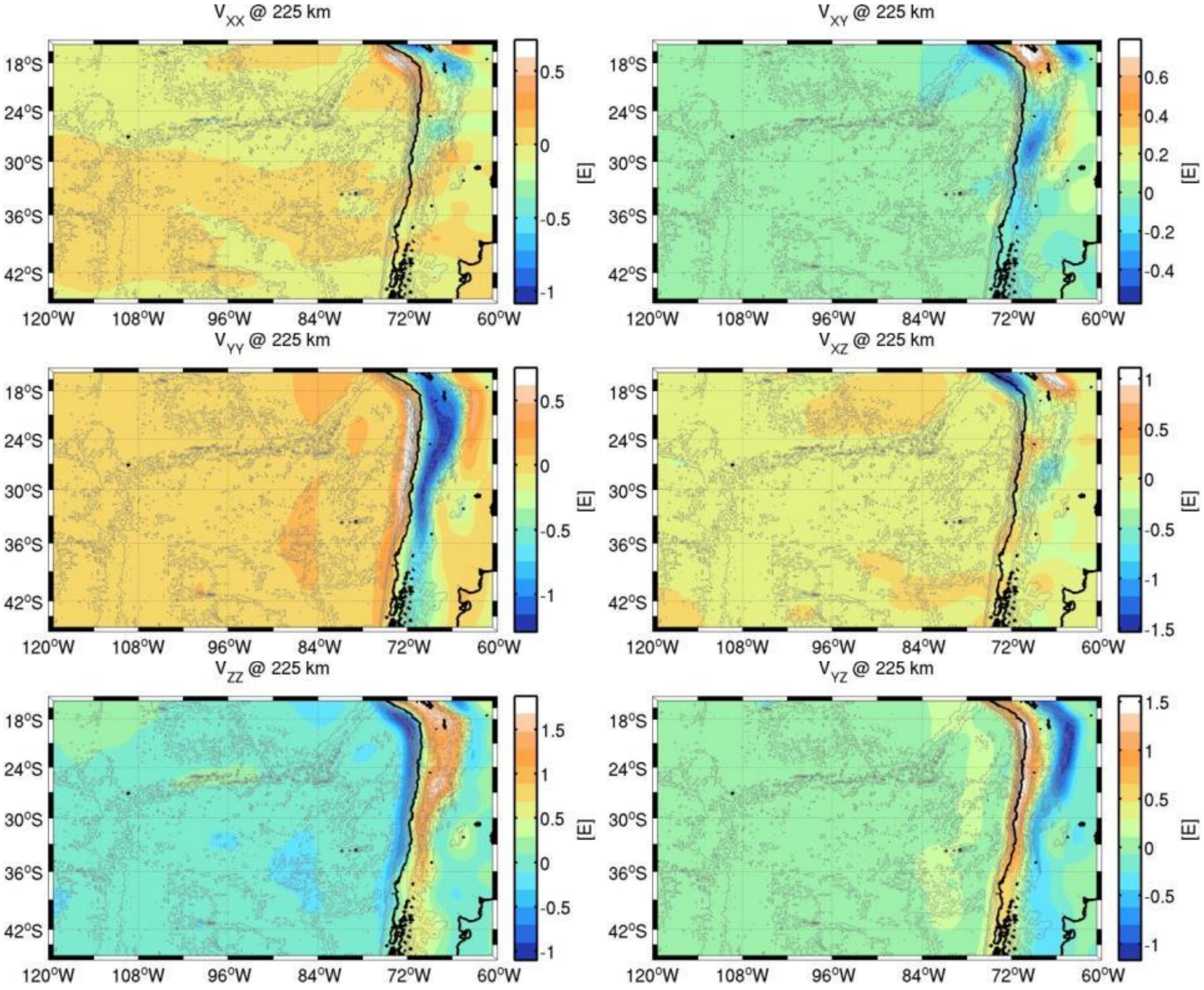

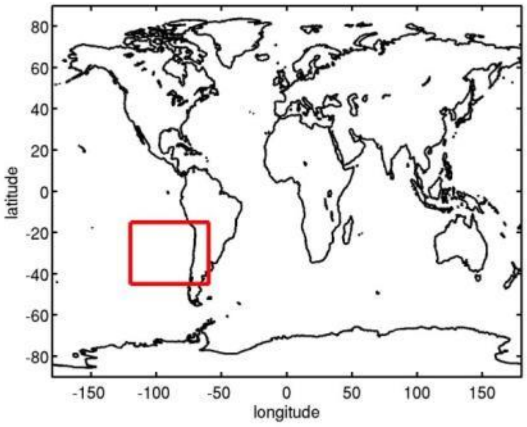

$-60° \le \lambda \le 0°, -45° \le \varphi \le -15°$

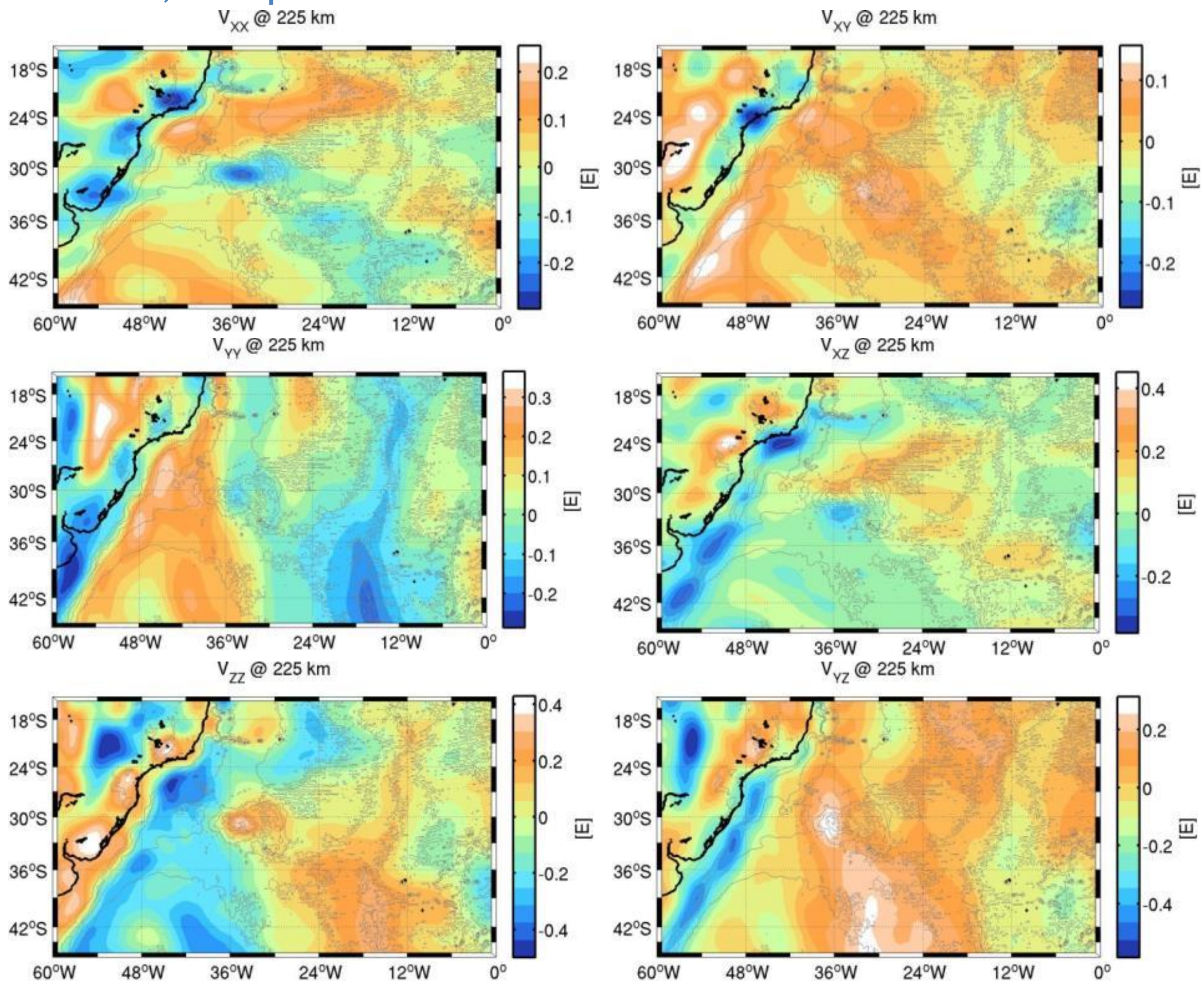

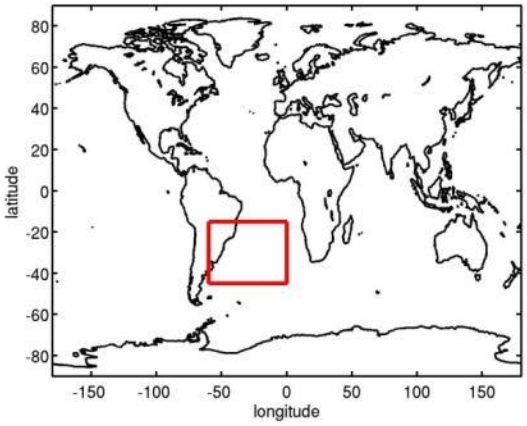

0° ≤ λ ≤ 60°, -45° ≤ φ ≤ -15°

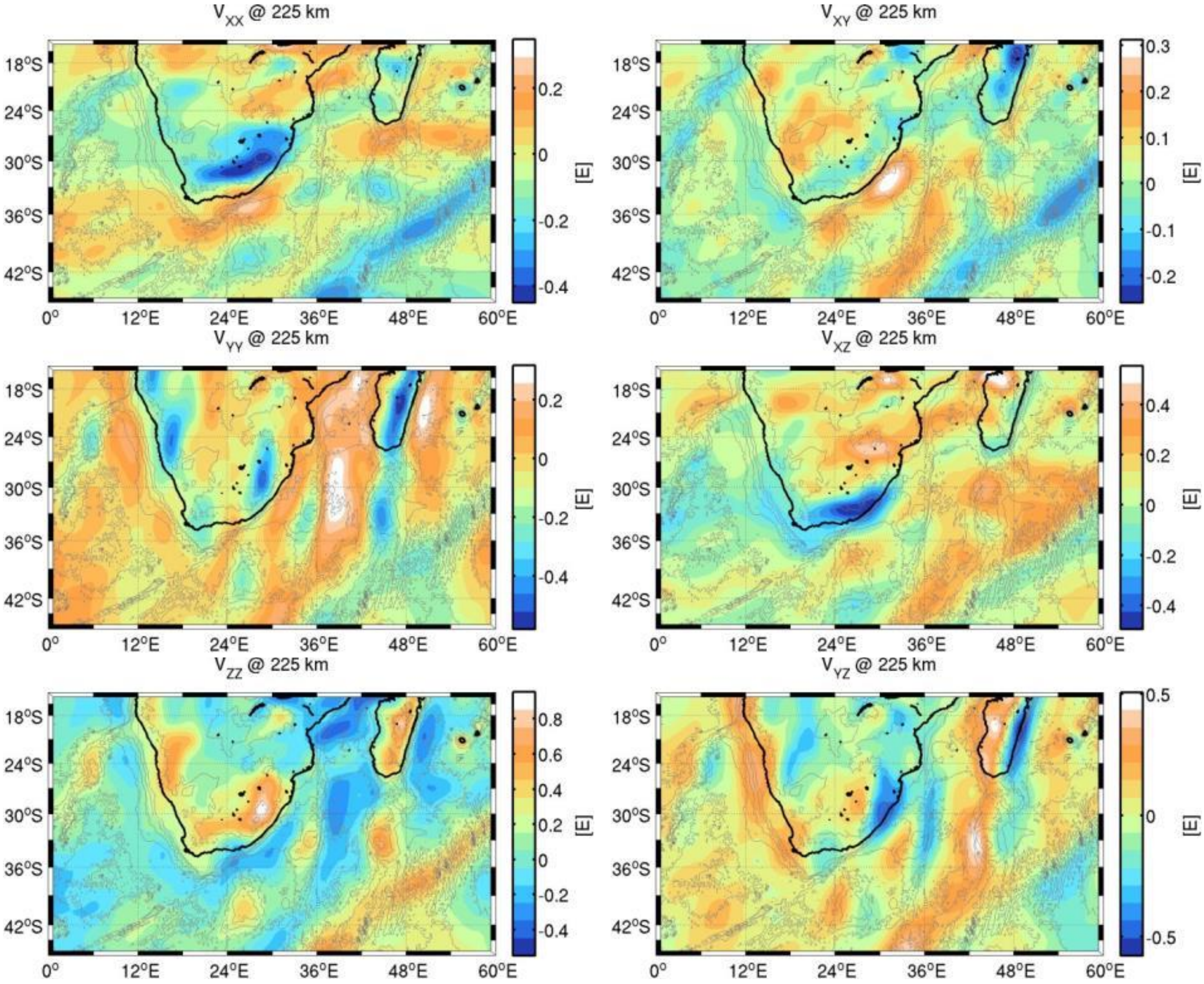

V_XX @ 225 km
V_XY @ 225 km
V_YY @ 225 km
V_XZ @ 225 km
V_ZZ @ 225 km
V_YZ @ 225 km
18°S
24°S
30°S
36°S
42°S
0°
12°E
24°E
36°E
48°E
60°E
[E]

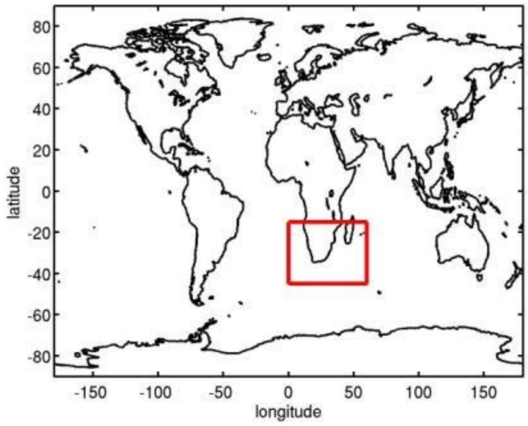

latitude
longitude
80
60
40
20
0
-20
-40
-60
-80
-150
-100
-50
0
50
100
150

60° ≤ λ ≤ -120°, -45° ≤ φ ≤ -15°

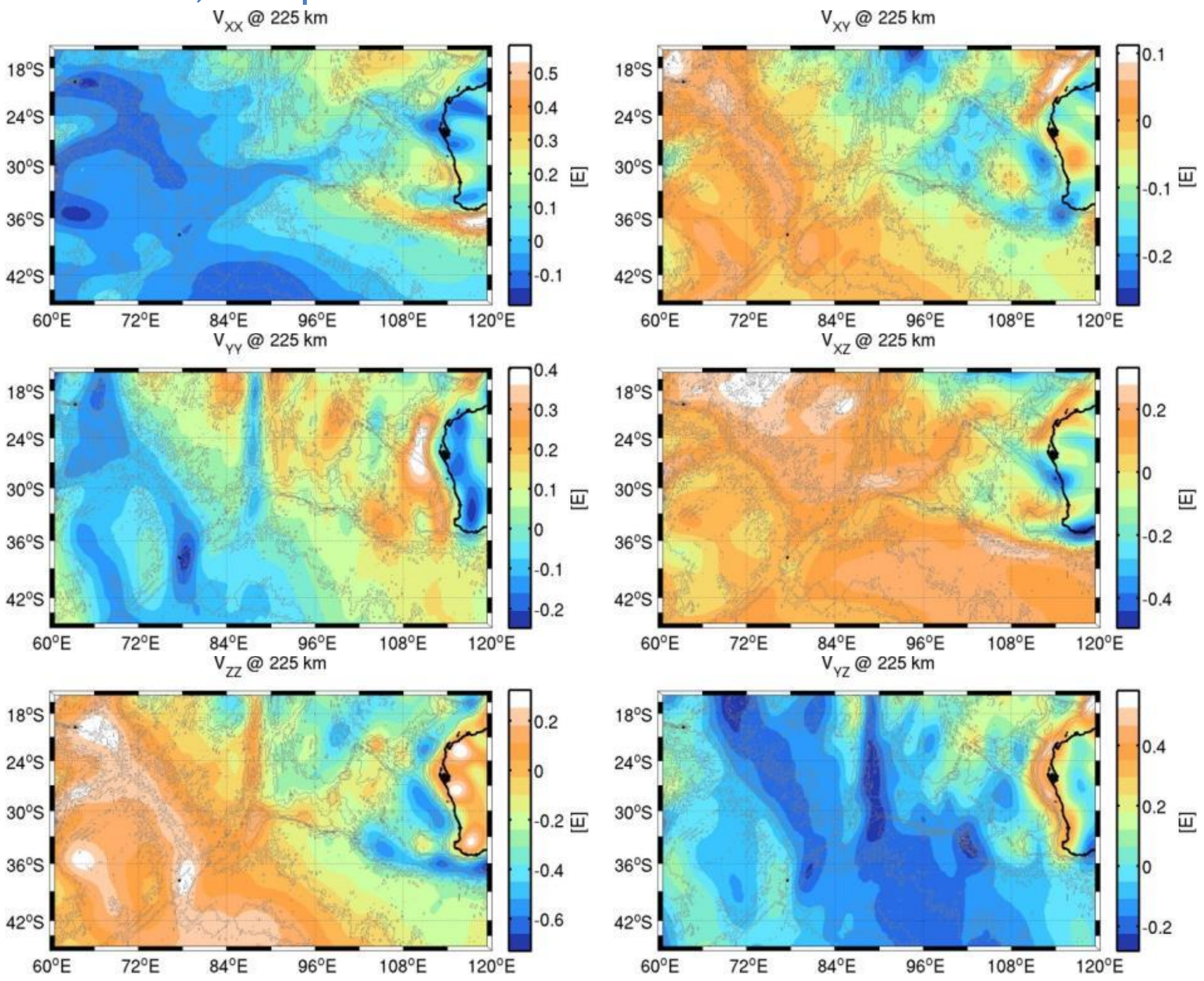

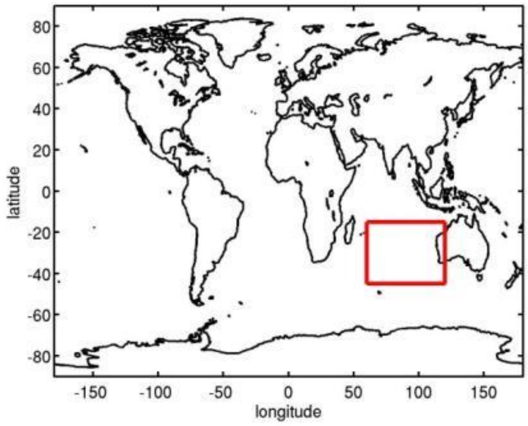

120° ≤ λ ≤ 180°, -45° ≤ φ ≤ -15°

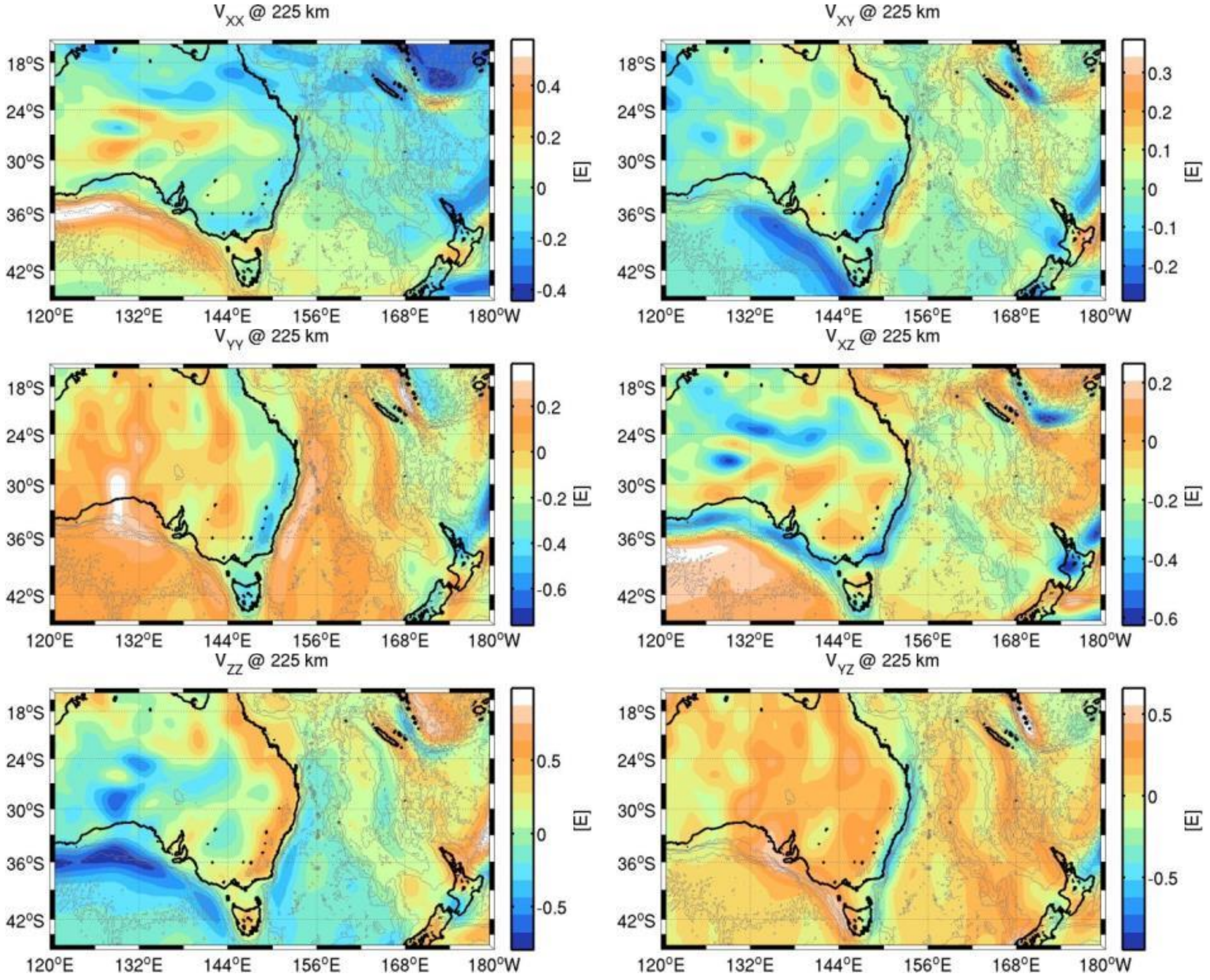

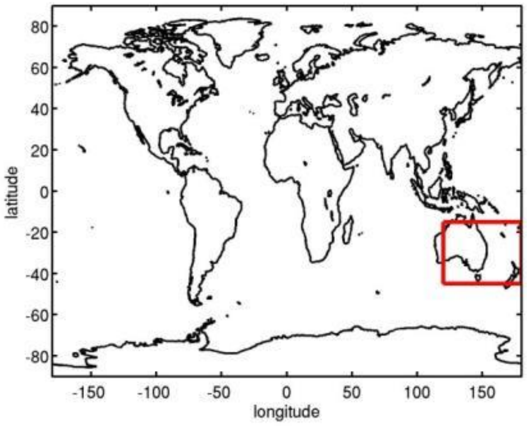

-180° ≤ λ ≤ -120°, -15° ≤ φ ≤ 15°

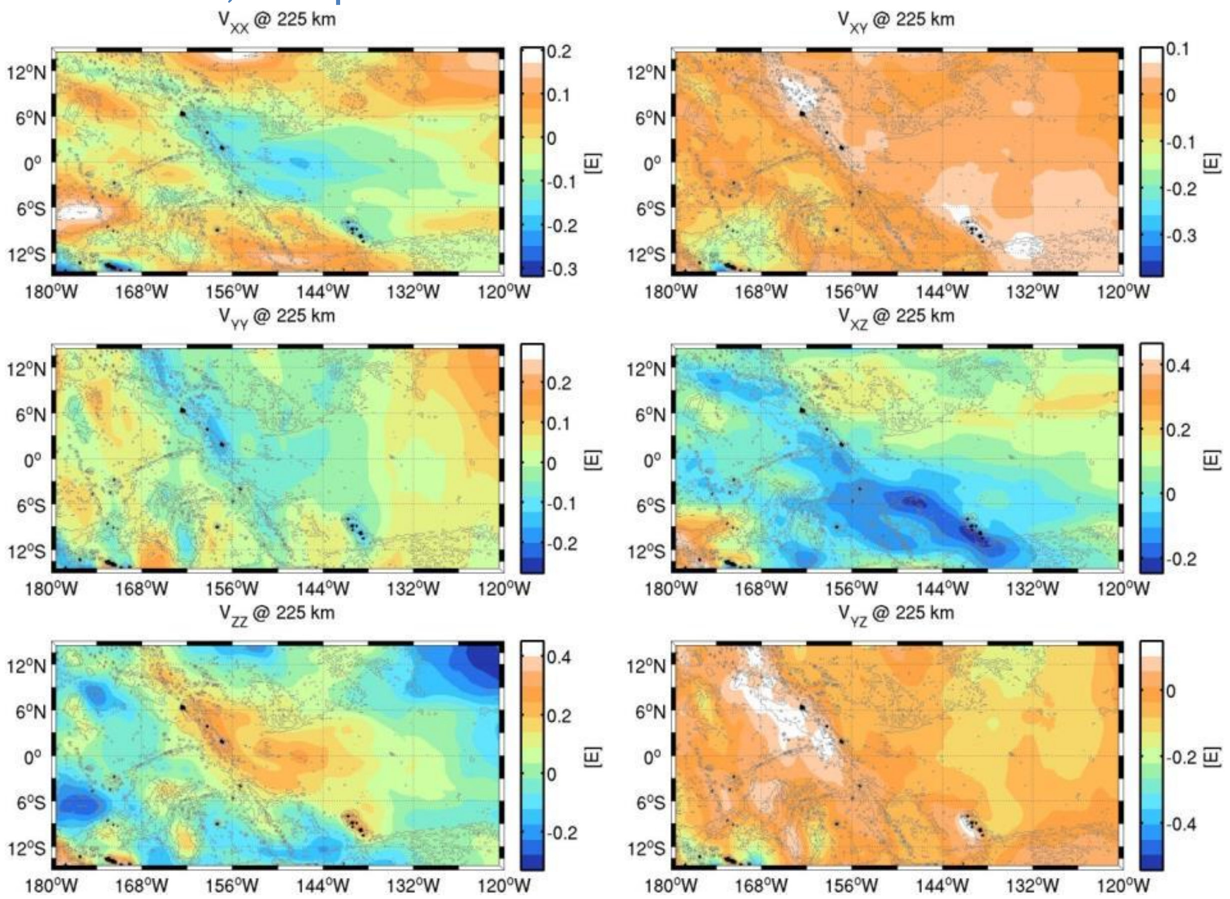

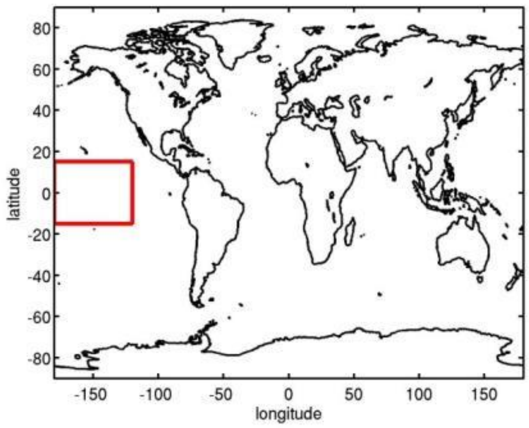

-120° ≤ λ ≤ -60°, -15° ≤ φ ≤ 15°

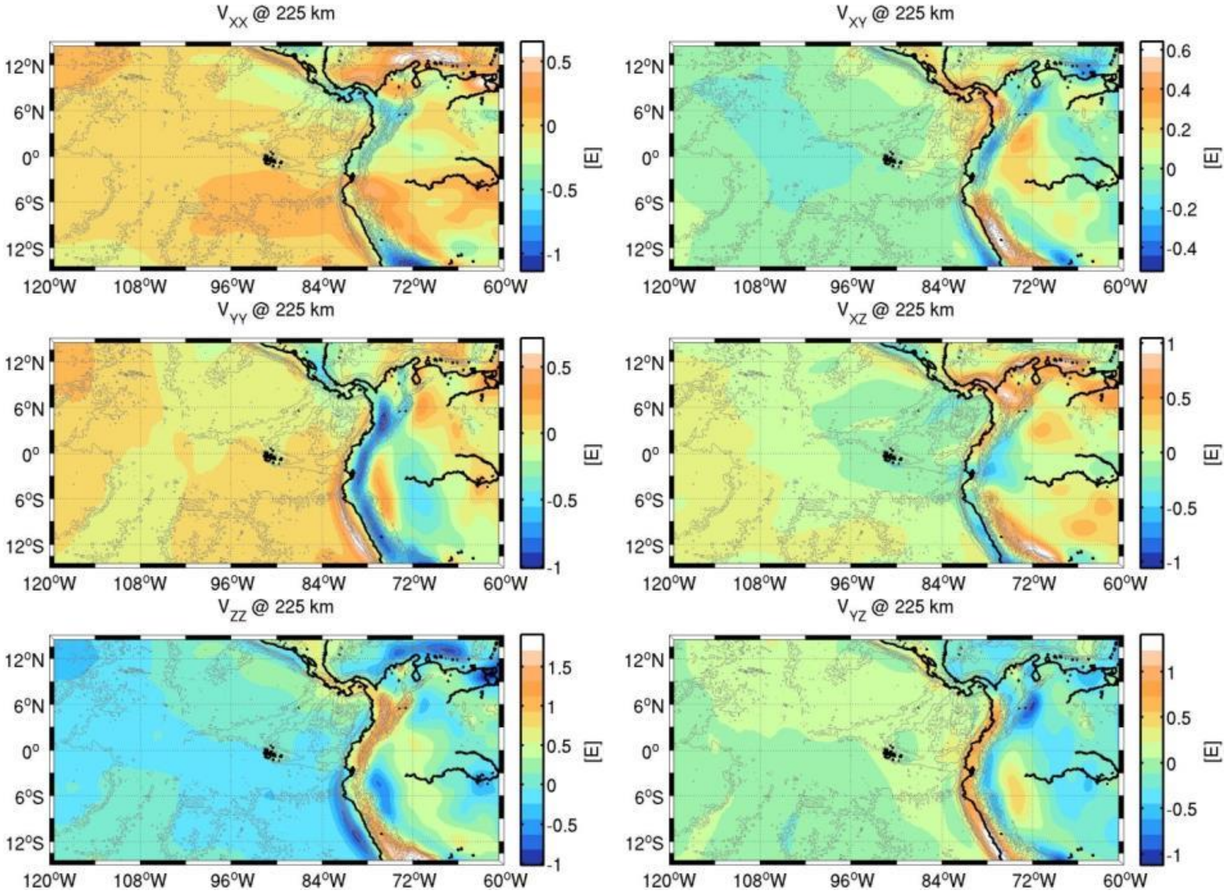

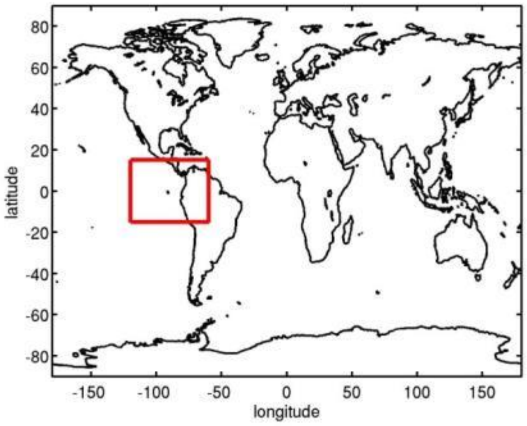

-60° ≤ λ ≤ 0°, -15° ≤ φ ≤ 15°

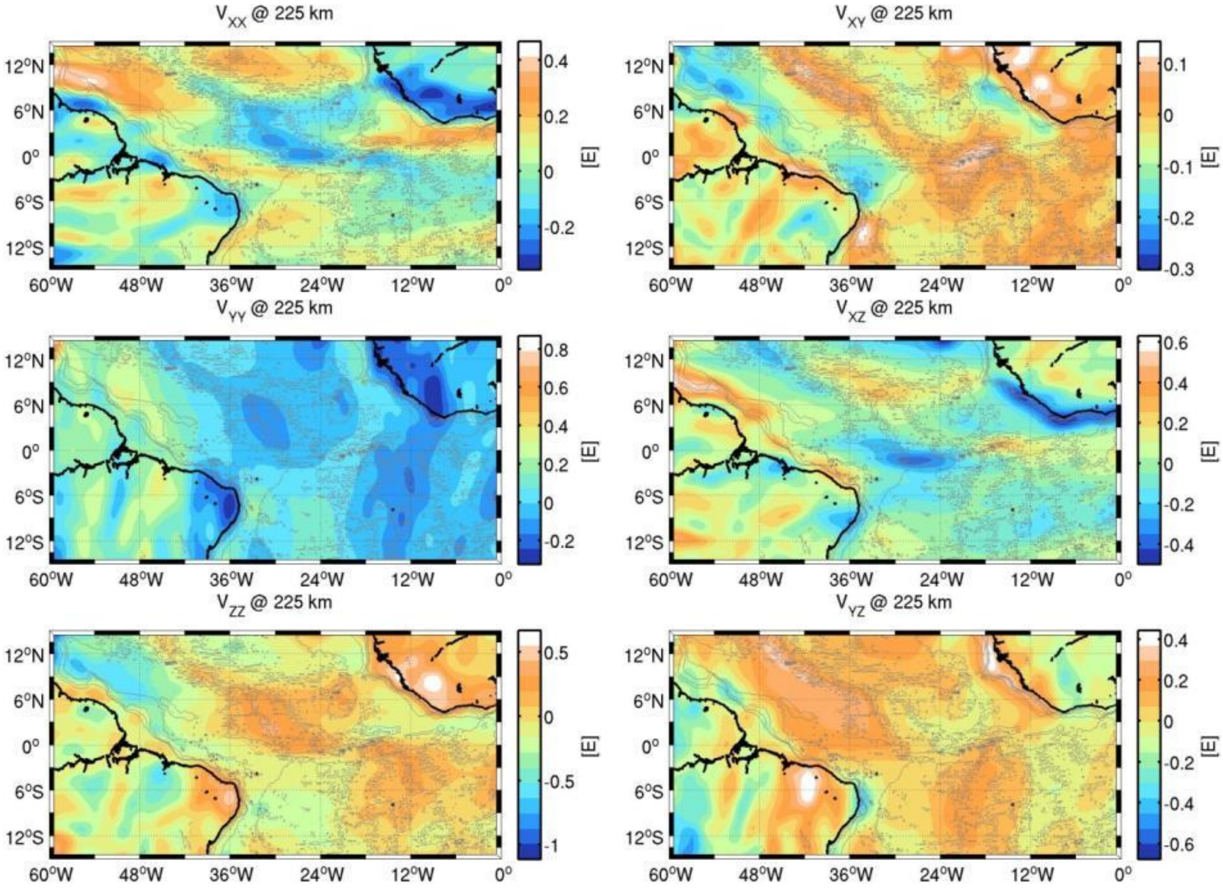

V_{XX} @ 225 km
V_{XY} @ 225 km
V_{YY} @ 225 km
V_{XZ} @ 225 km
V_{ZZ} @ 225 km
V_{YZ} @ 225 km
12°N
6°N
0°
6°S
12°S
60°W
48°W
36°W
24°W
12°W
0°
[E]

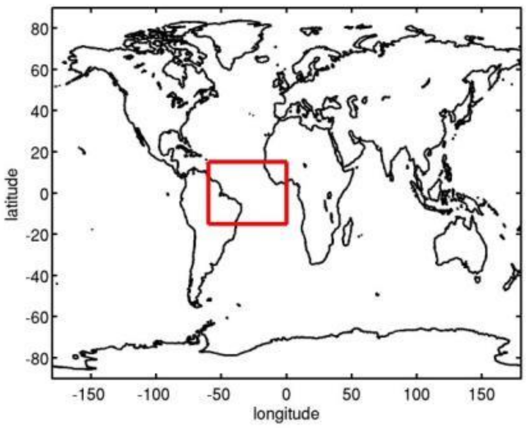

latitude
longitude

0° ≤ λ ≤ 60°, -15° ≤ φ ≤ 15°

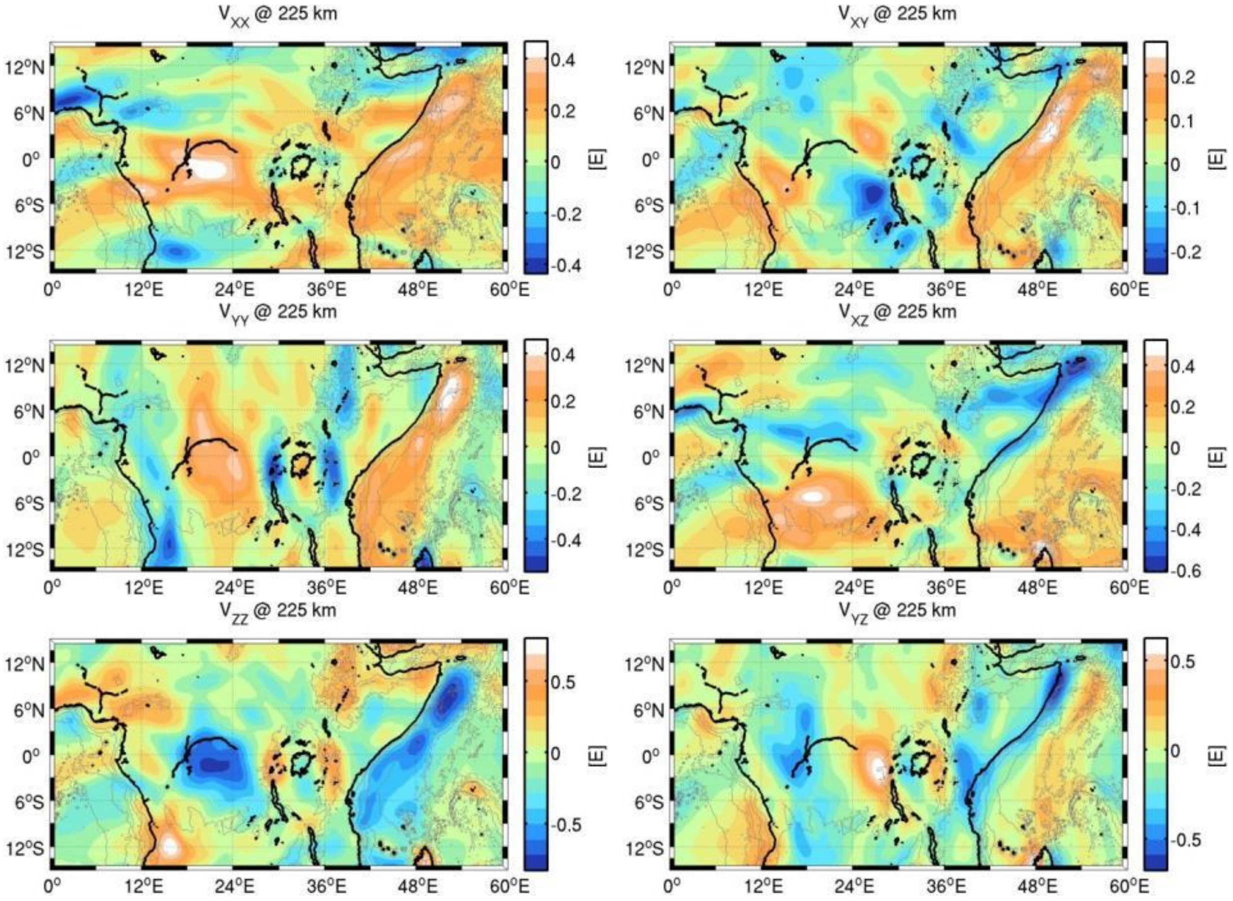

V_{XX} @ 225 km
V_{XY} @ 225 km
V_{YY} @ 225 km
V_{XZ} @ 225 km
V_{ZZ} @ 225 km
V_{YZ} @ 225 km
12°N
6°N
0°
6°S
12°S
0°
12°E
24°E
36°E
48°E
60°E
[E]

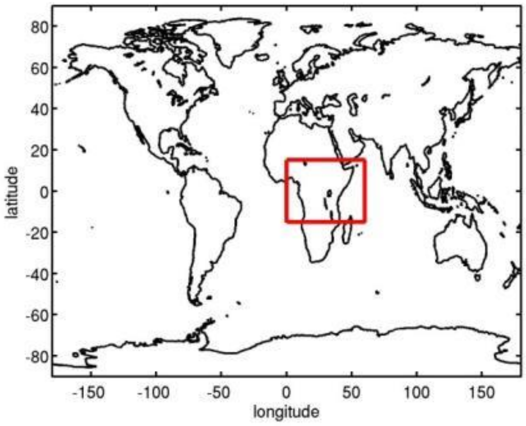

latitude
longitude
80
60
40
20
0
-20
-40
-60
-80
-150
-100
-50
0
50
100
150

$60° \leq \lambda \leq 120°, -15° \leq \varphi \leq 15°$

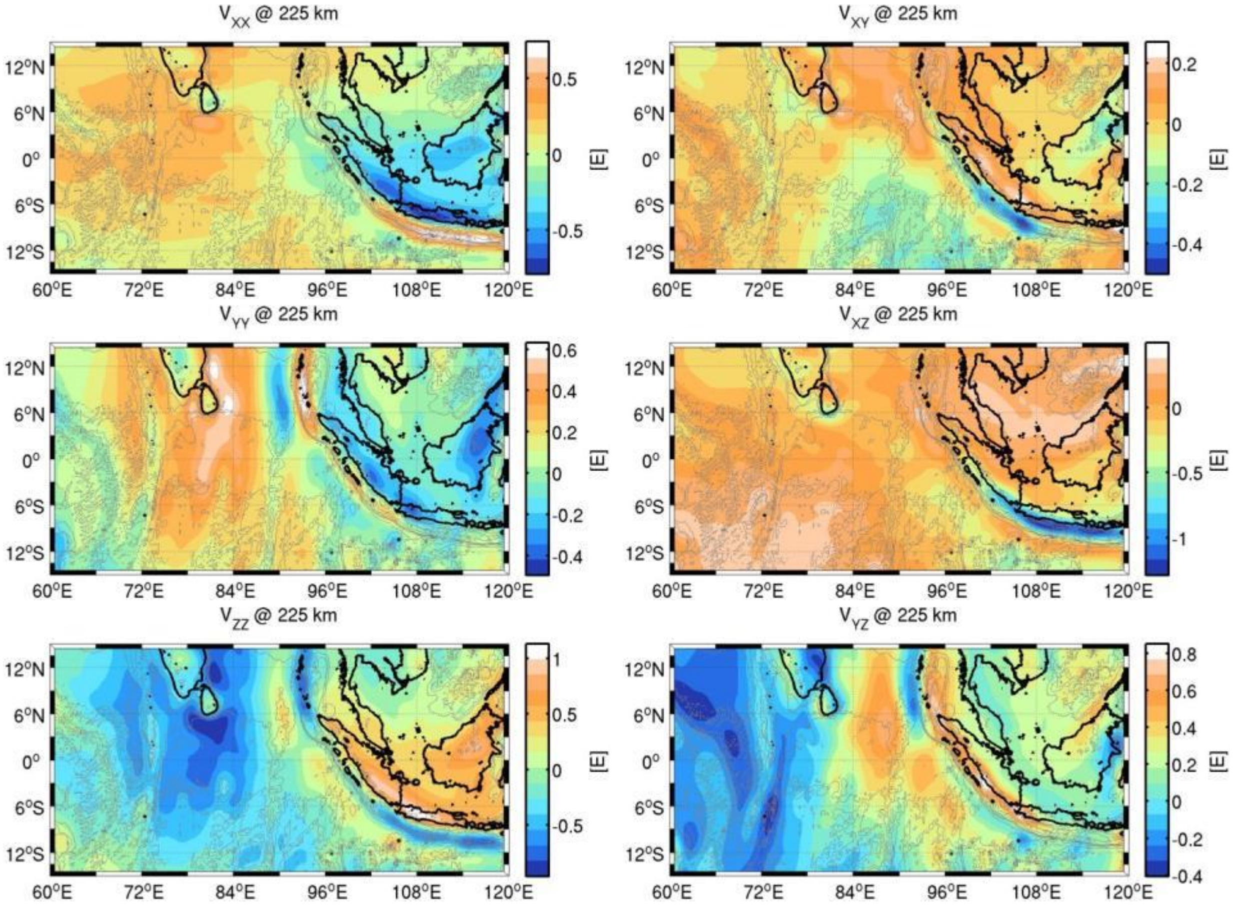

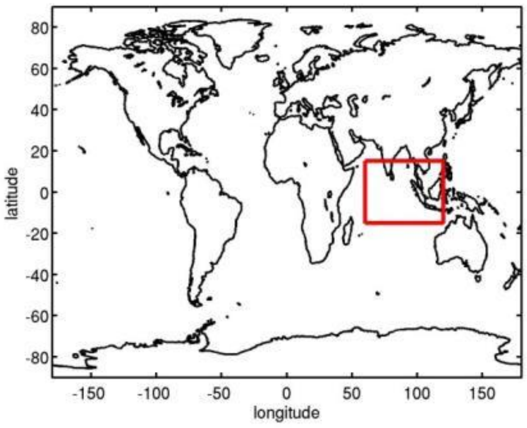

120° ≤ λ ≤ 180°, -15° ≤ φ ≤ 15°

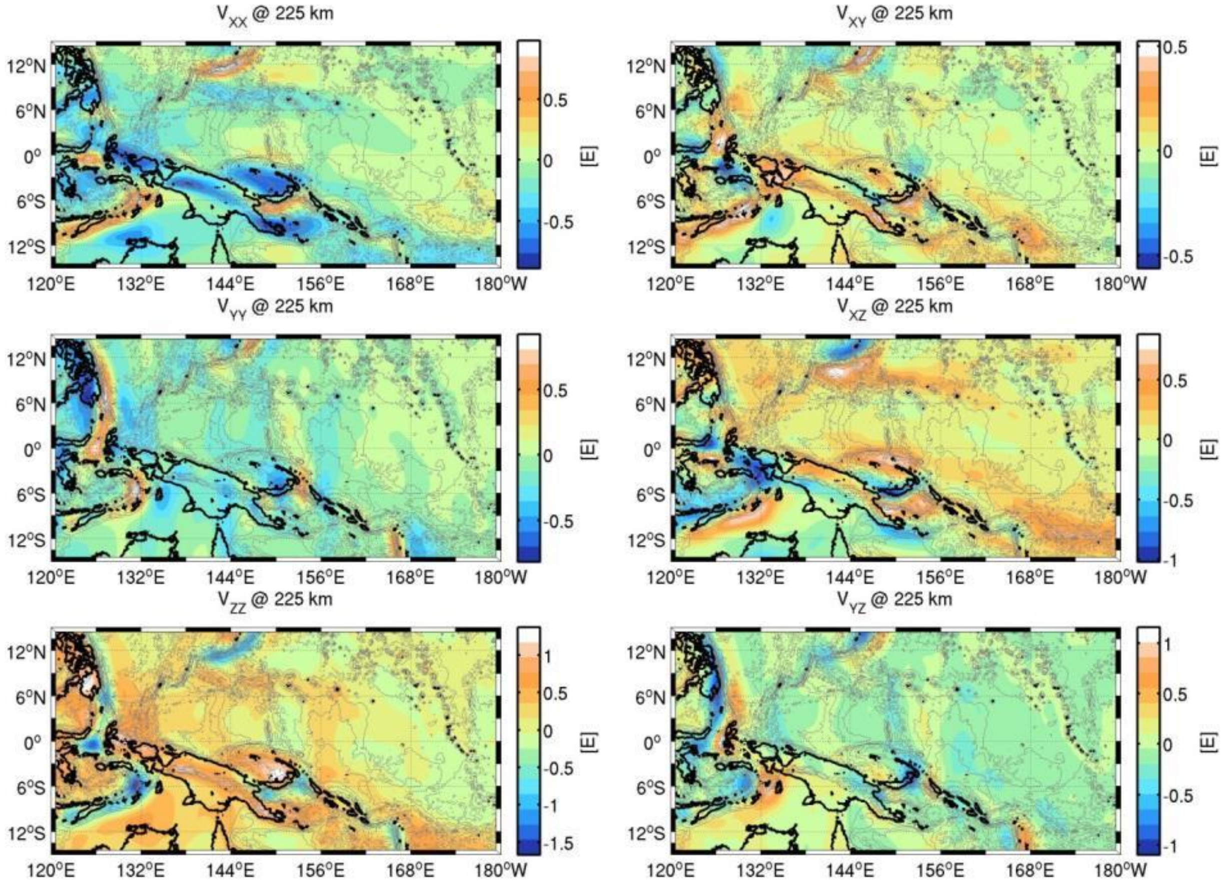

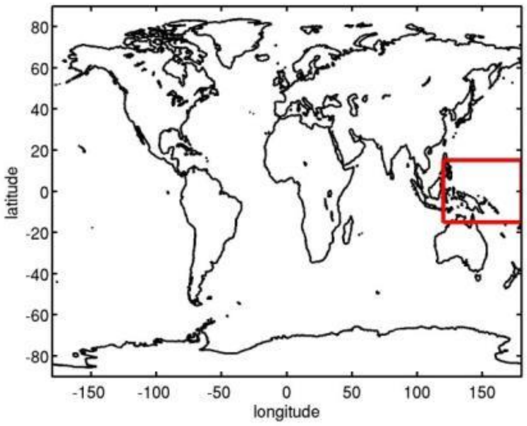

-180° ≤ λ ≤ -120°, 15° ≤ φ ≤ 45°

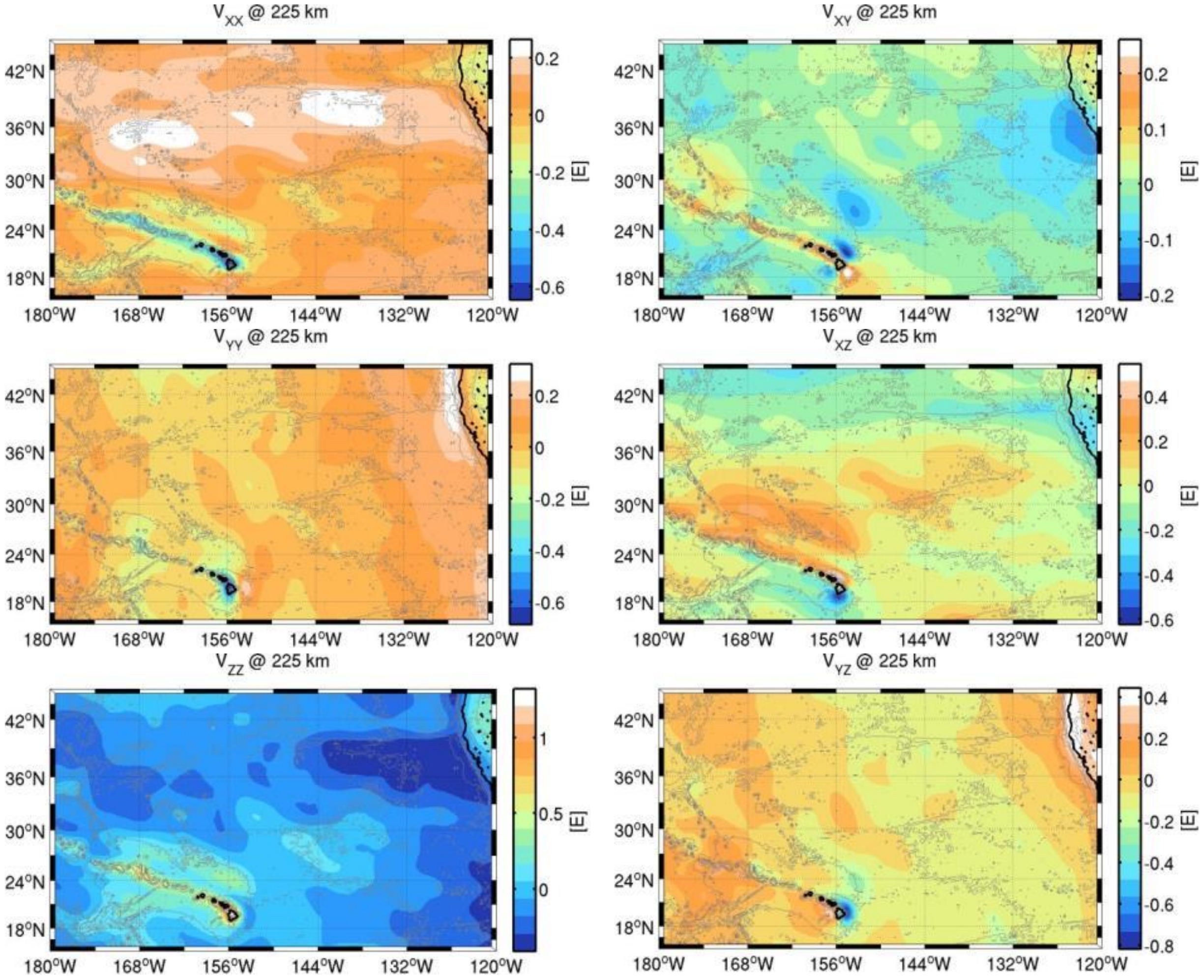

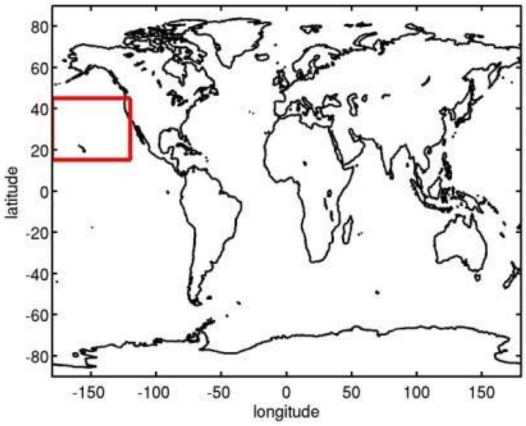

$-120^\circ \le \lambda \le -60^\circ$, $15^\circ \le \varphi \le 45^\circ$

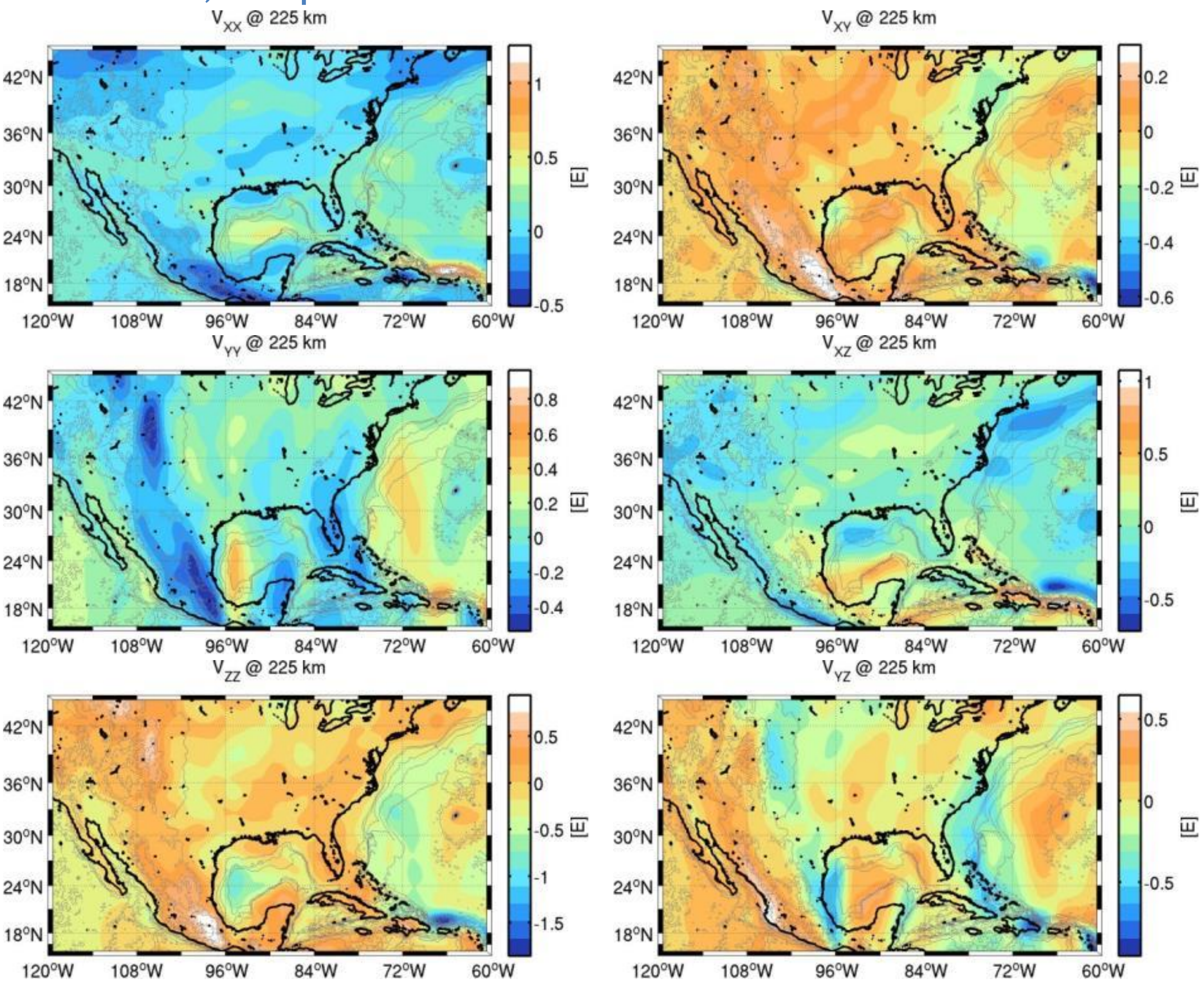

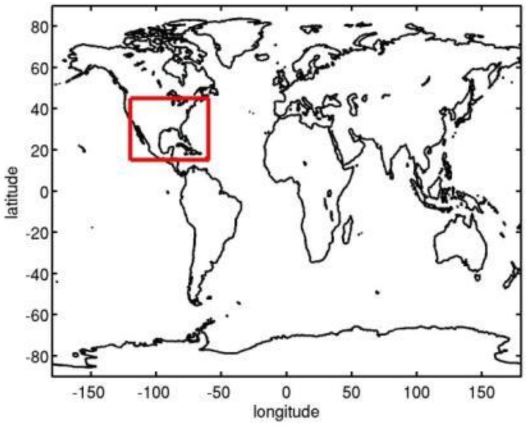

$-180° \leq \lambda \leq -120°$, $15° \leq \varphi \leq 45°$

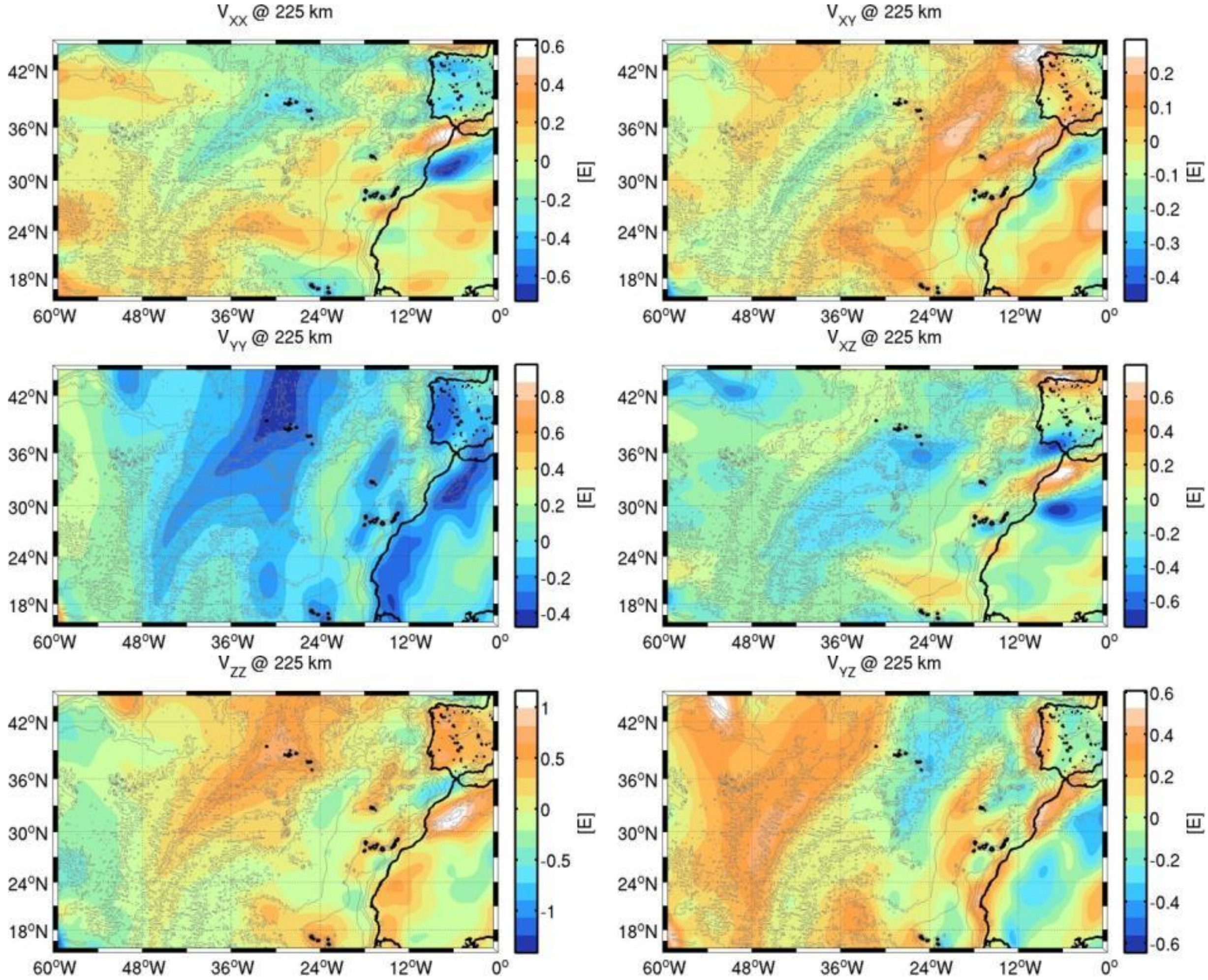

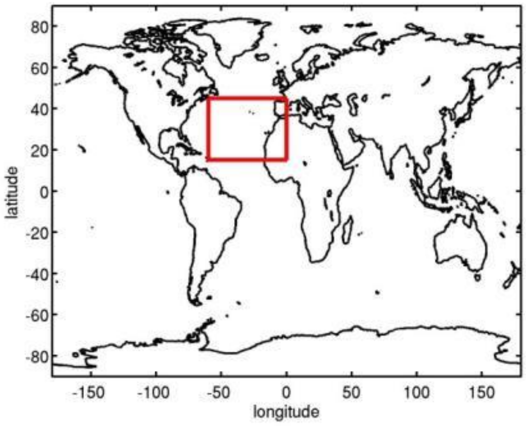

$0° \le \lambda \le 60°$, $15° \le \varphi \le 45°$

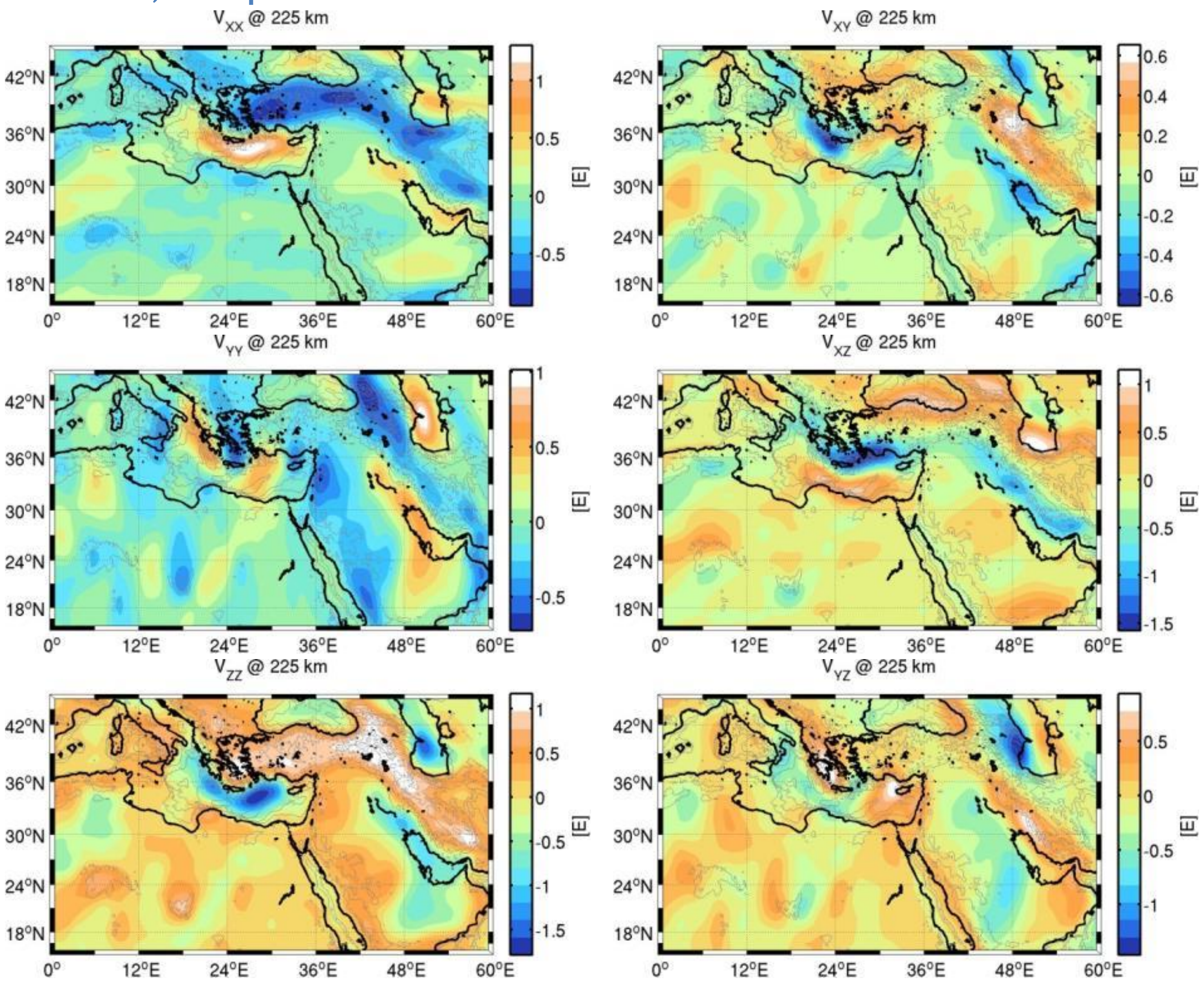

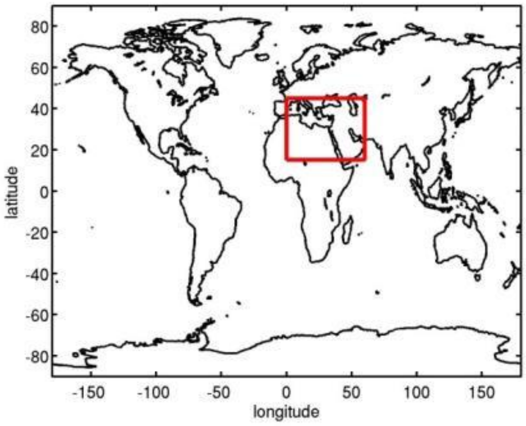

$60° \leq \lambda \leq 120°, 15° \leq \varphi \leq 45°$

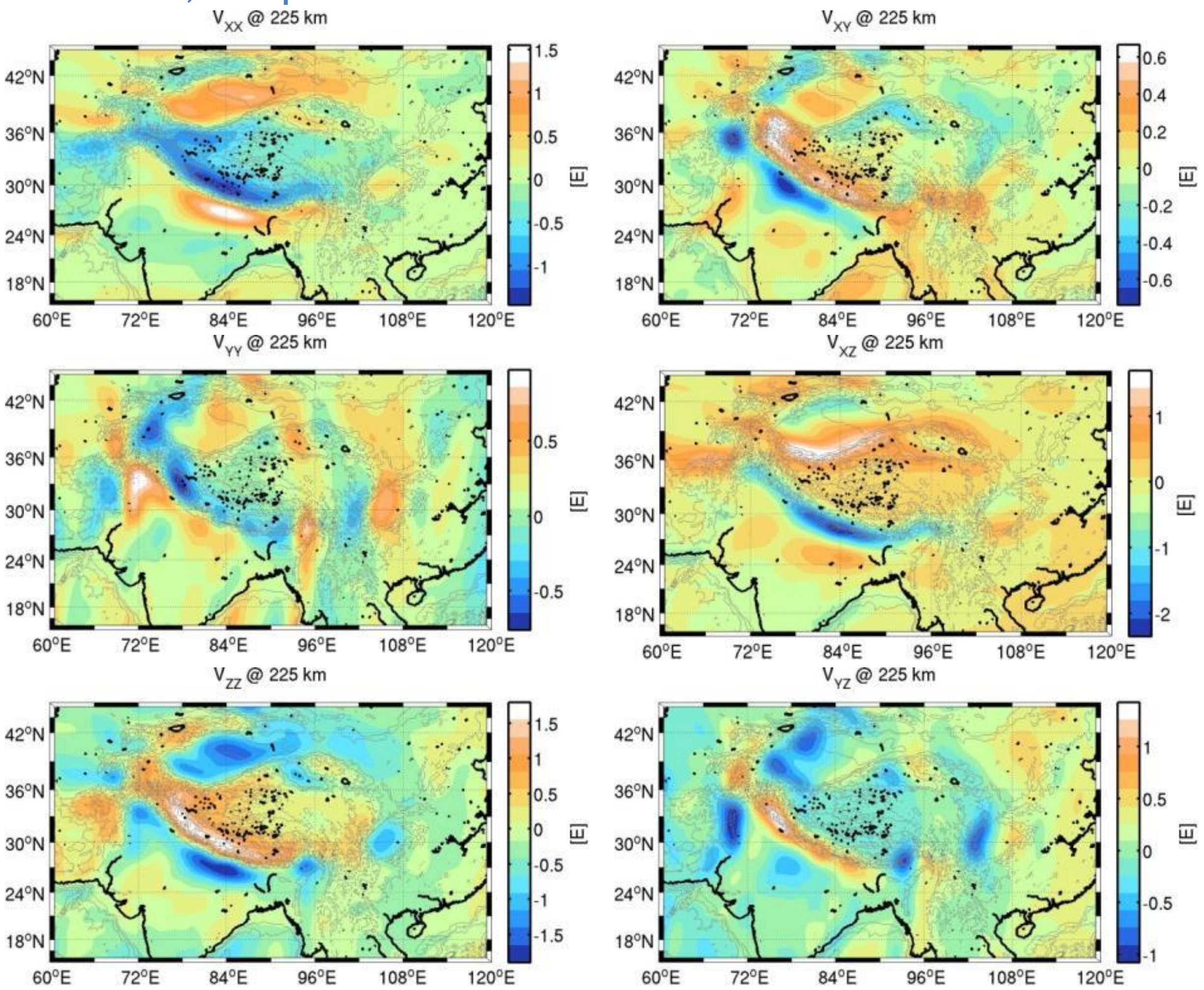

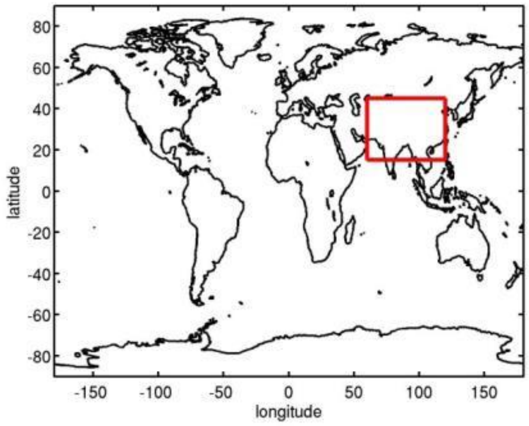

$120° \leq \lambda \leq 180°, 15° \leq \varphi \leq 45°$

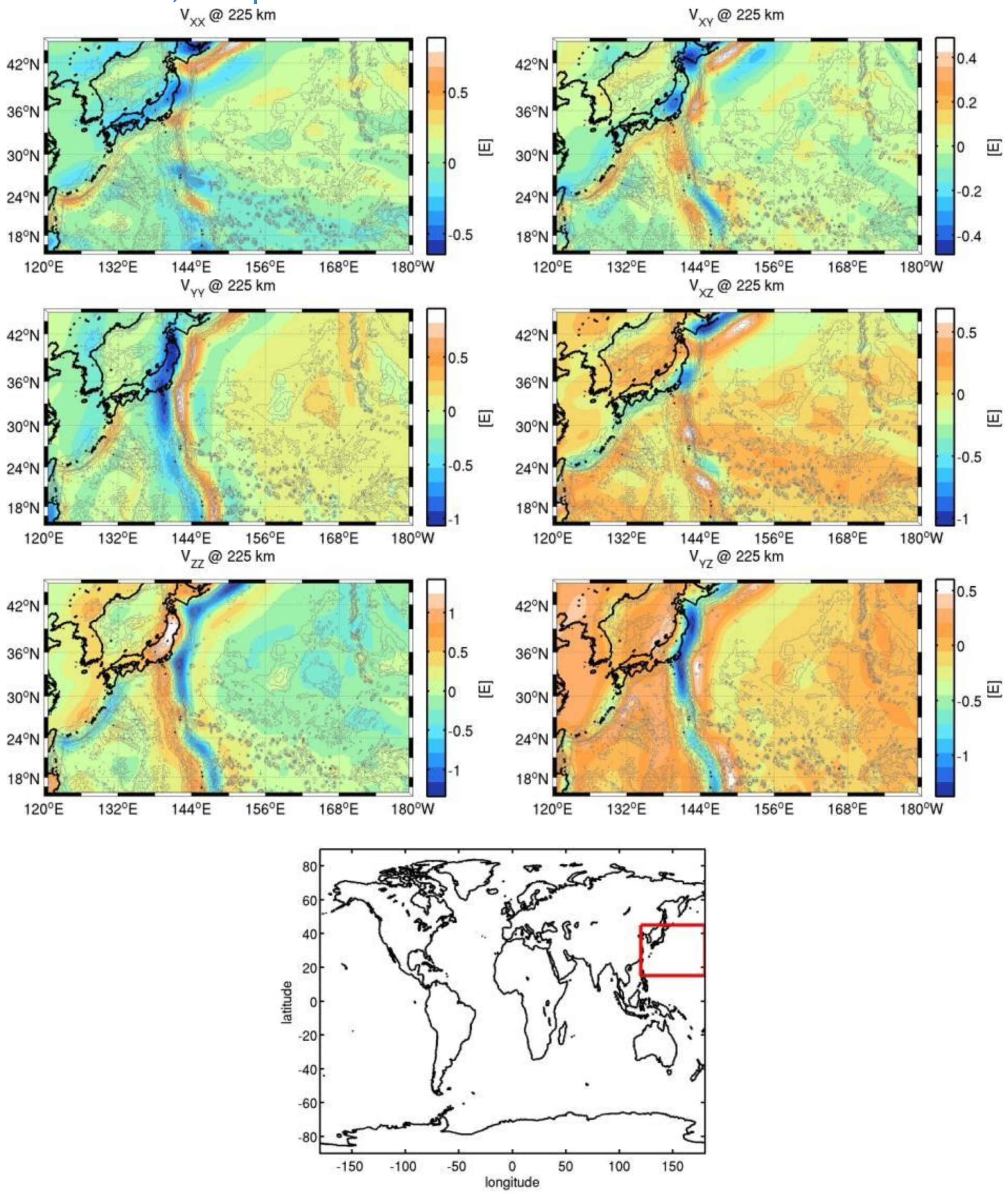

-180° ≤ λ ≤ -120°, 45° ≤ φ ≤ 75°

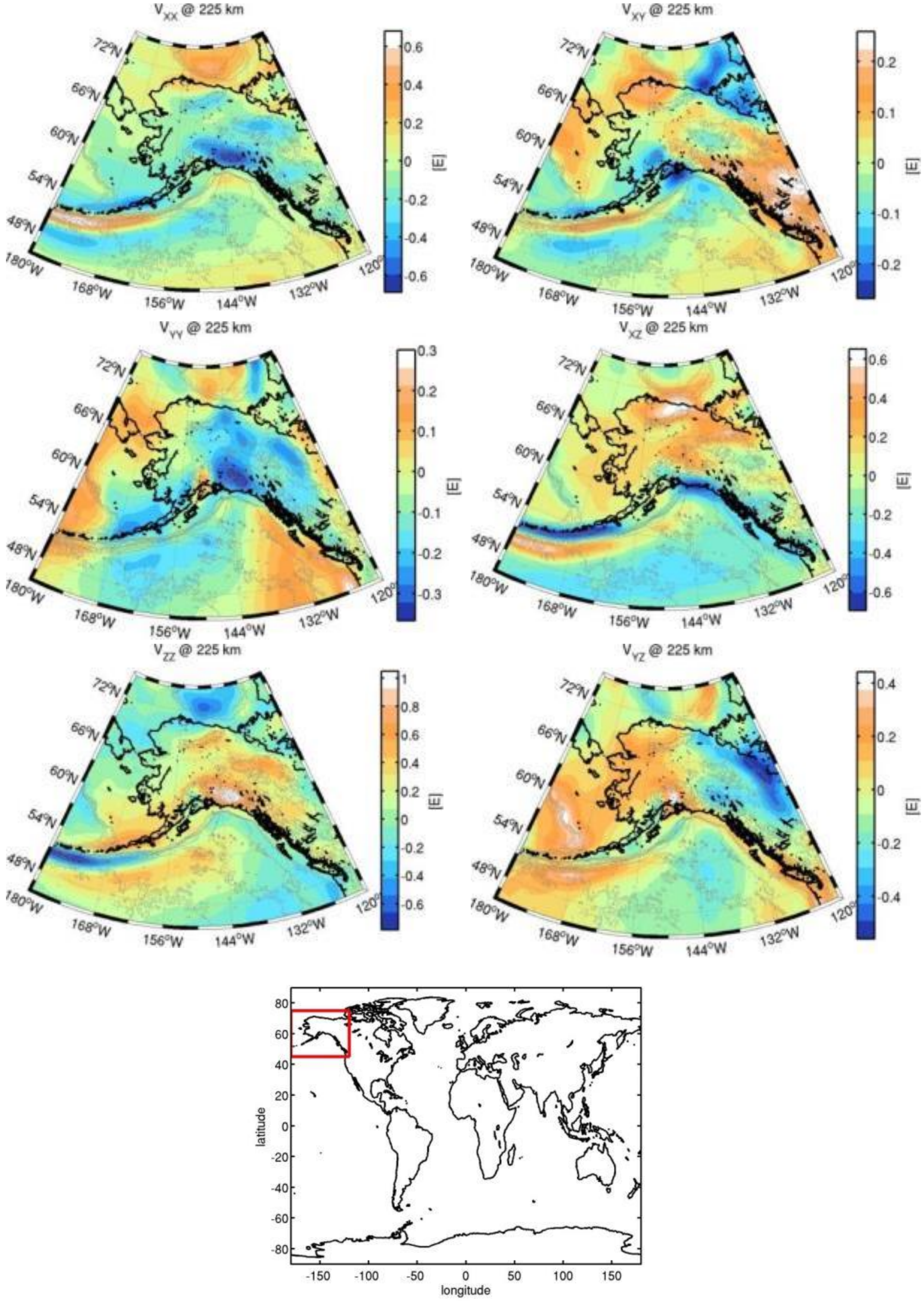

-120° ≤ λ ≤ -60°, 45° ≤ φ ≤ 75°

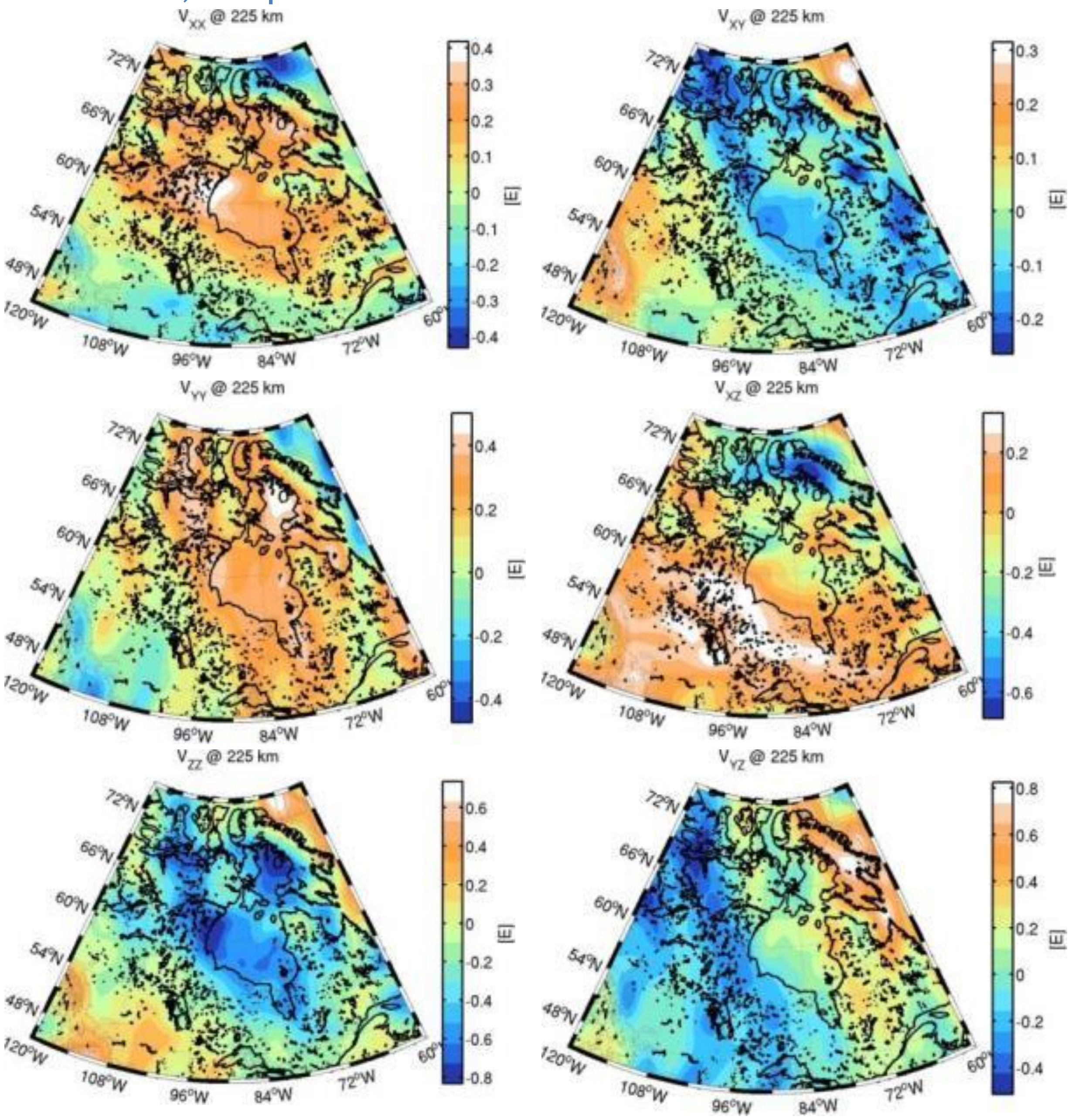

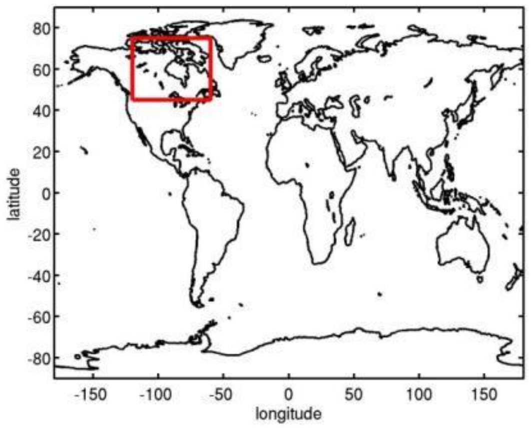

-60° ≤ λ ≤ 0°, 45° ≤ φ ≤ 75°

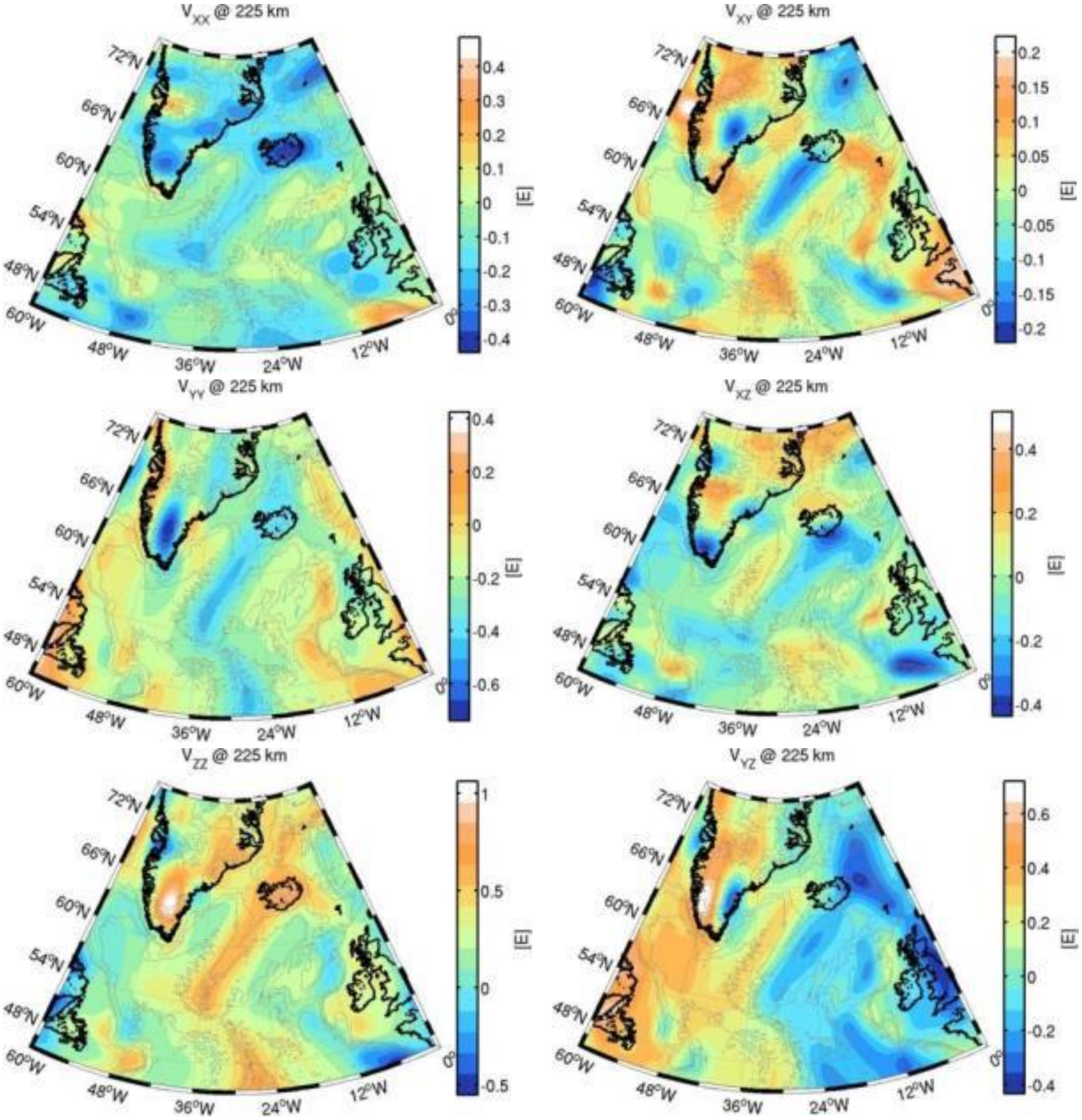

V_XX @ 225 km
V_XY @ 225 km
V_YY @ 225 km
V_XZ @ 225 km
V_ZZ @ 225 km
V_YZ @ 225 km
[E]
72°N
66°N
60°N
54°N
48°N
60°W
48°W
36°W
24°W
12°W
0°

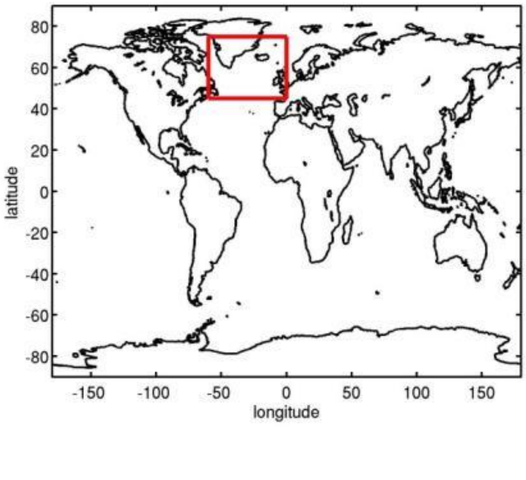

latitude
longitude

$0° \leq \lambda \leq 60°, 45° \leq \varphi \leq 75°$

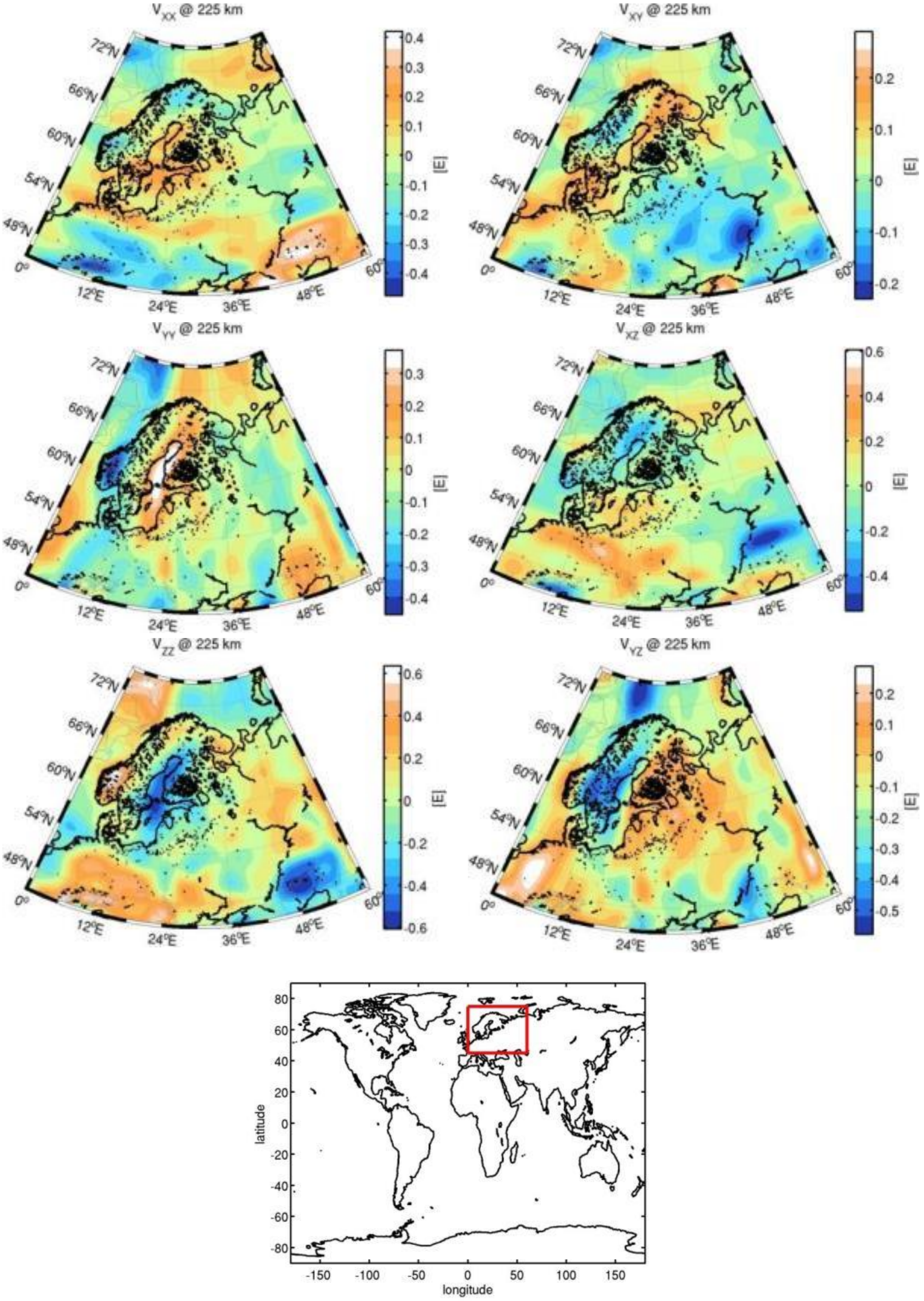

$60° \leq \lambda \leq 120°, 45° \leq \varphi \leq 75°$

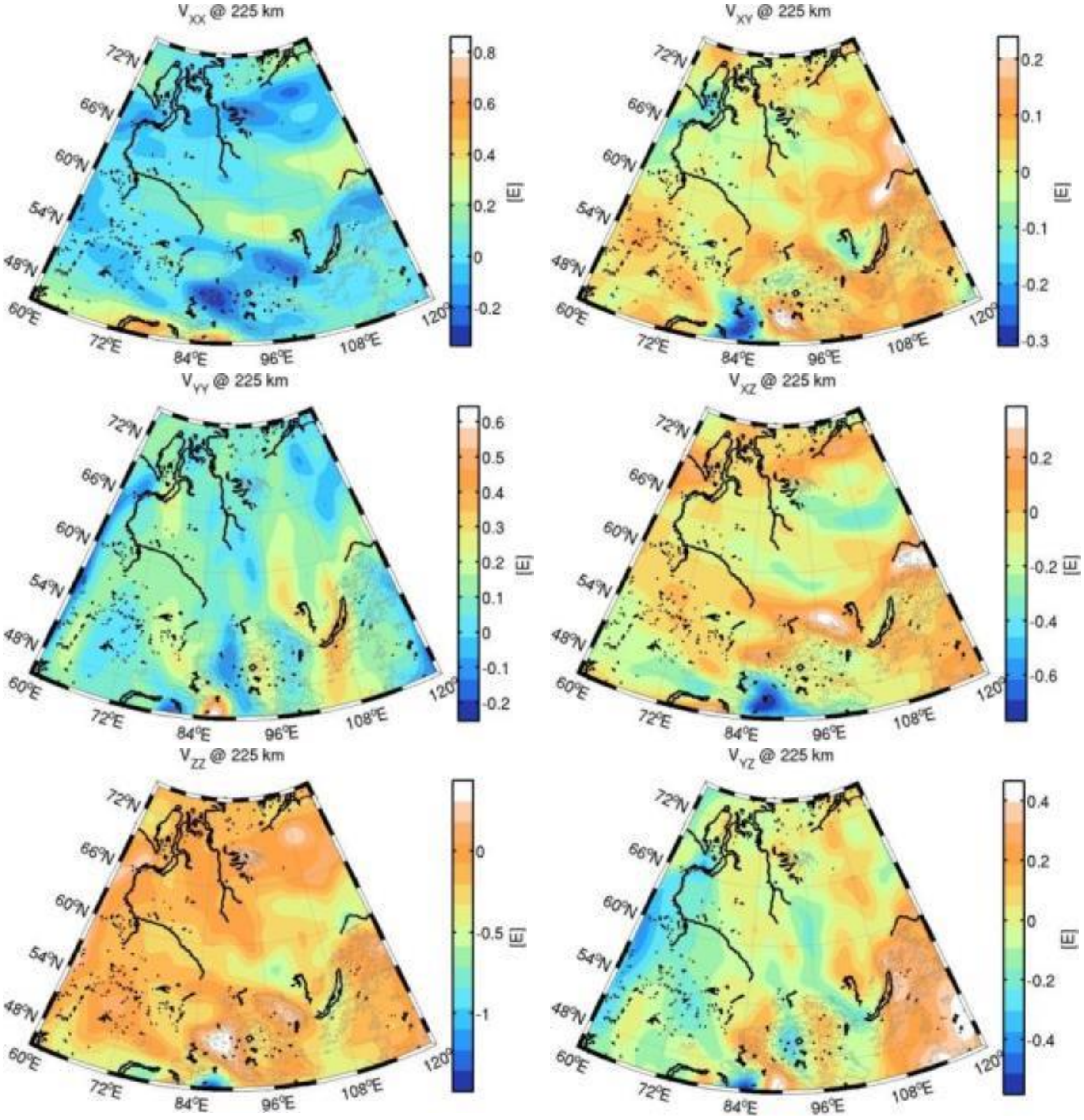

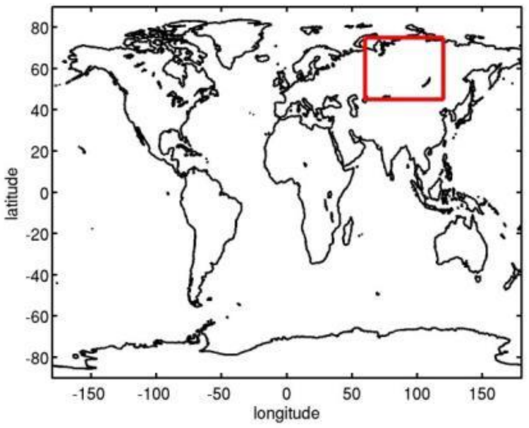

120° ≤ λ ≤ 180°, 45° ≤ φ ≤ 75°

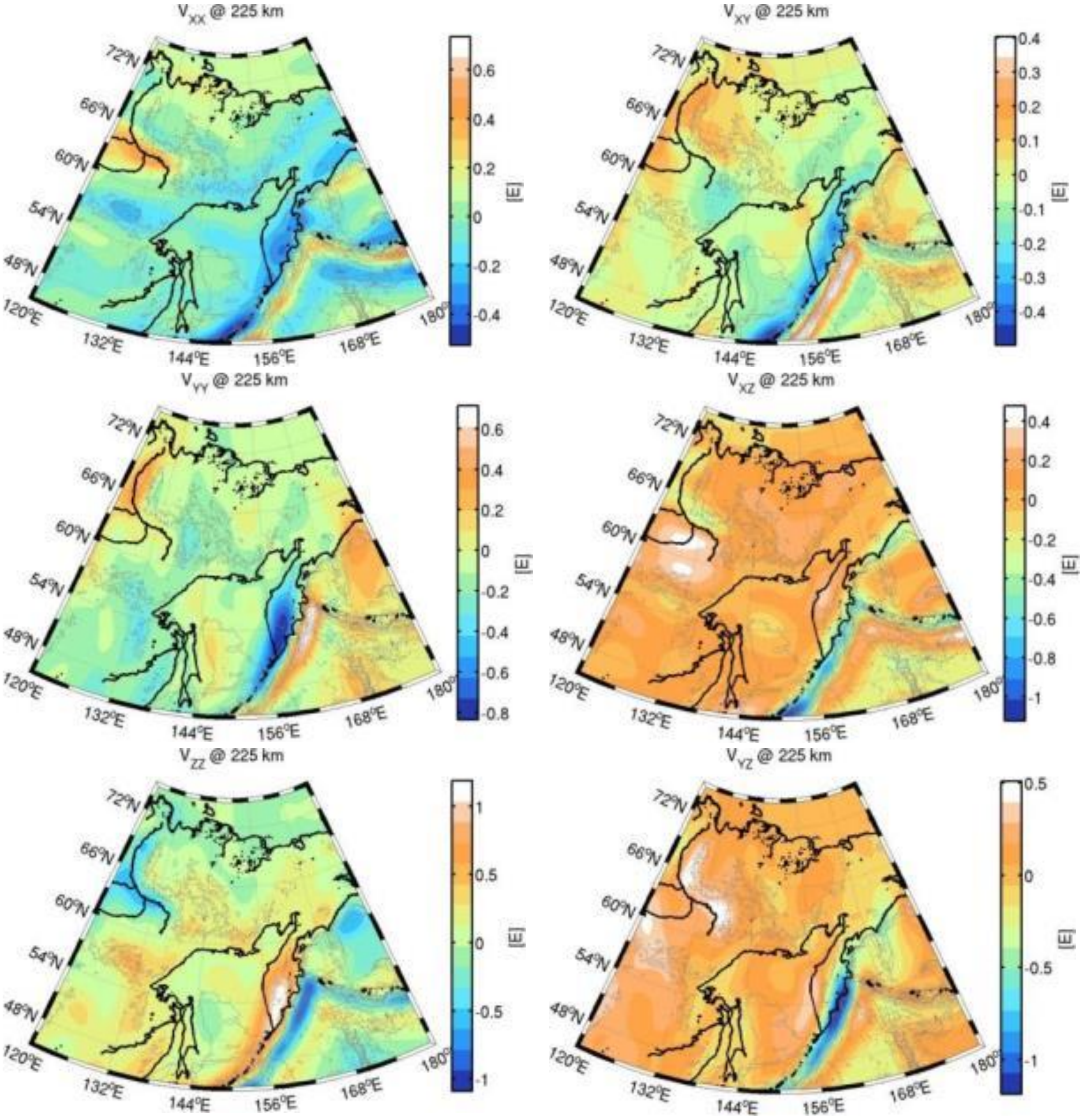

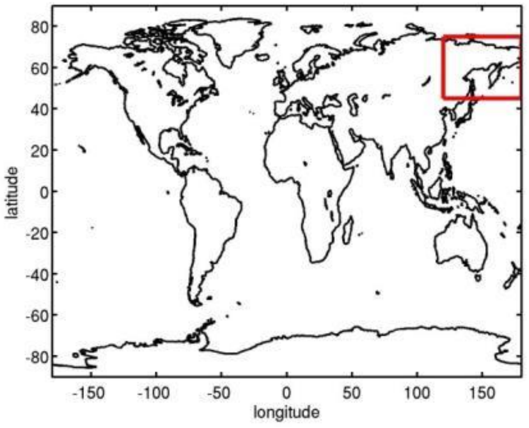

North Pole

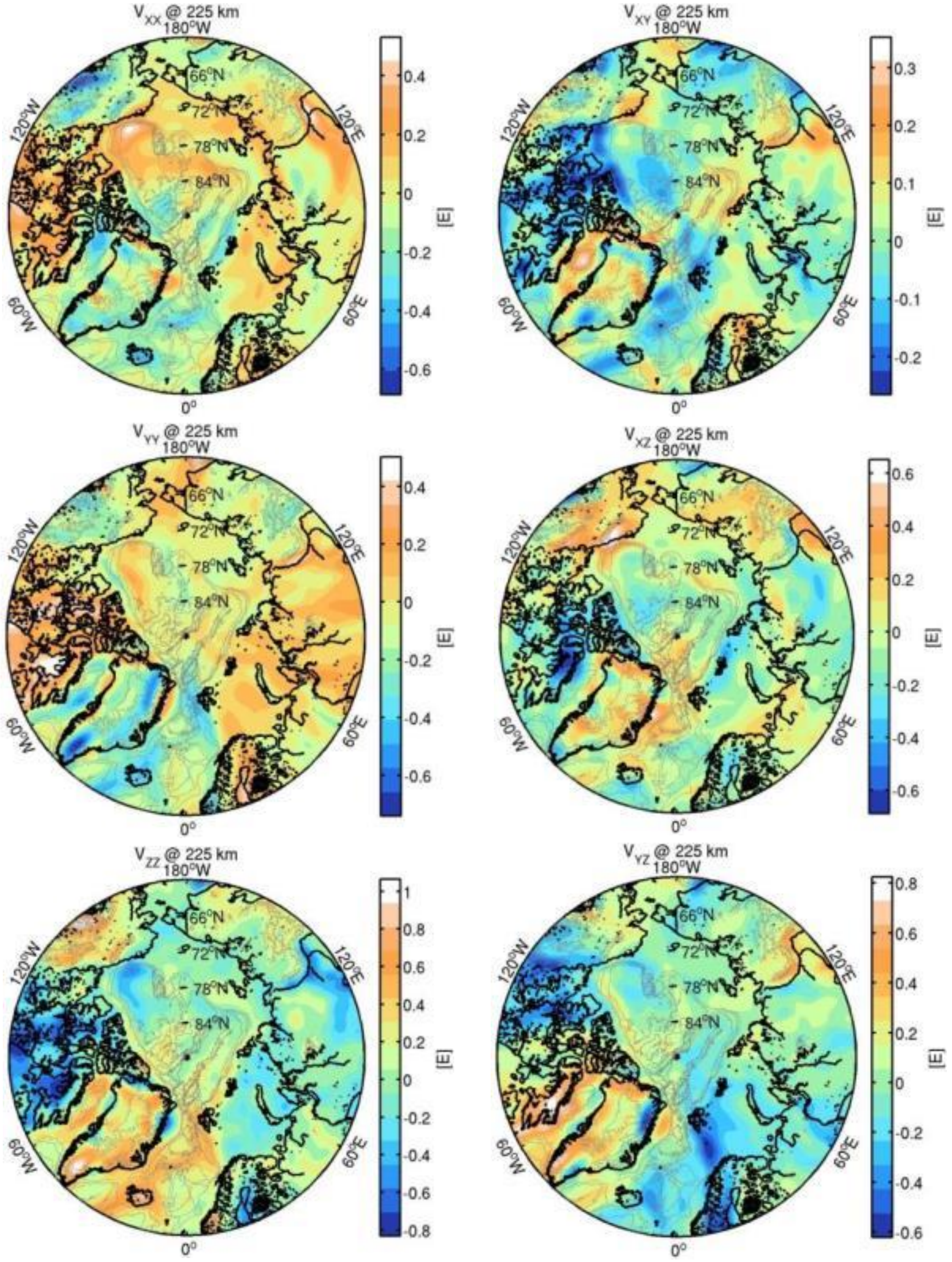
V XX @ 225 km
V XY @ 225 km
V YY @ 225 km
V XZ @ 225 km
V ZZ @ 225 km
V YZ @ 225 km
180°W
120°W
120°E
60°W
60°E
0°
66°N
72°N
78°N
84°N
[E]

South Pole

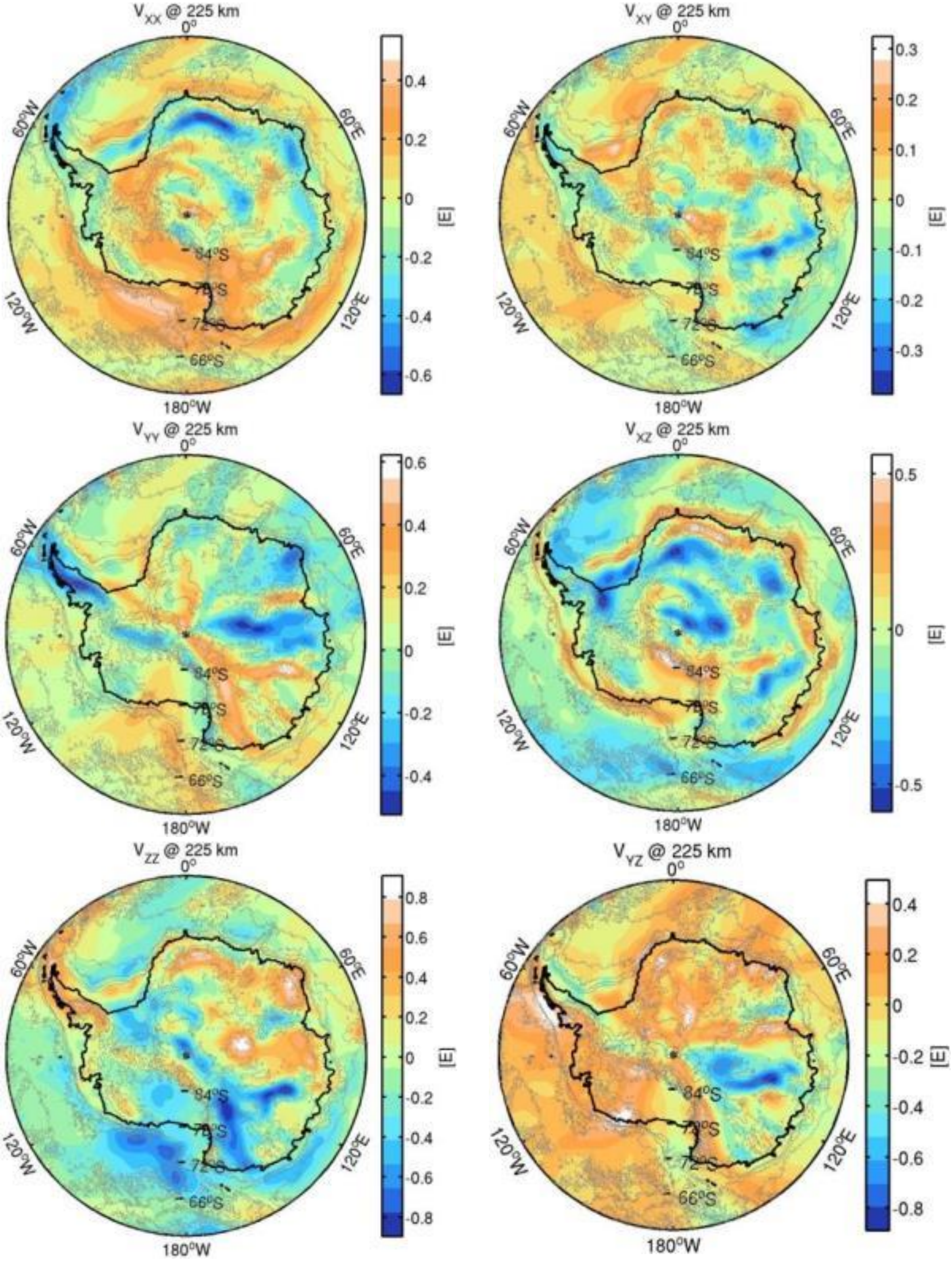
V_{XX} @ 225 km
V_{XY} @ 225 km
V_{YY} @ 225 km
V_{XZ} @ 225 km
V_{ZZ} @ 225 km
V_{YZ} @ 225 km
0°
60°W
60°E
120°W
120°E
180°W
84°S
78°S
72°S
66°S
[E]

Gravity gradient grids @ 225 km altitude with topographic reduction

The gravity gradient signal after topographic mass reduction at 225 km altitude is shown for longitude-latitude blocks of 60° × 30° for longitudes -180° ≤ λ ≤ 180° and latitudes -75° ≤ ϕ ≤ 75°. The North and South Pole are shown separately. The topographic mass reduction has been done using a spherical harmonic model for rock, water and ice density [44], where we used a maximum spherical harmonic degree of L = 360 to be consistent with the gravity gradient resolution. This global correction enhances the signal of the internal structure of the Earth and is the equivalent to a Bouguer gravity anomaly. The gradient signal with respect to the WGS84 reference ellipsoid is shown in the order as shown in the table below, starting in the south-west. The colour scale in each region and for each gradient is adapted to the min/max values in that region. Topography and bathymetry contour lines are shown every 1000 m and were derived from ETOPO1 [47]. The Lambert projection is used for the patches centred at ϕ = ±60°, a stereographic projection is used for the North and South Pole, and all other patches use the Mercator projection.

V_{XX}	V_{XY}
V_{YY}	V_{XZ}
V_{ZZ}	V_{YZ}

The geolocation of all patches is shown in the figure below.

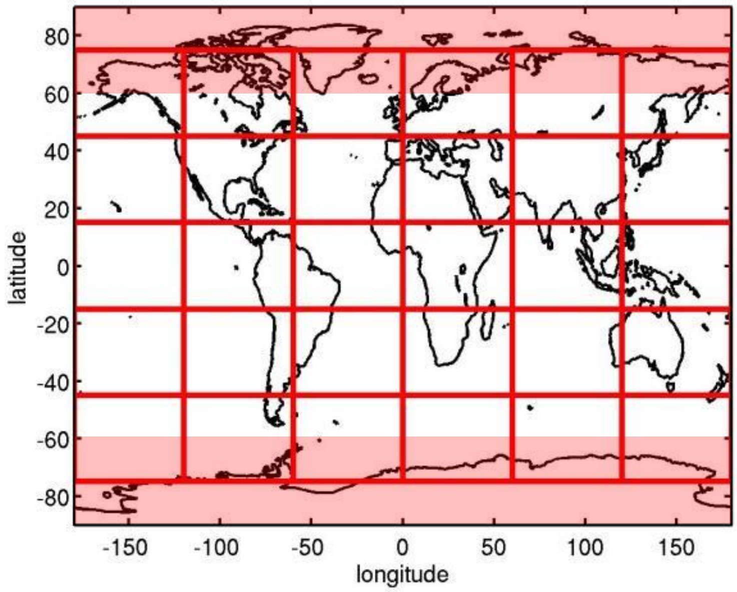

-180° ≤ λ ≤ -120°, -75° ≤ φ ≤ -45°, with topographic reduction

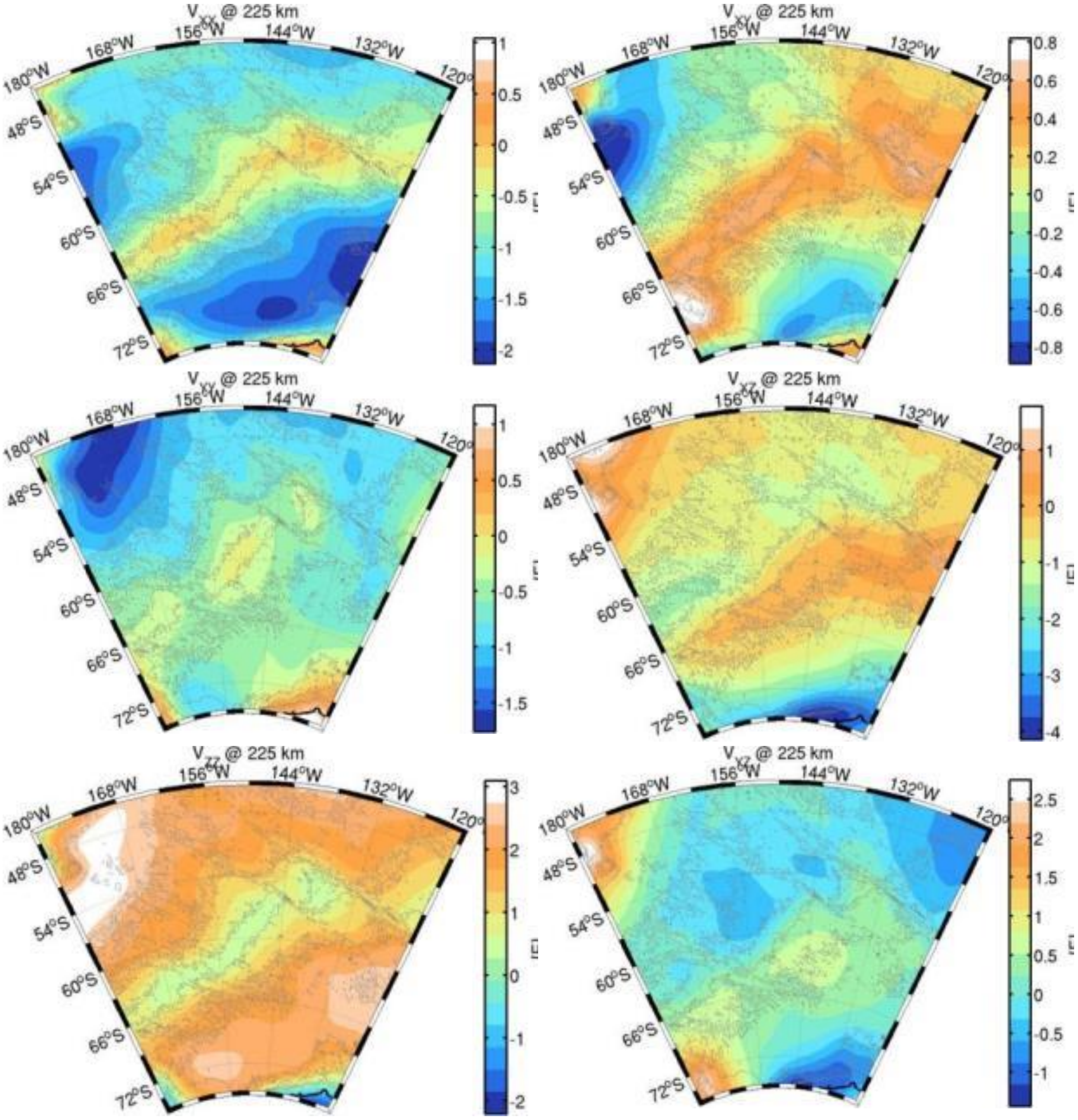

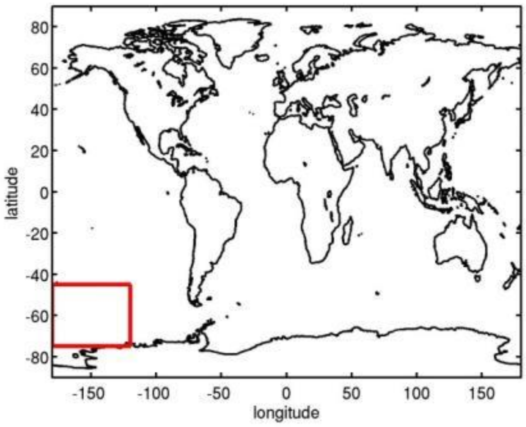

-120° ≤ λ ≤ -60°, -75° ≤ φ ≤ -45°, with topographic reduction

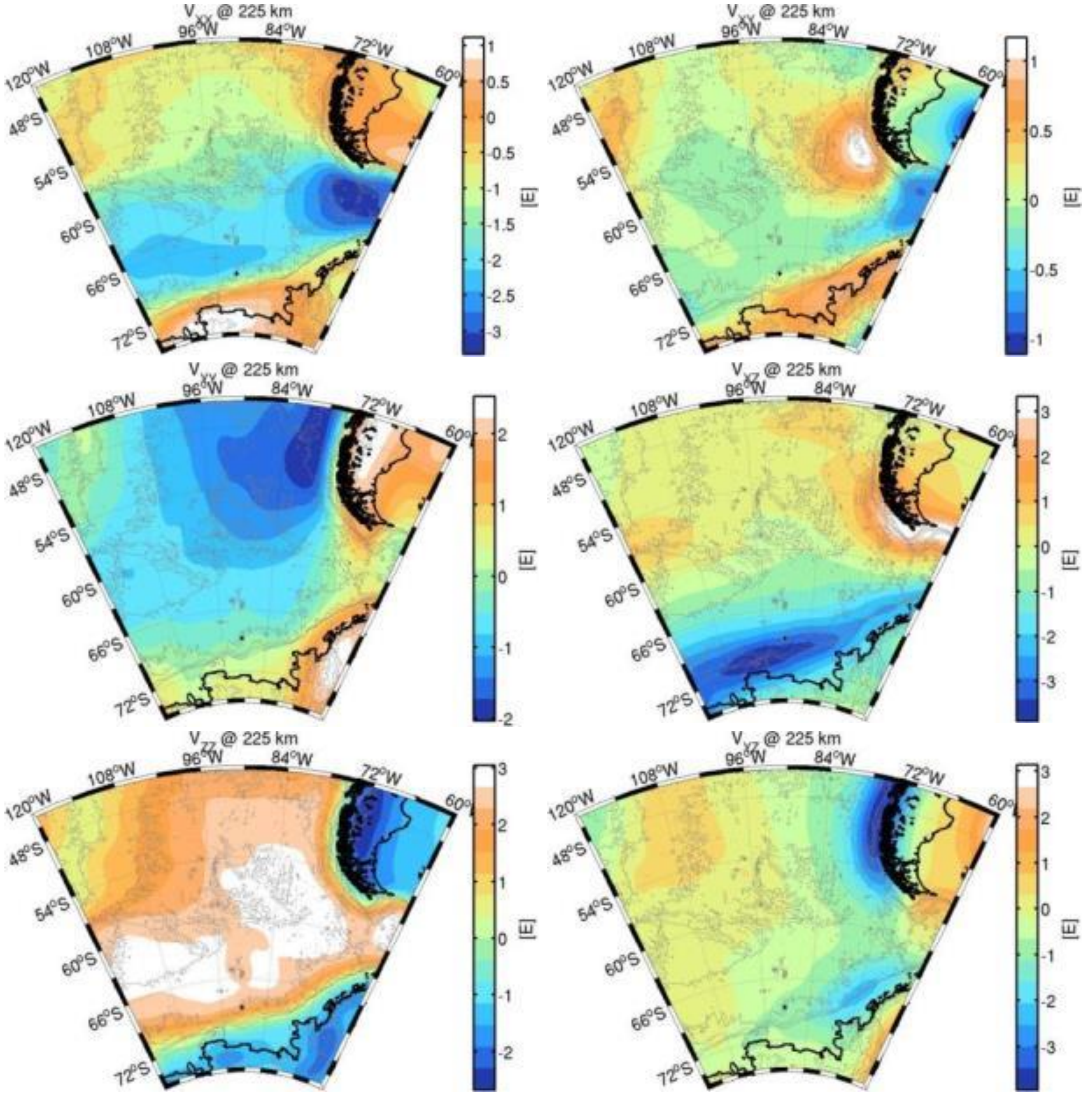

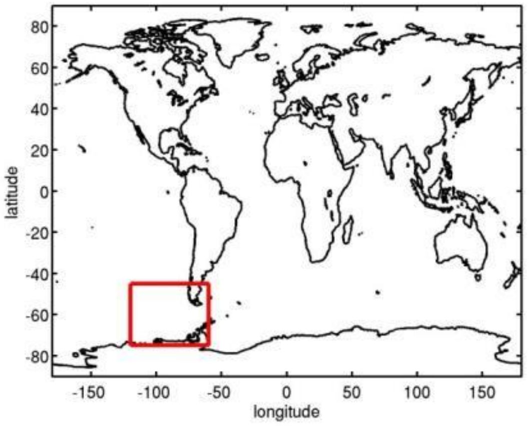

-60° ≤ λ ≤ 0°, -75° ≤ φ ≤ -45°, with topographic reduction

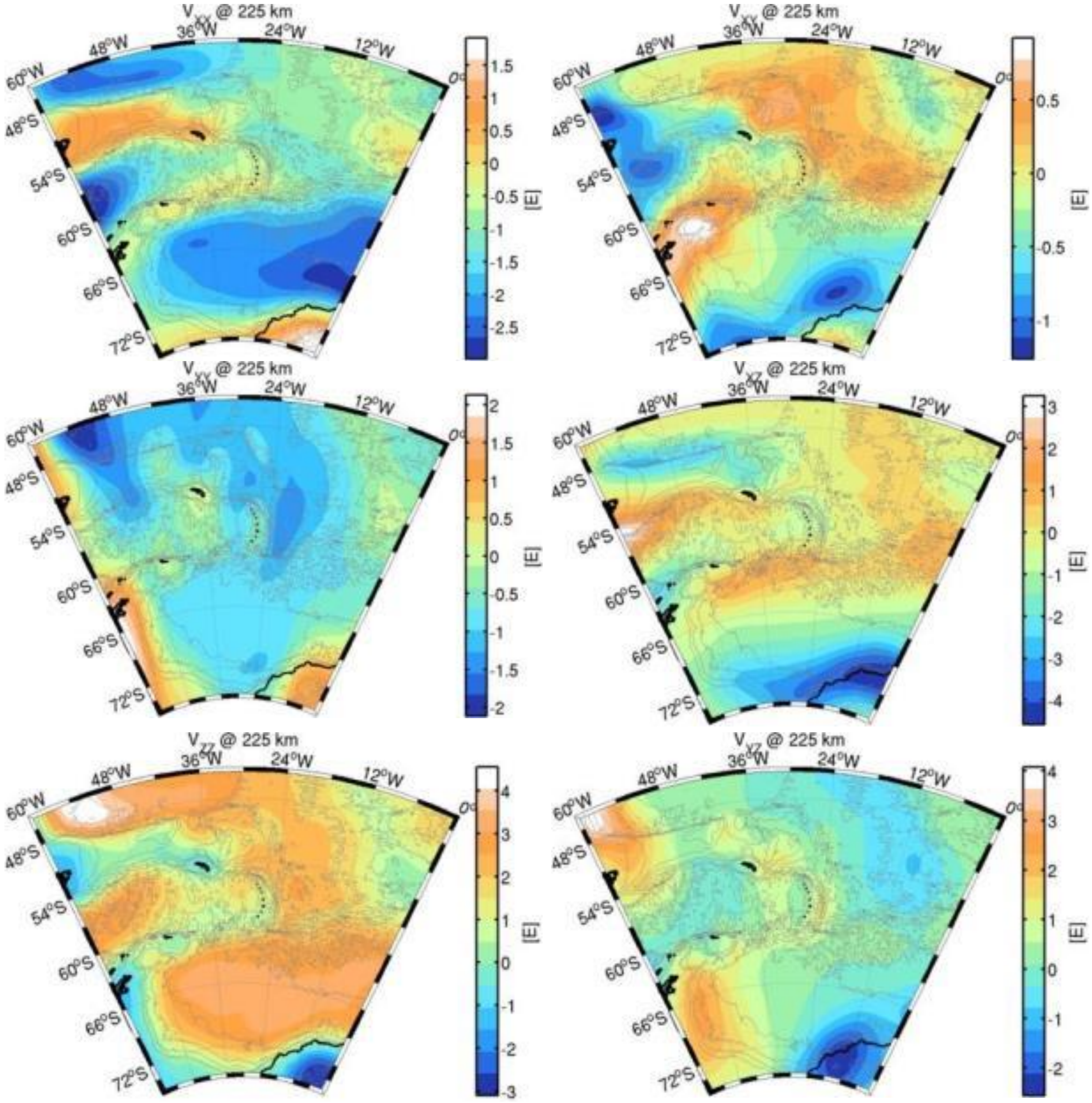

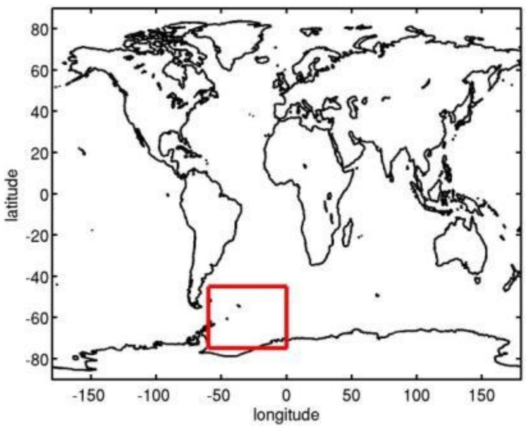

0° ≤ λ ≤ 60°, -75° ≤ φ ≤ -45°, with topographic reduction

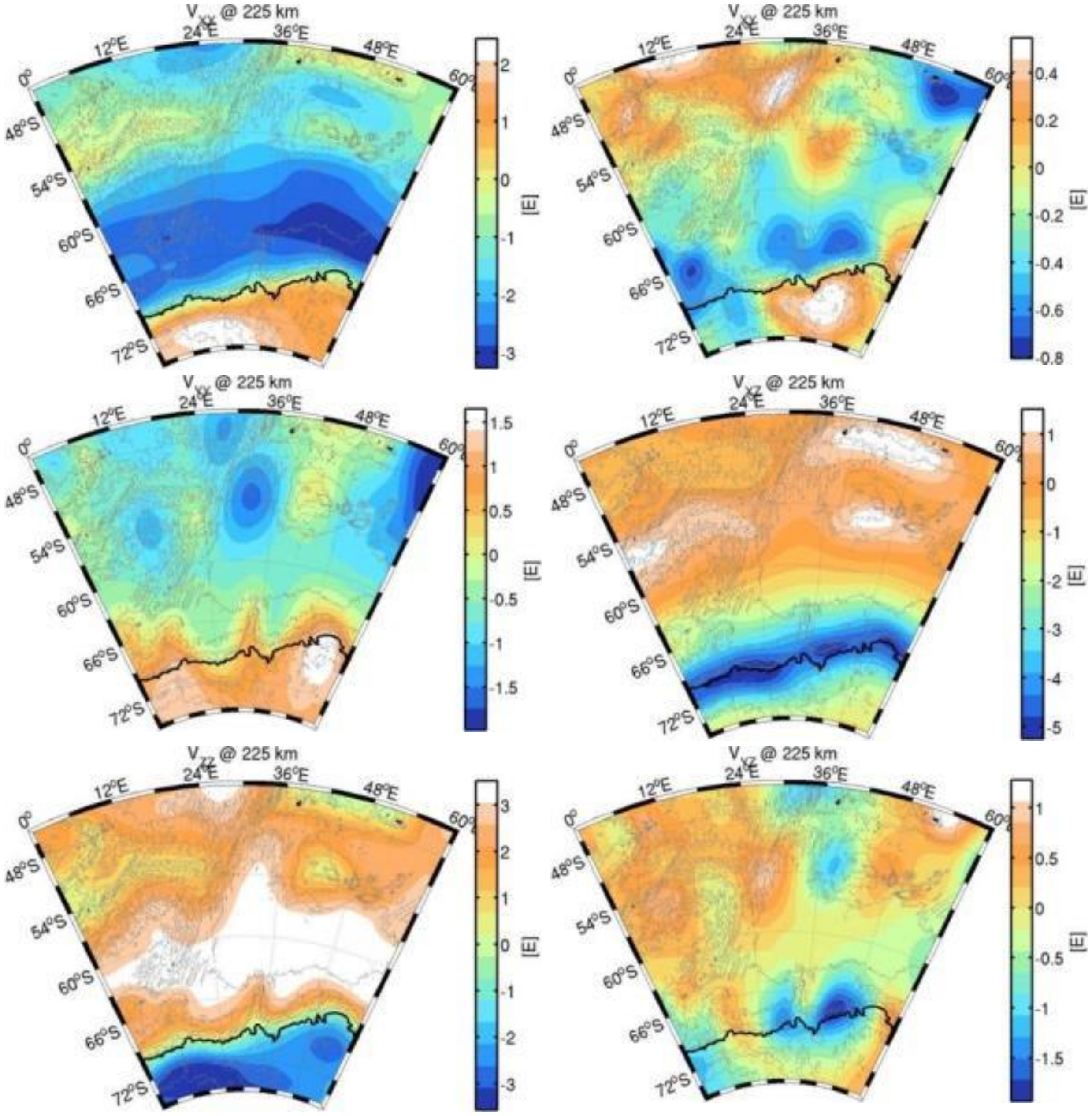

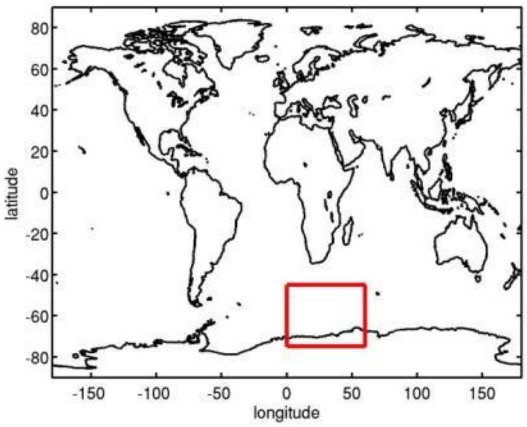

60° ≤ λ ≤ 120°, -75° ≤ φ ≤ -45°, with topographic reduction

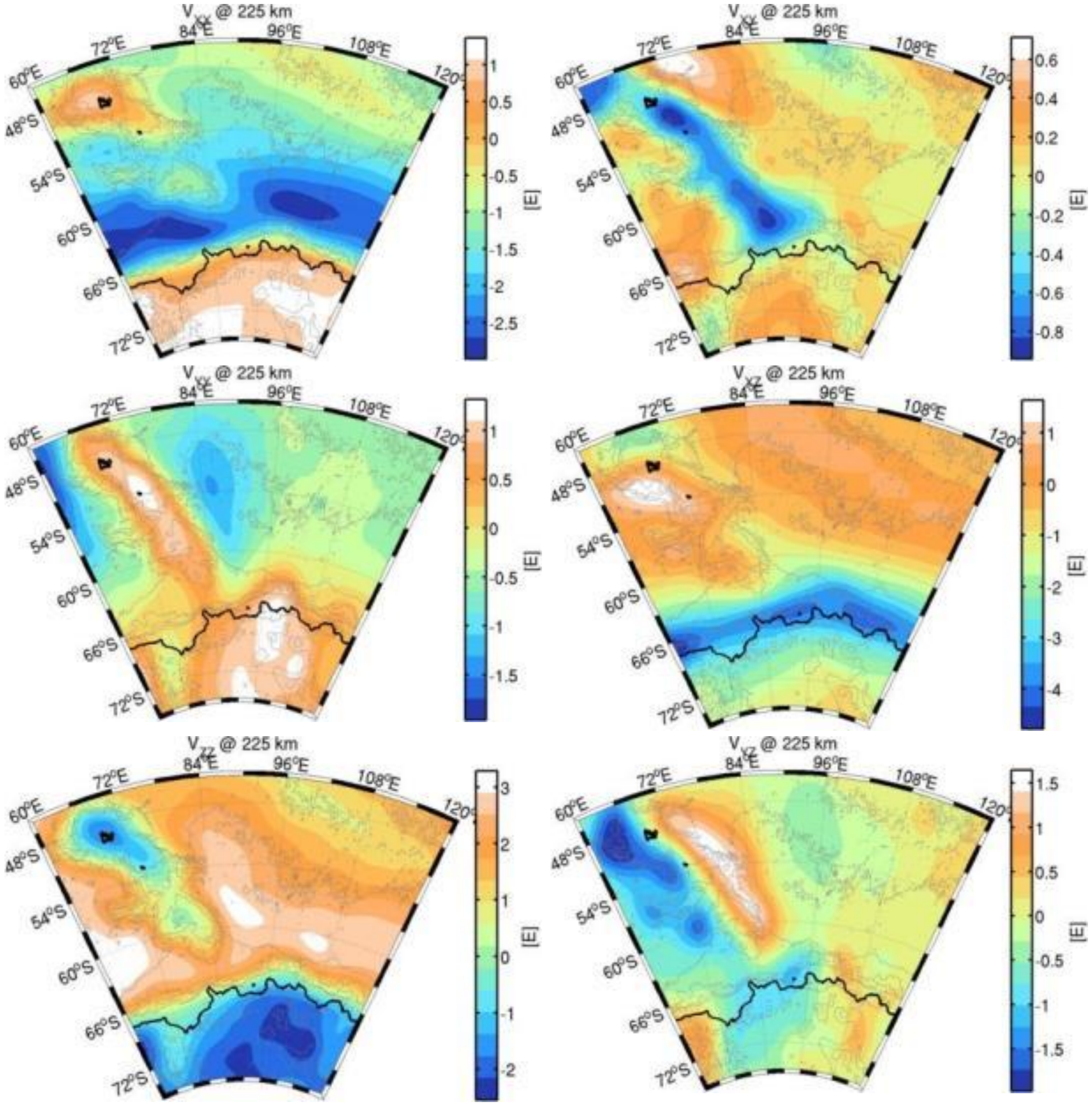

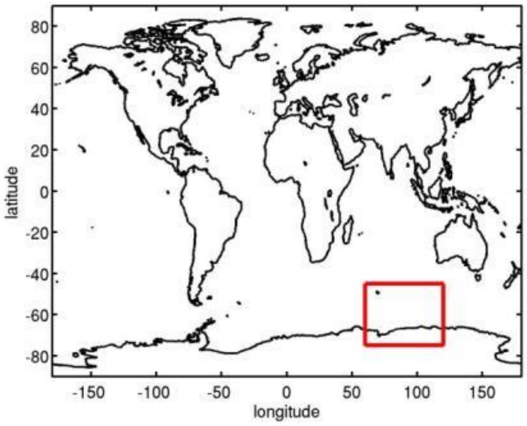

120° ≤ λ ≤ 180°, -75° ≤ φ ≤ -45°, with topographic reduction

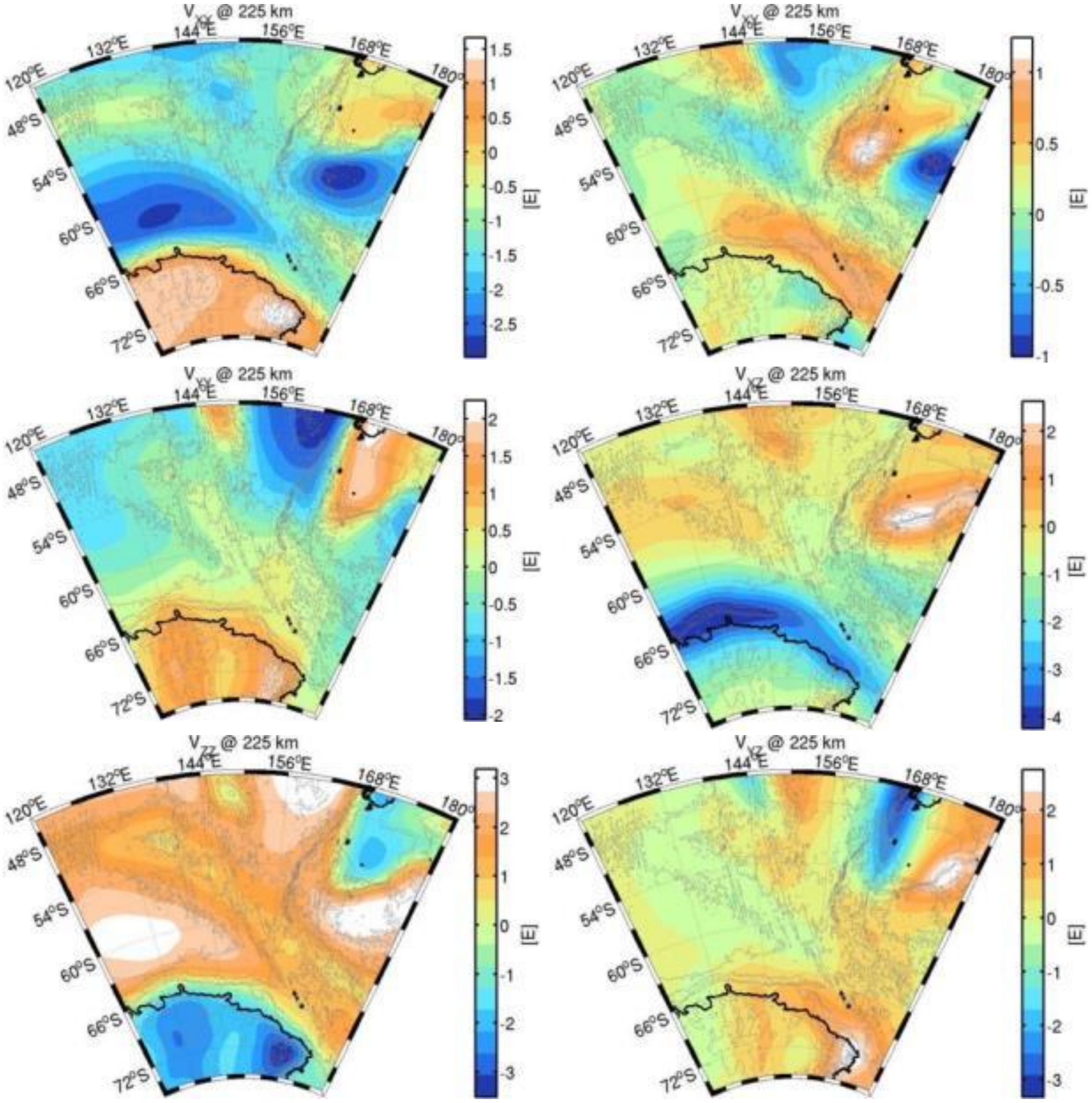

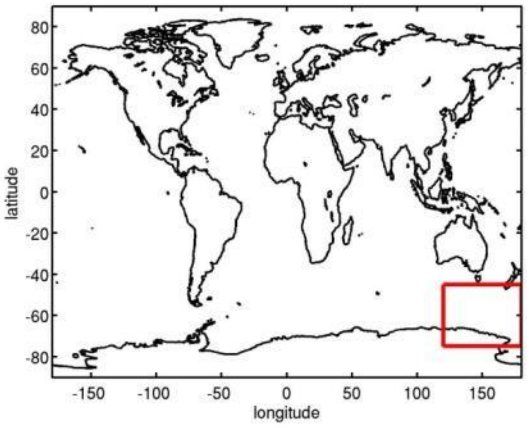

-180° ≤ λ ≤ -120°, -45° ≤ φ ≤ -15°, with topographic reduction

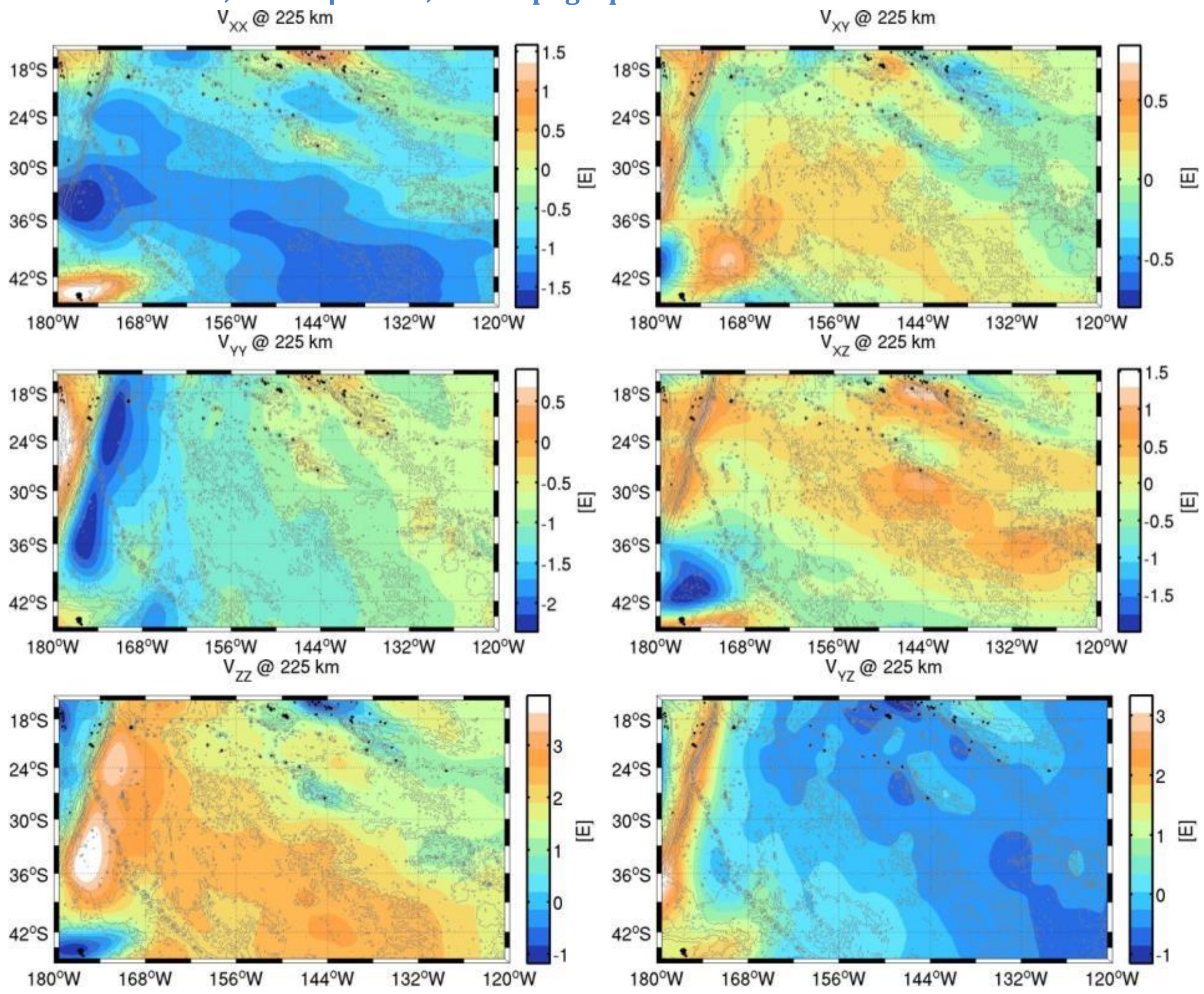

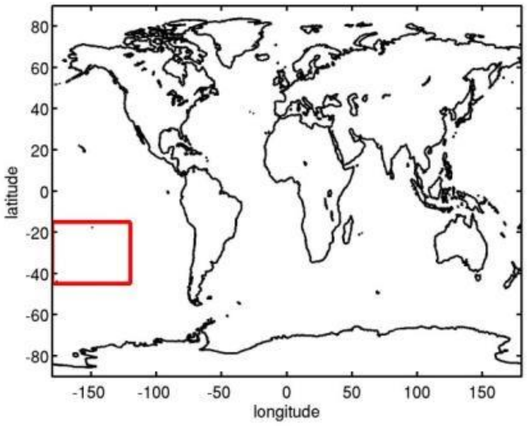

-120° ≤ λ ≤ -60°, -45° ≤ φ ≤ -15°, with topographic reduction

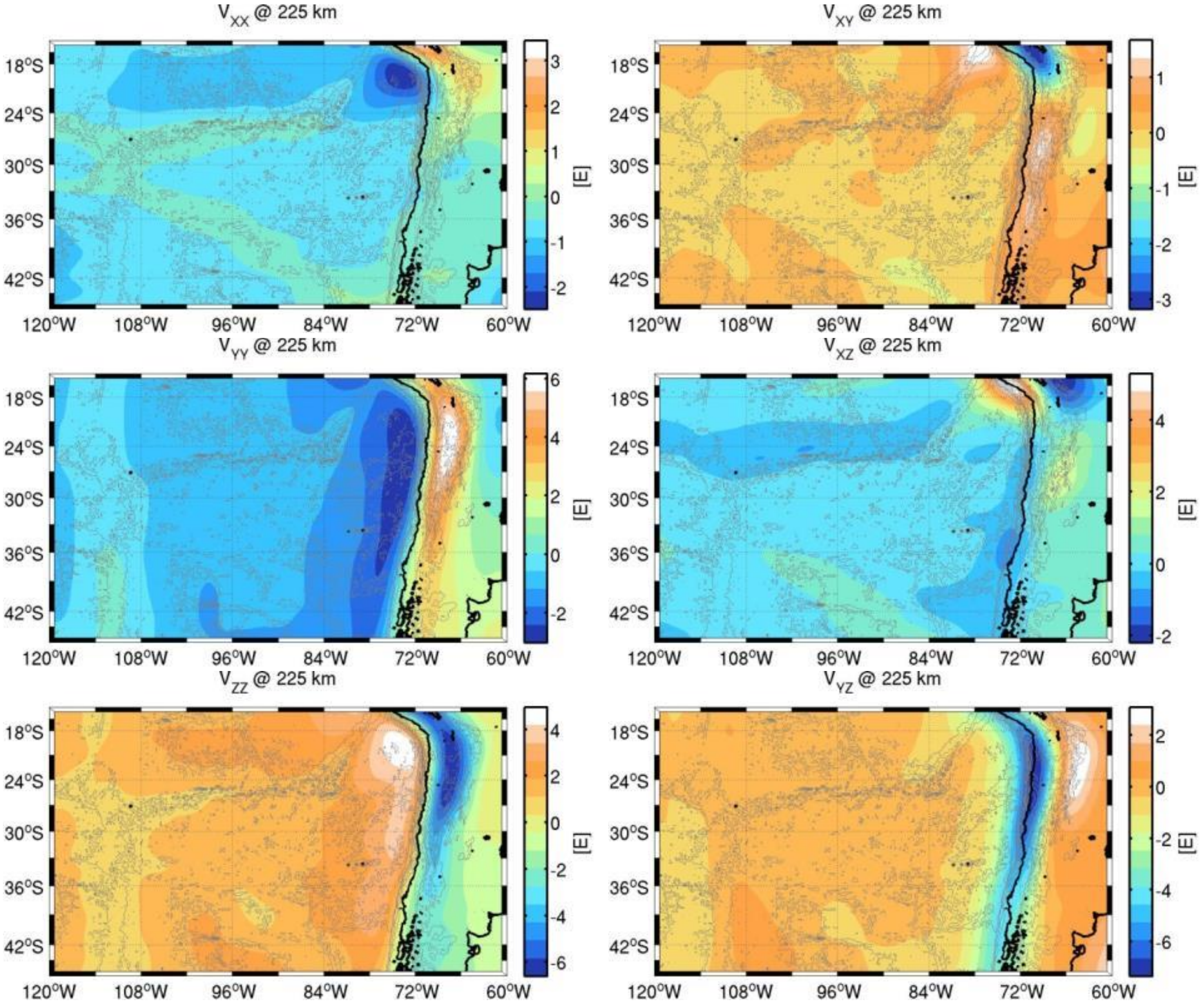

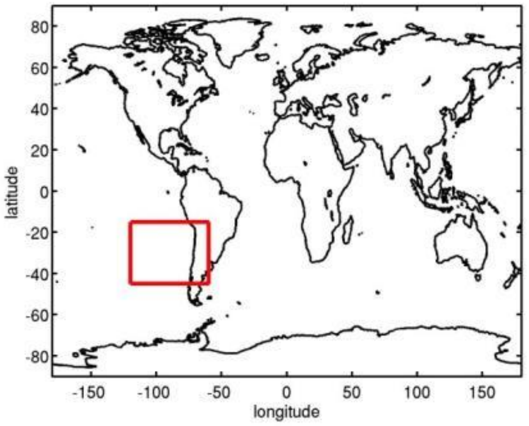

$-60° \leq \lambda \leq 0°, -45° \leq \varphi \leq -15°$, with topographic reduction

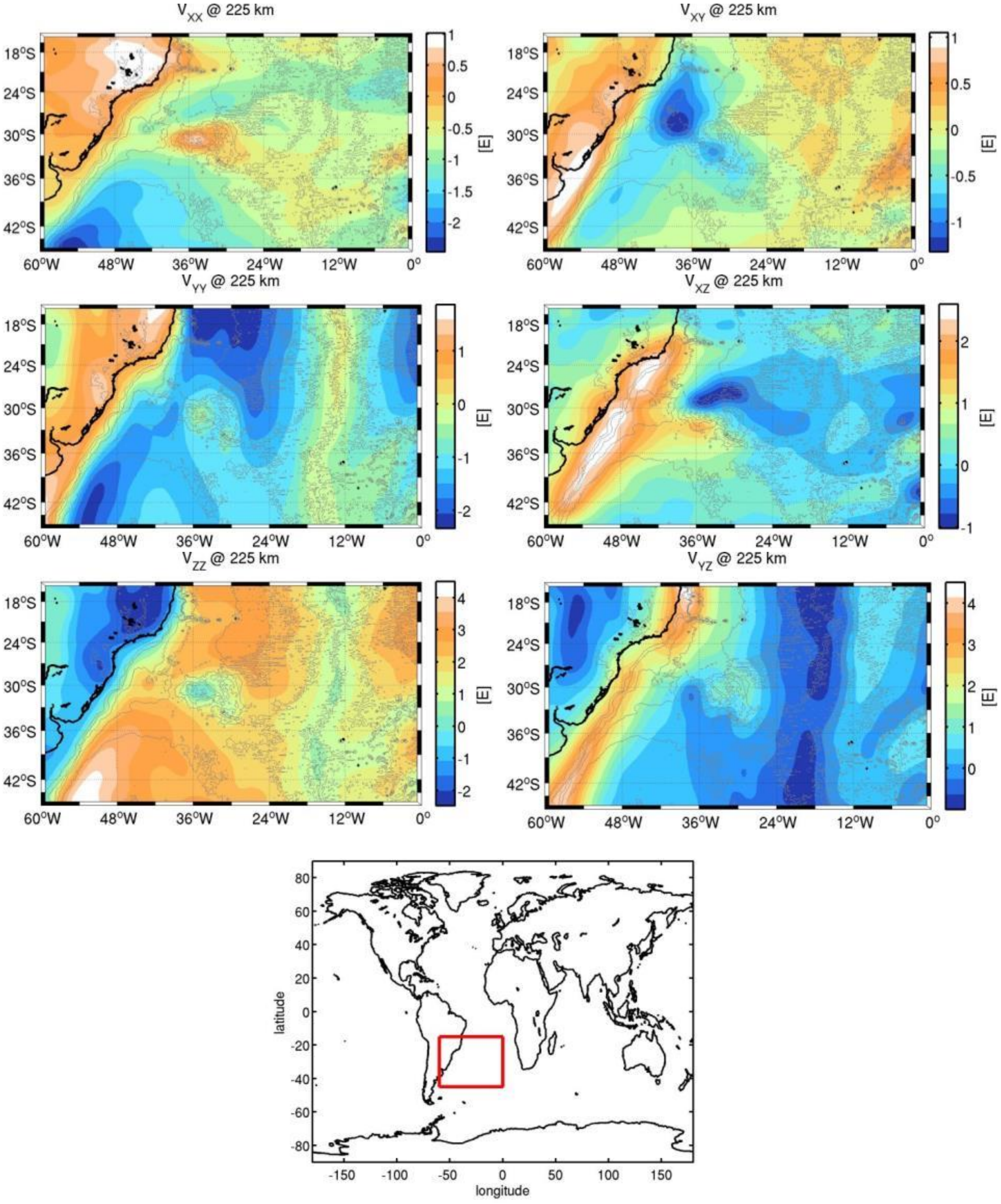

0° ≤ λ ≤ 60°, -45° ≤ φ ≤ -15°, with topographic reduction

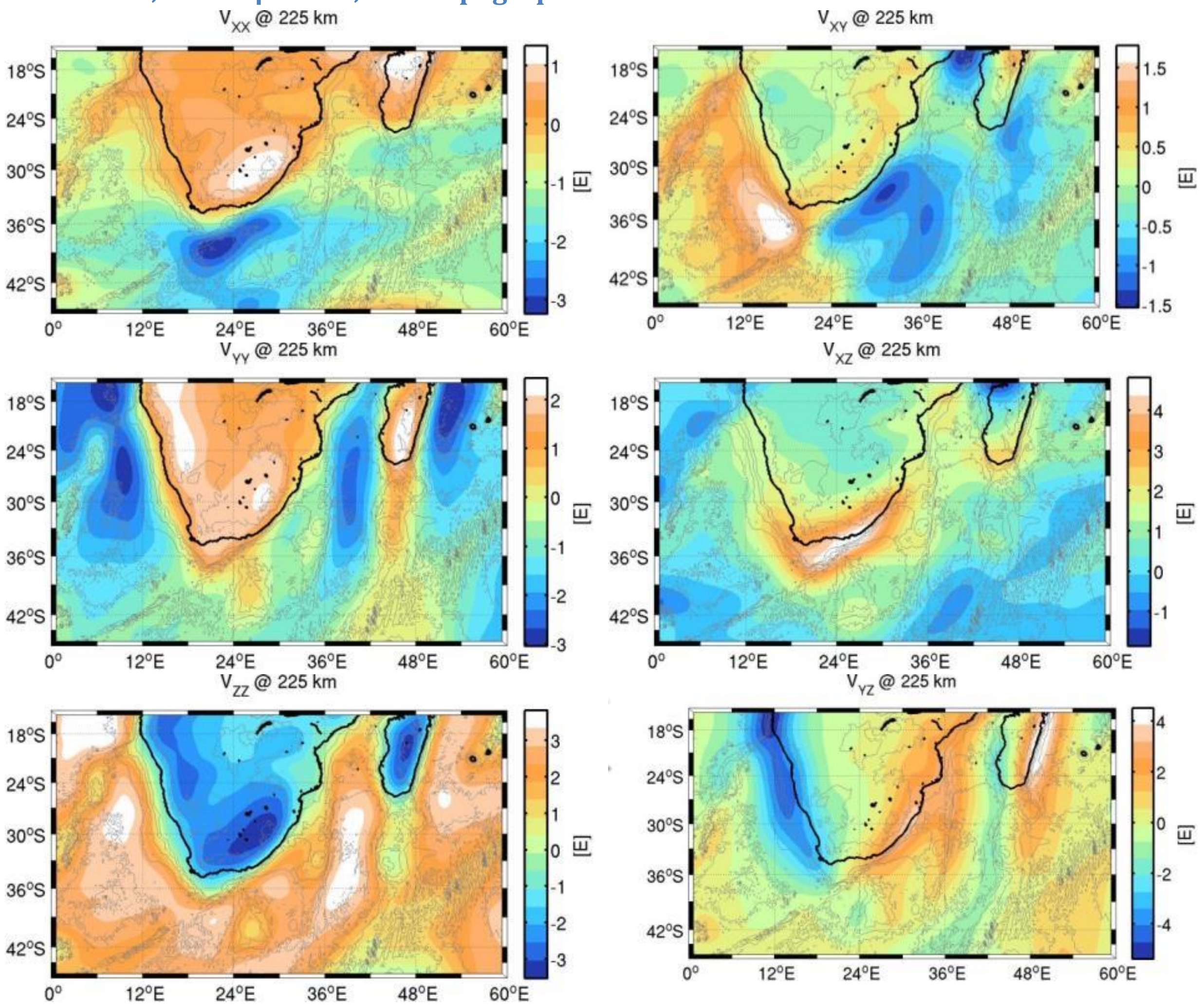

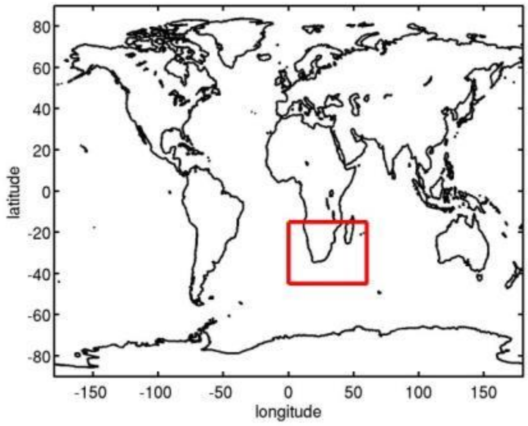

60° ≤ λ ≤ -120°, -45° ≤ φ ≤ -15°, with topographic reduction

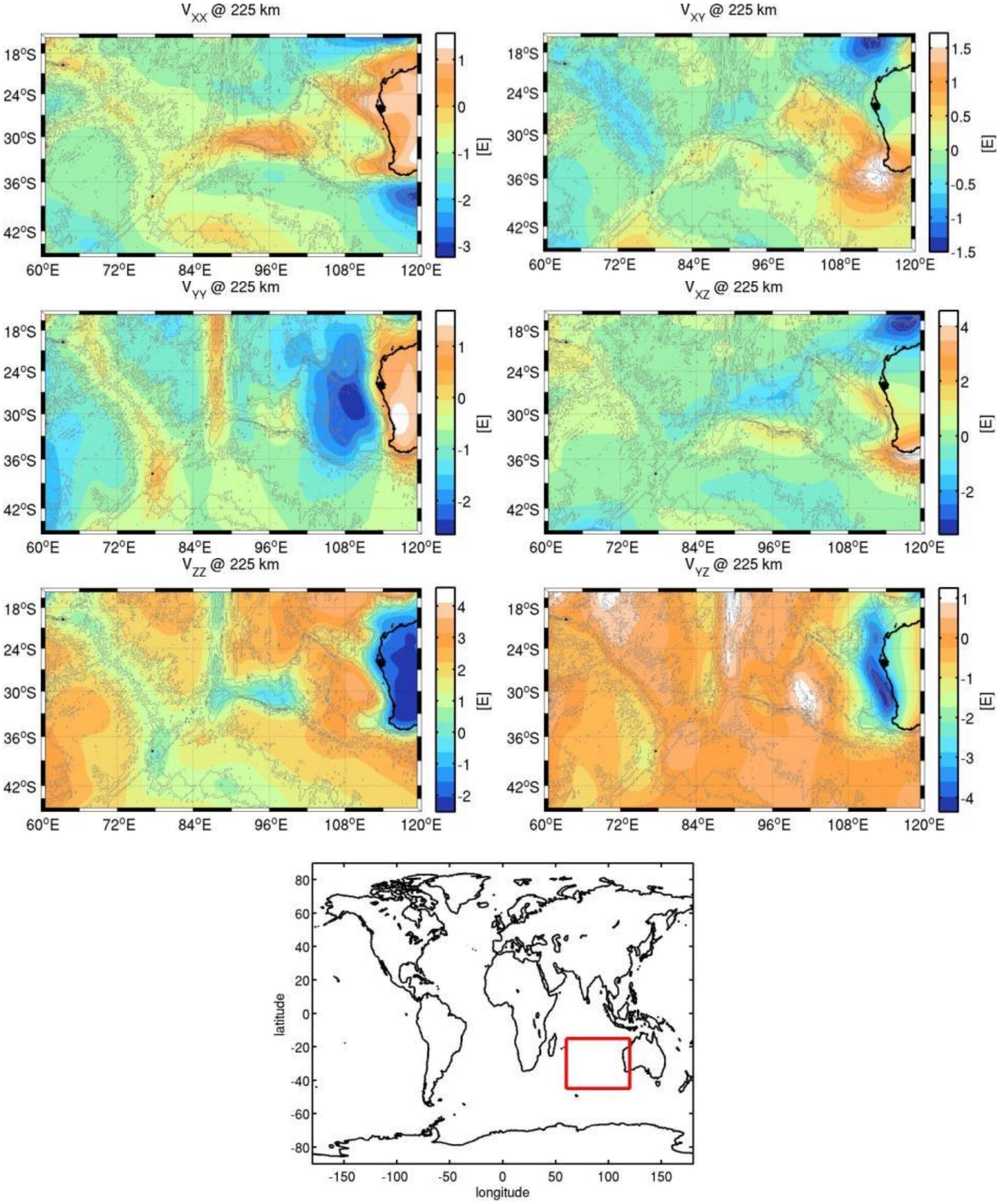

120° ≤ λ ≤ 180°, -45° ≤ φ ≤ -15°, with topographic reduction

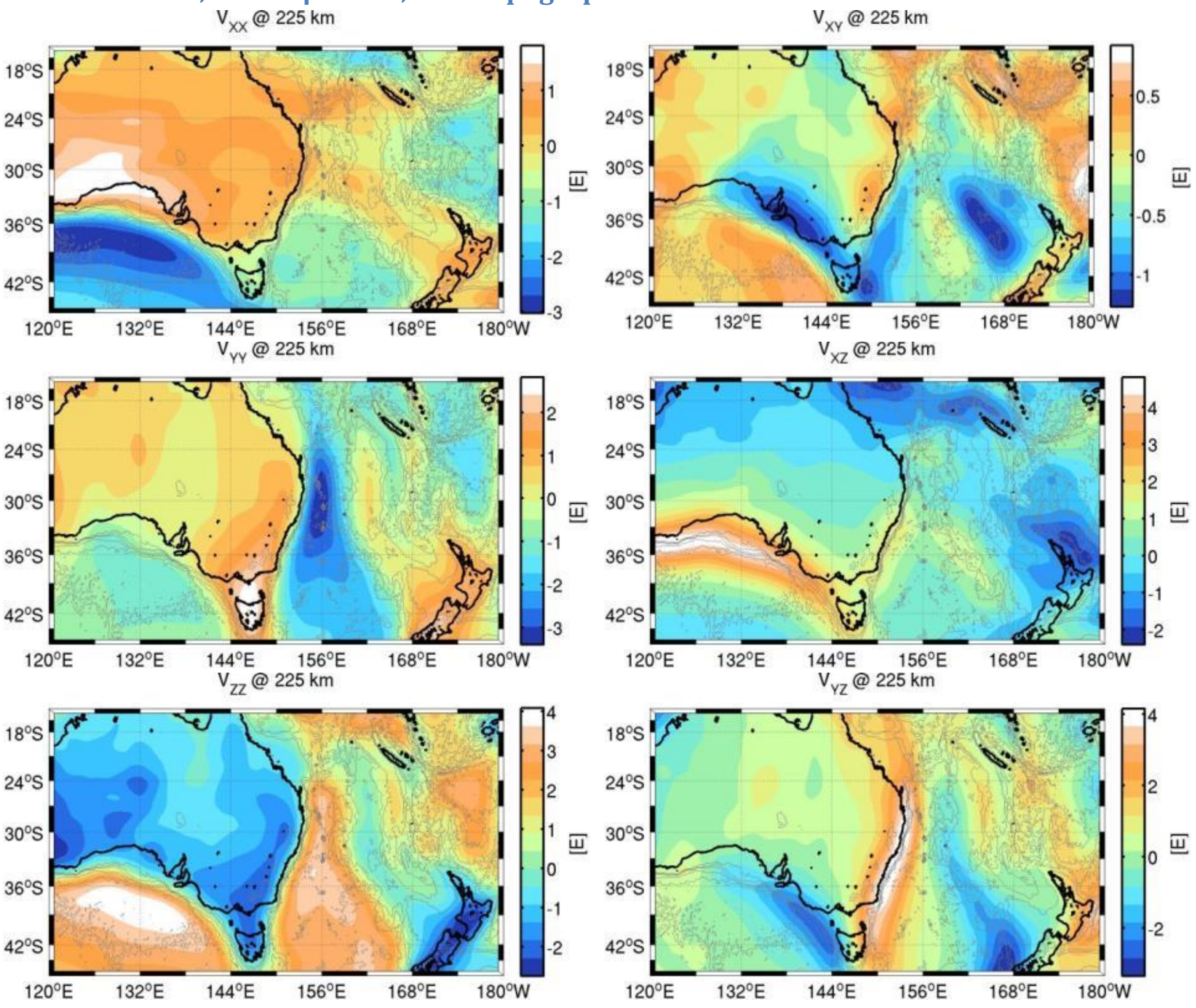

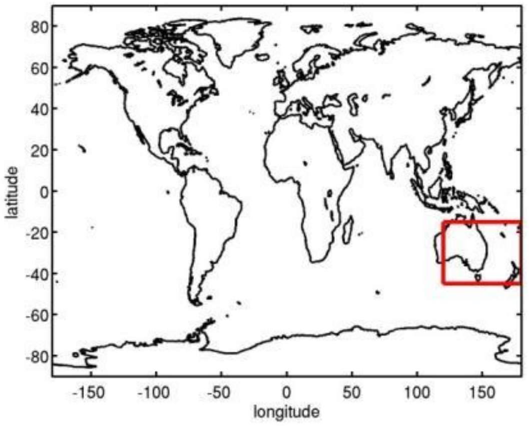

-180° ≤ λ ≤ -120°, -15° ≤ φ ≤ 15°, with topographic reduction

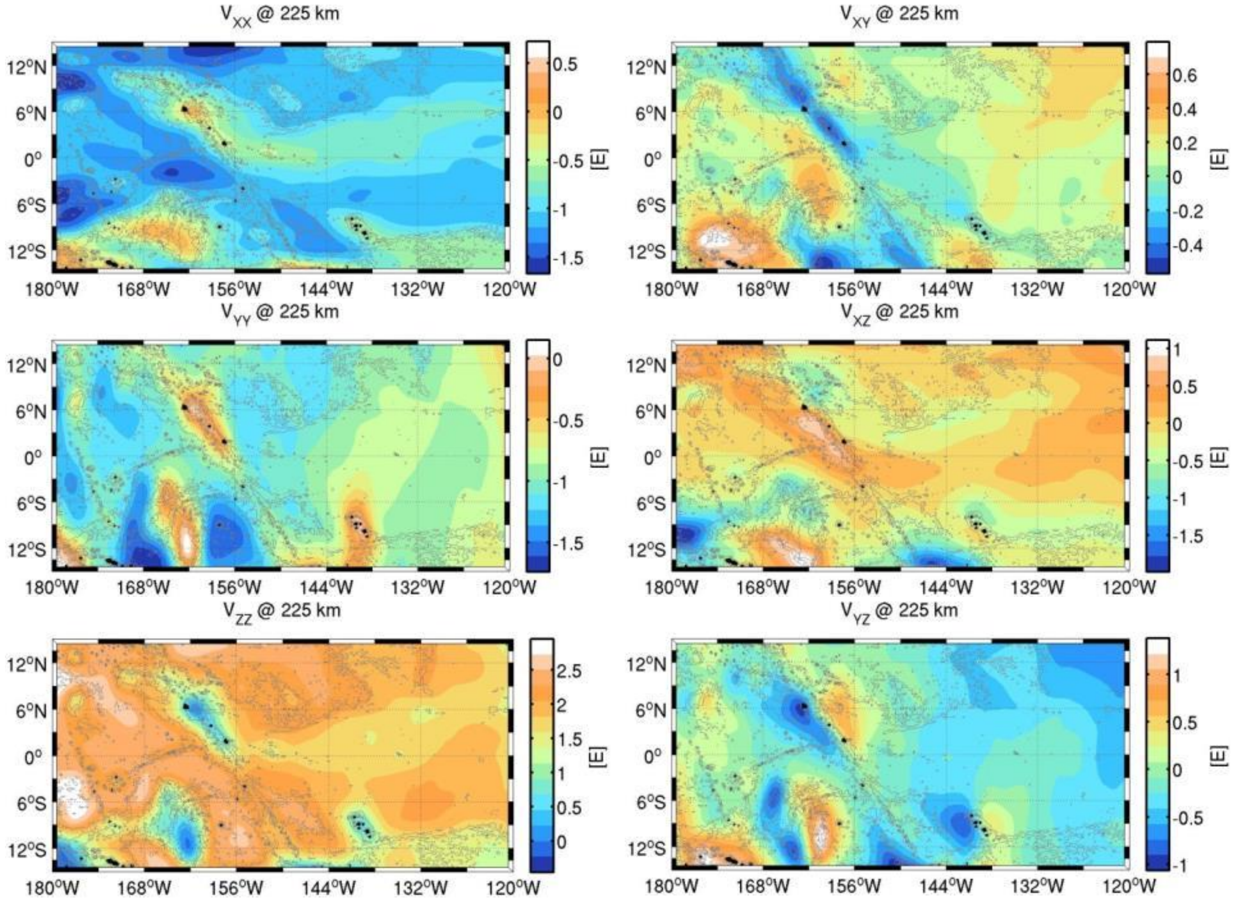

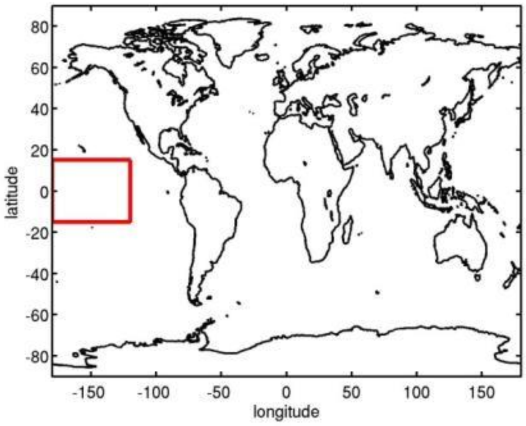

-120° ≤ λ ≤ -60°, -15° ≤ φ ≤ 15°, with topographic reduction

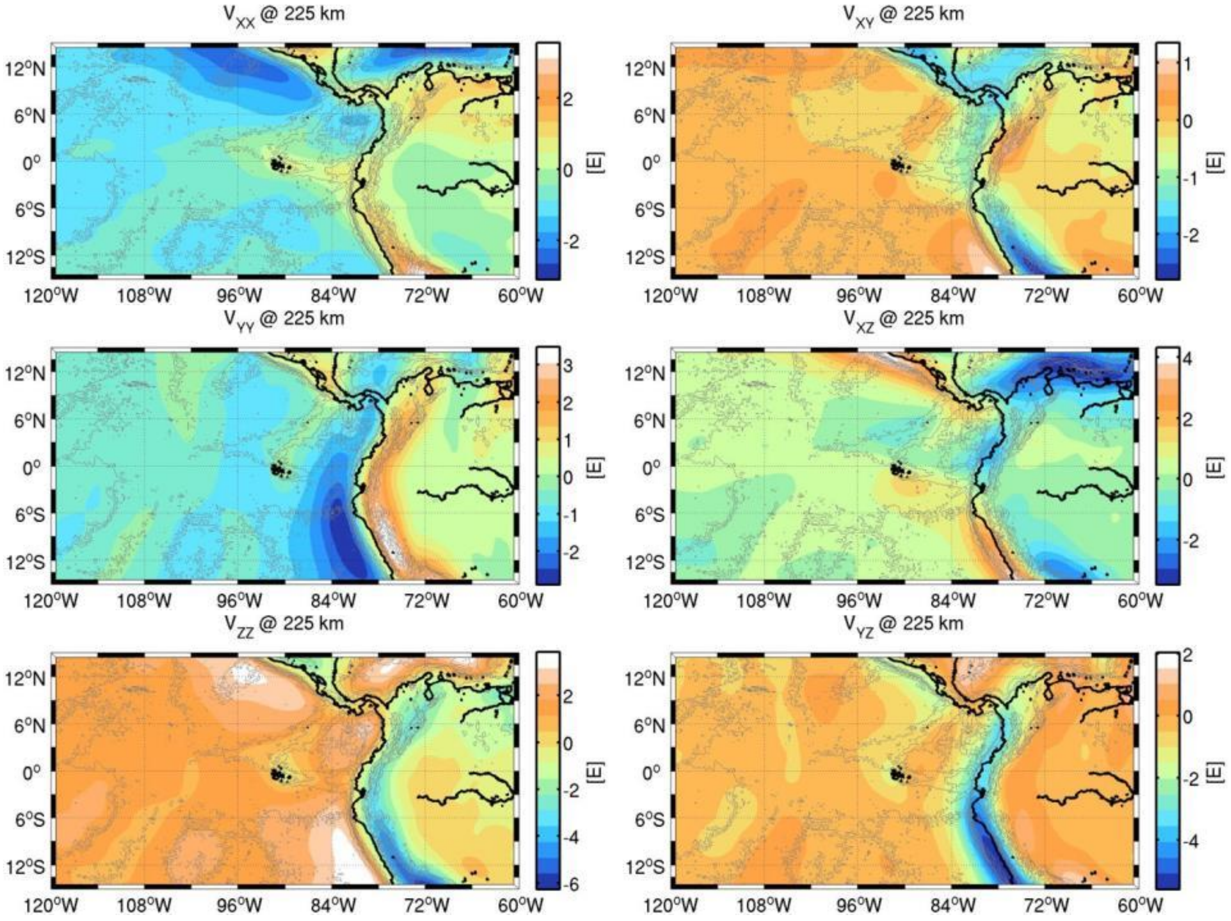

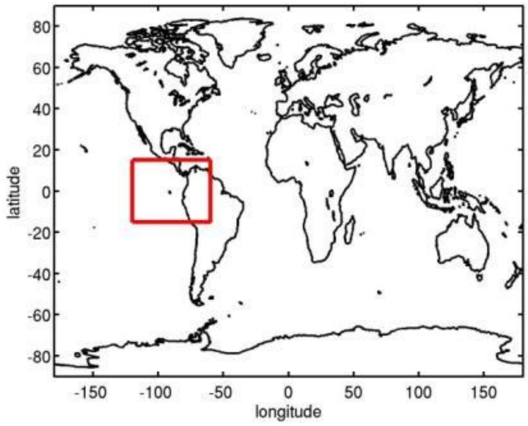

-60° ≤ λ ≤ 0°, -15° ≤ φ ≤ 15°, with topographic reduction

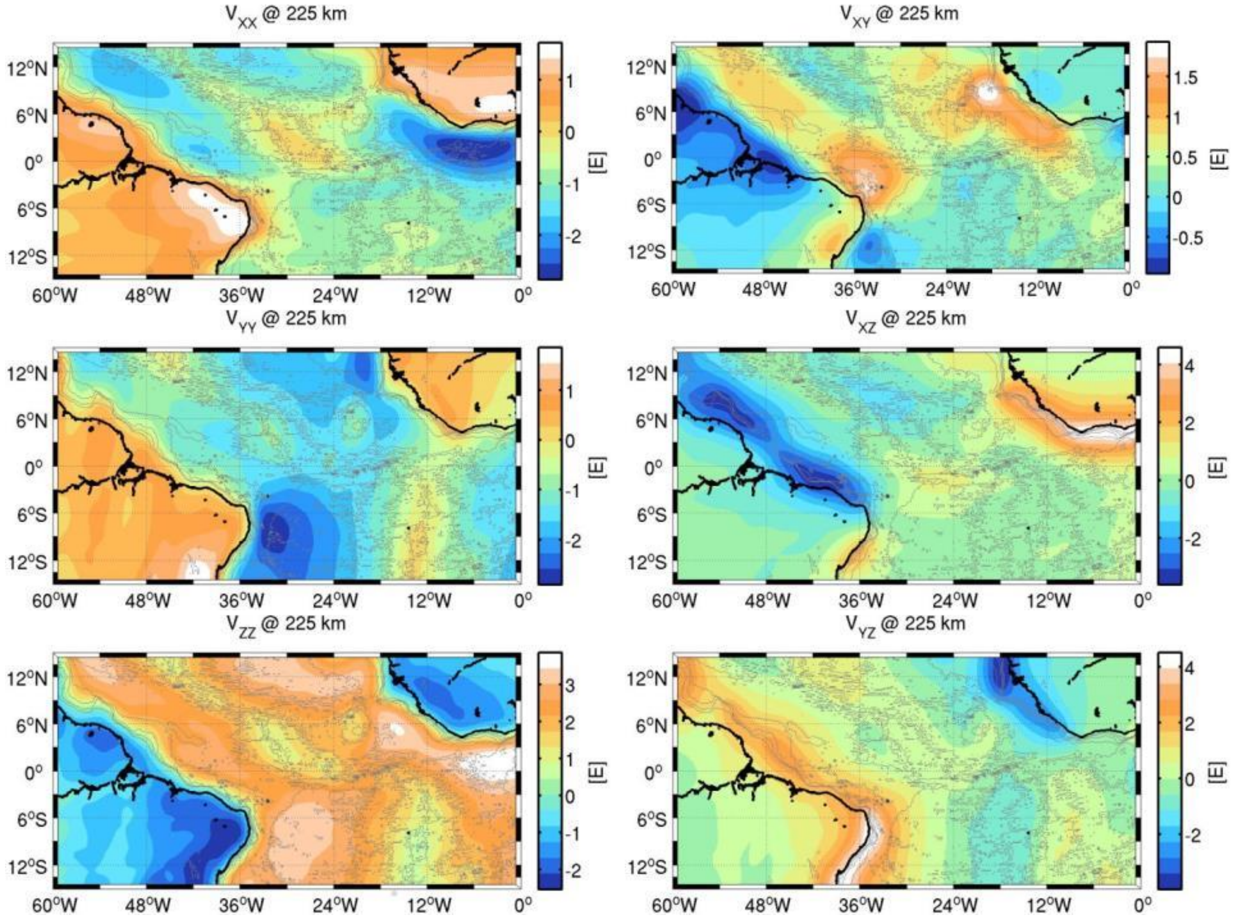

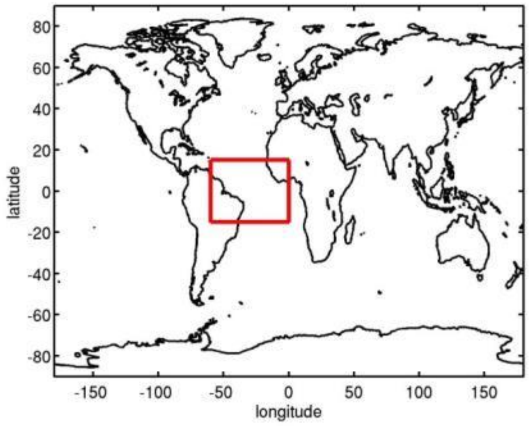

0° ≤ λ ≤ 60°, -15° ≤ φ ≤ 15°, with topographic reduction

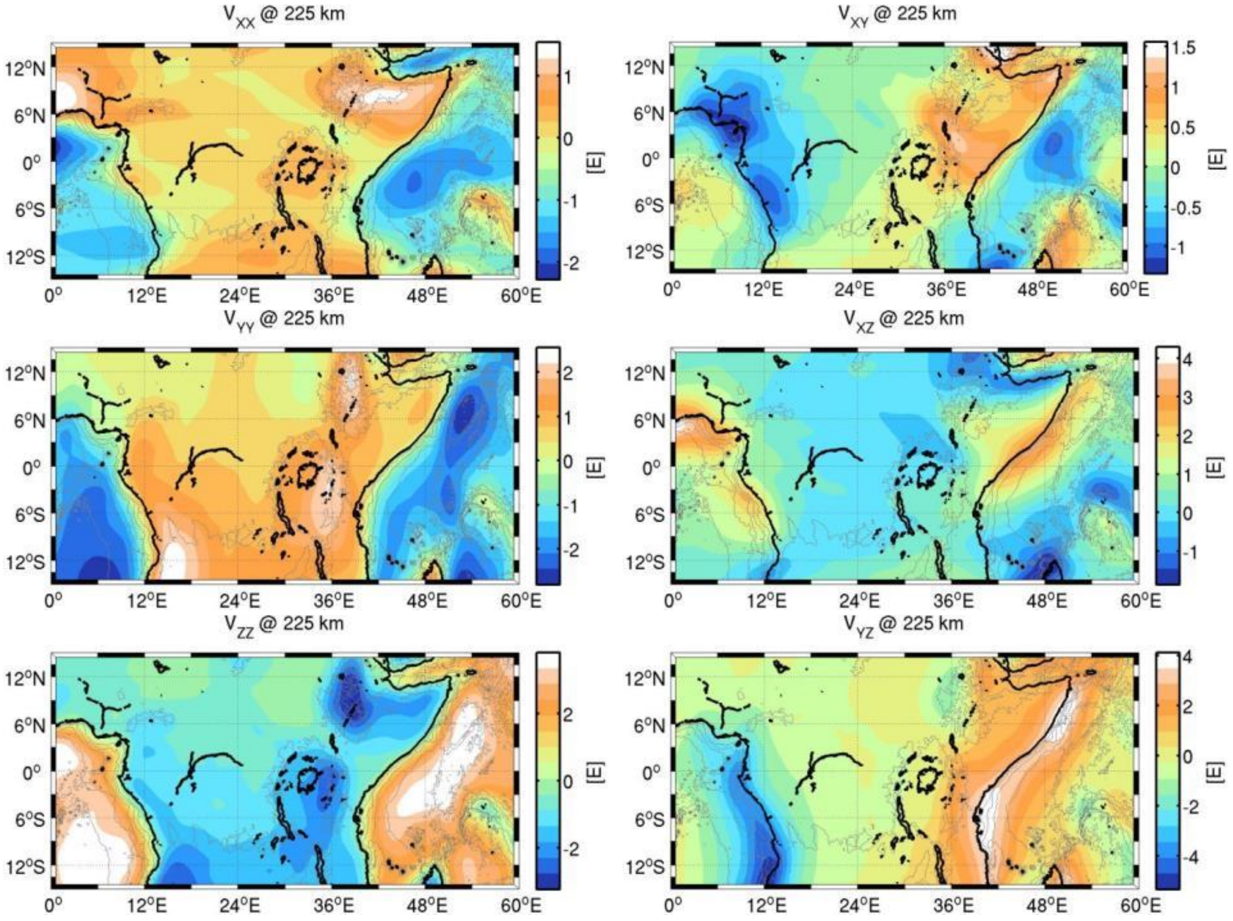

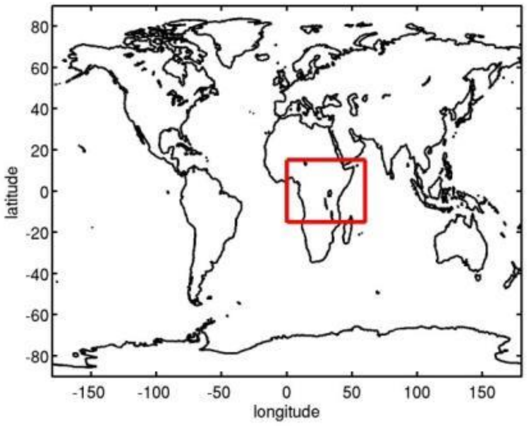

60° ≤ λ ≤ 120°, -15° ≤ φ ≤ 15°, with topographic reduction

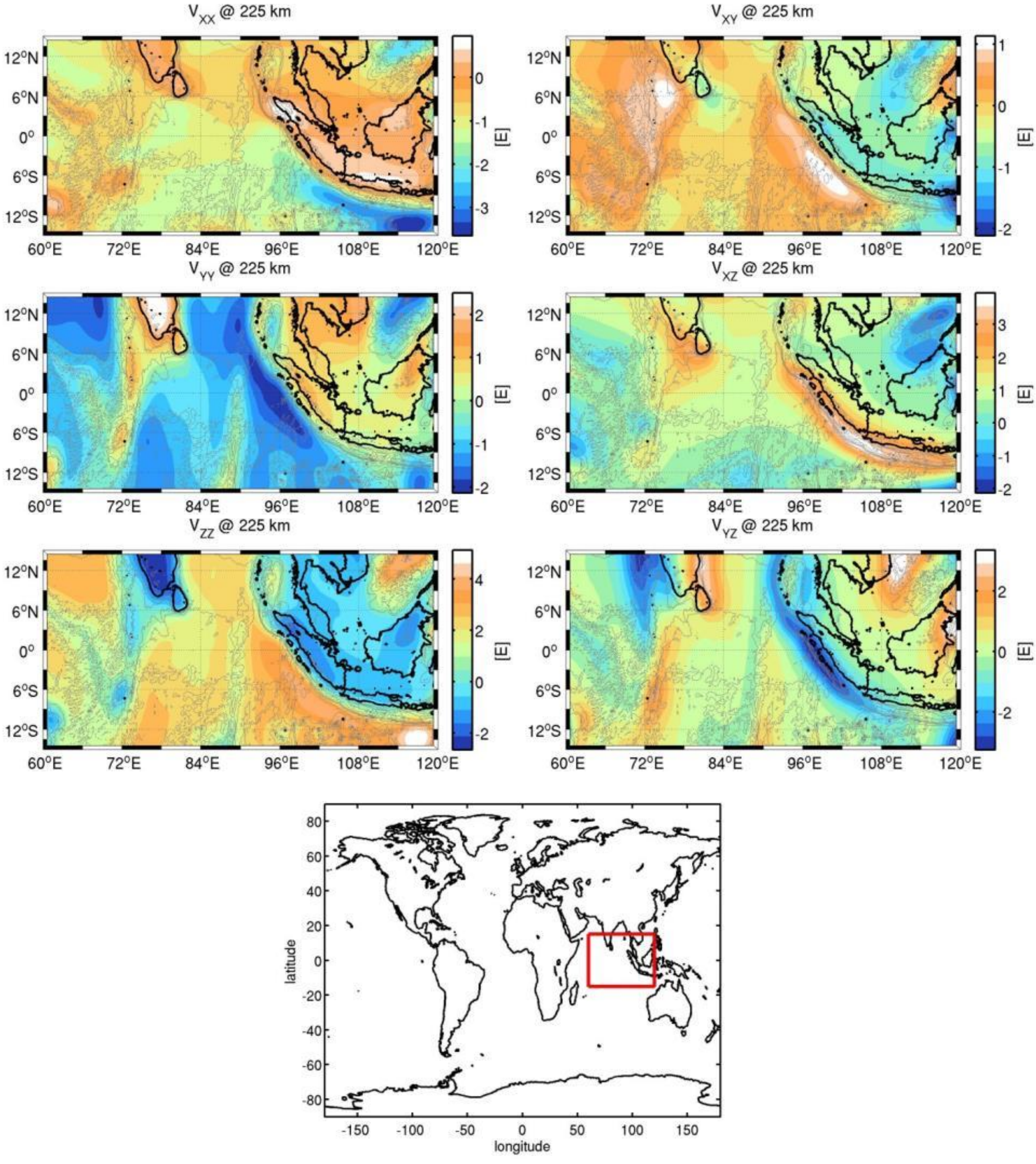

120° ≤ λ ≤ 180°, -15° ≤ φ ≤ 15°, with topographic reduction

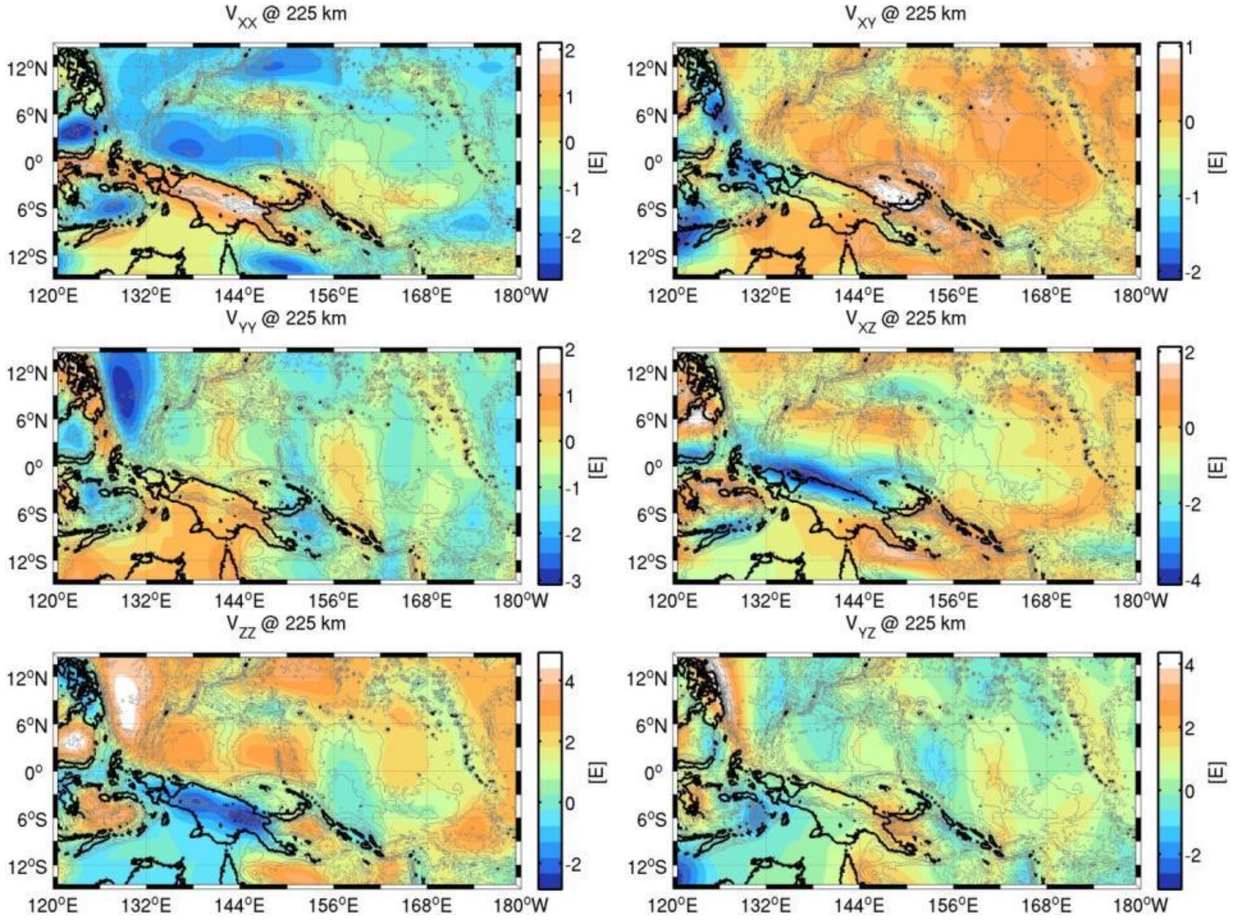

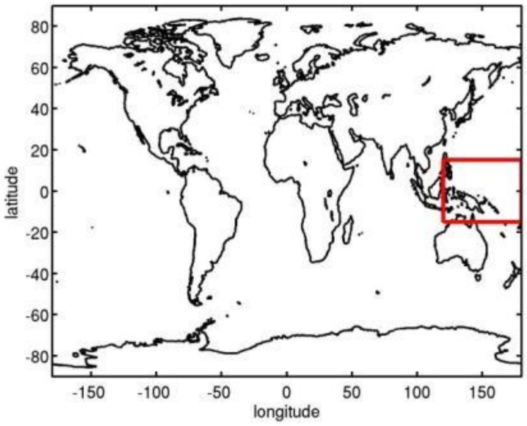

-180° ≤ λ ≤ -120°, 15° ≤ φ ≤ 45°, with topographic reduction

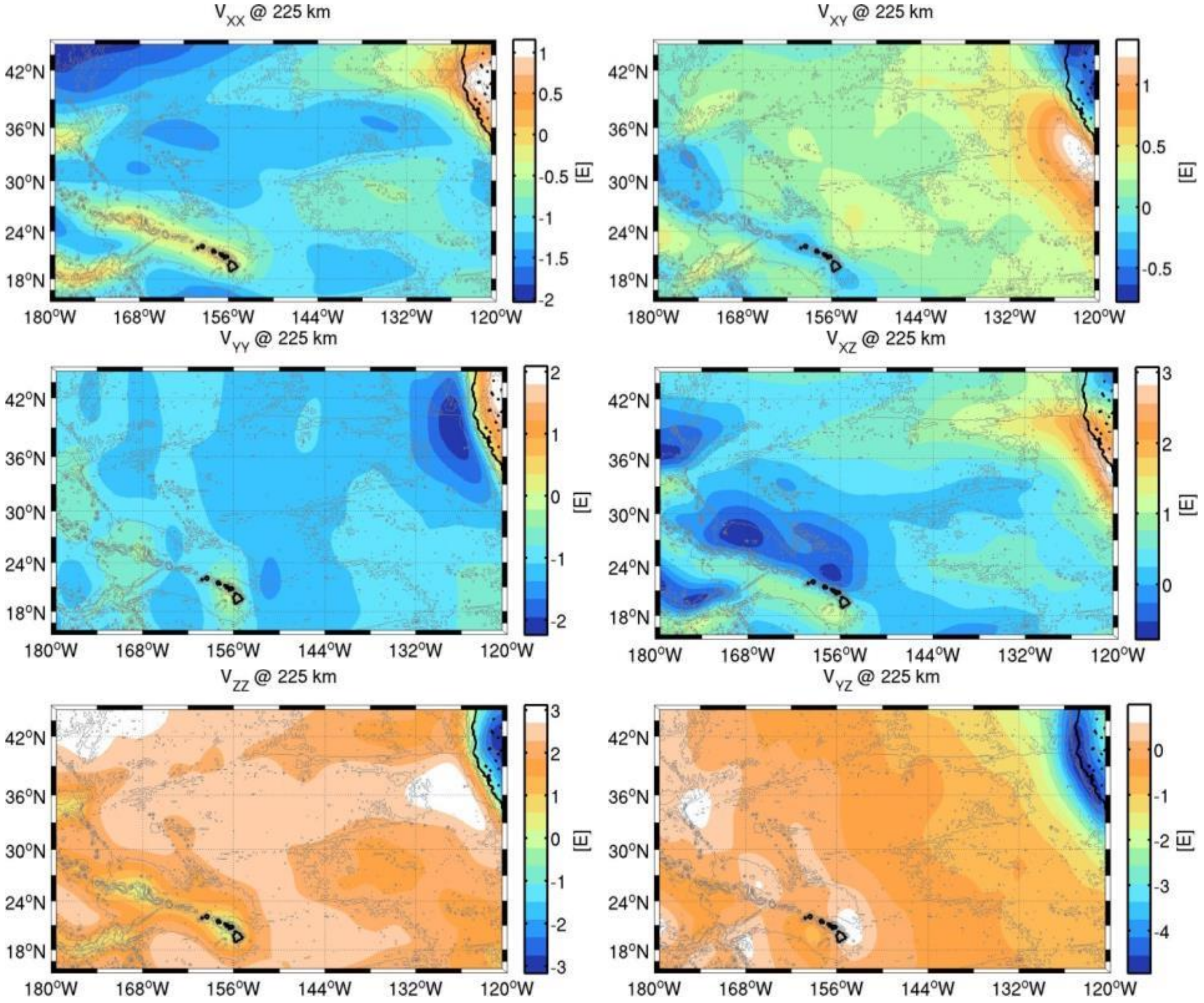

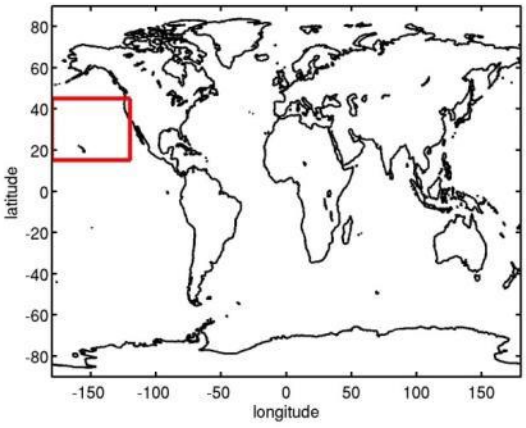

-120° ≤ λ ≤ -60°, 15° ≤ φ ≤ 45°, with topographic reduction

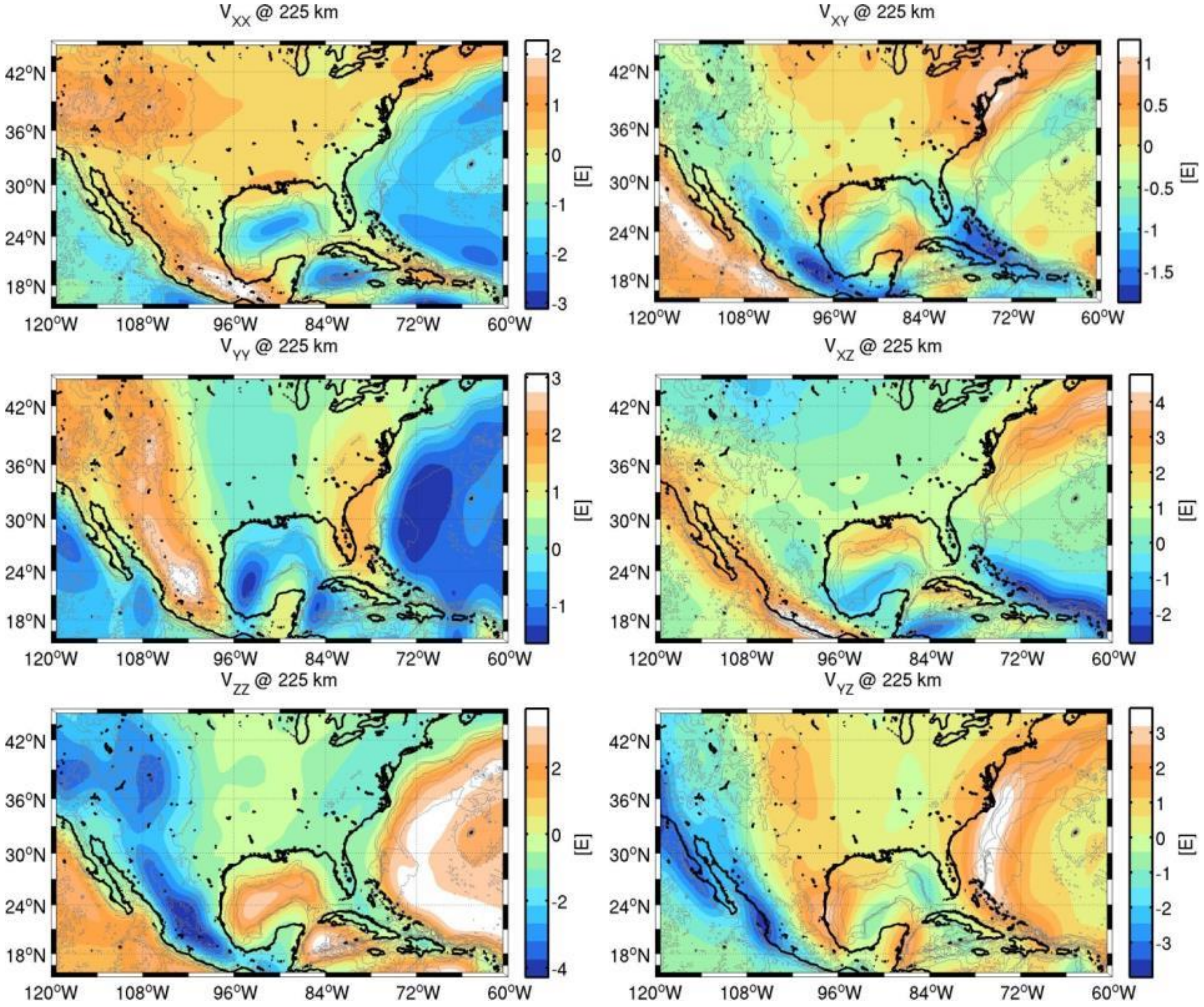

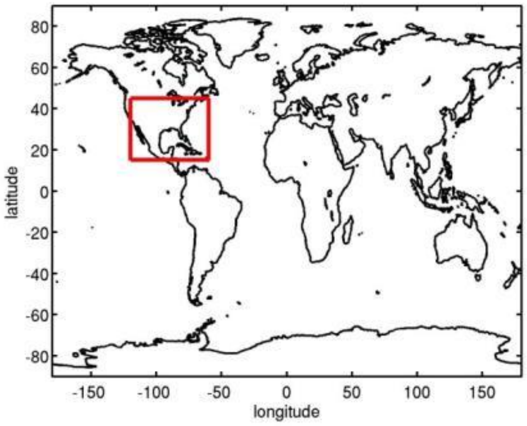

-180° ≤ λ ≤ -120°, 15° ≤ φ ≤ 45°, with topographic reduction

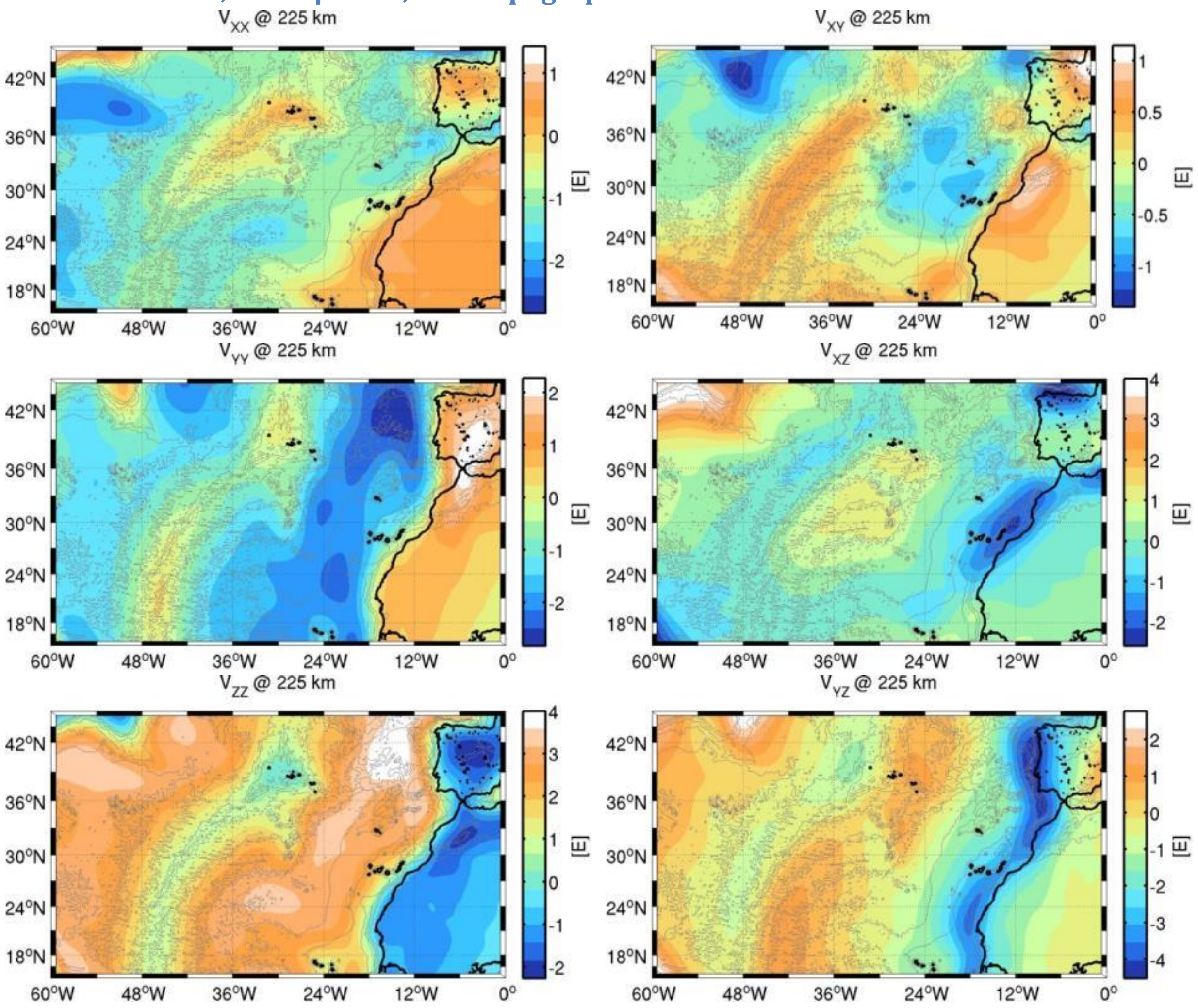

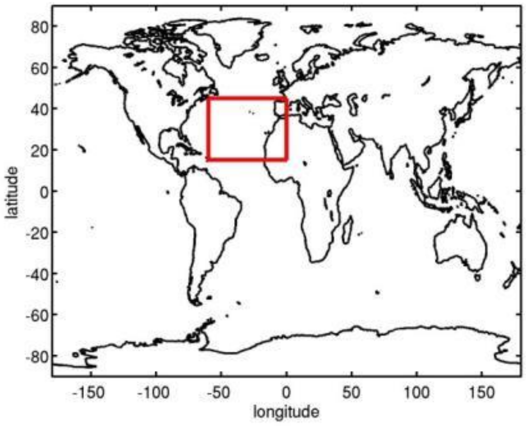

0° ≤ λ ≤ 60°, 15° ≤ φ ≤ 45°, with topographic reduction

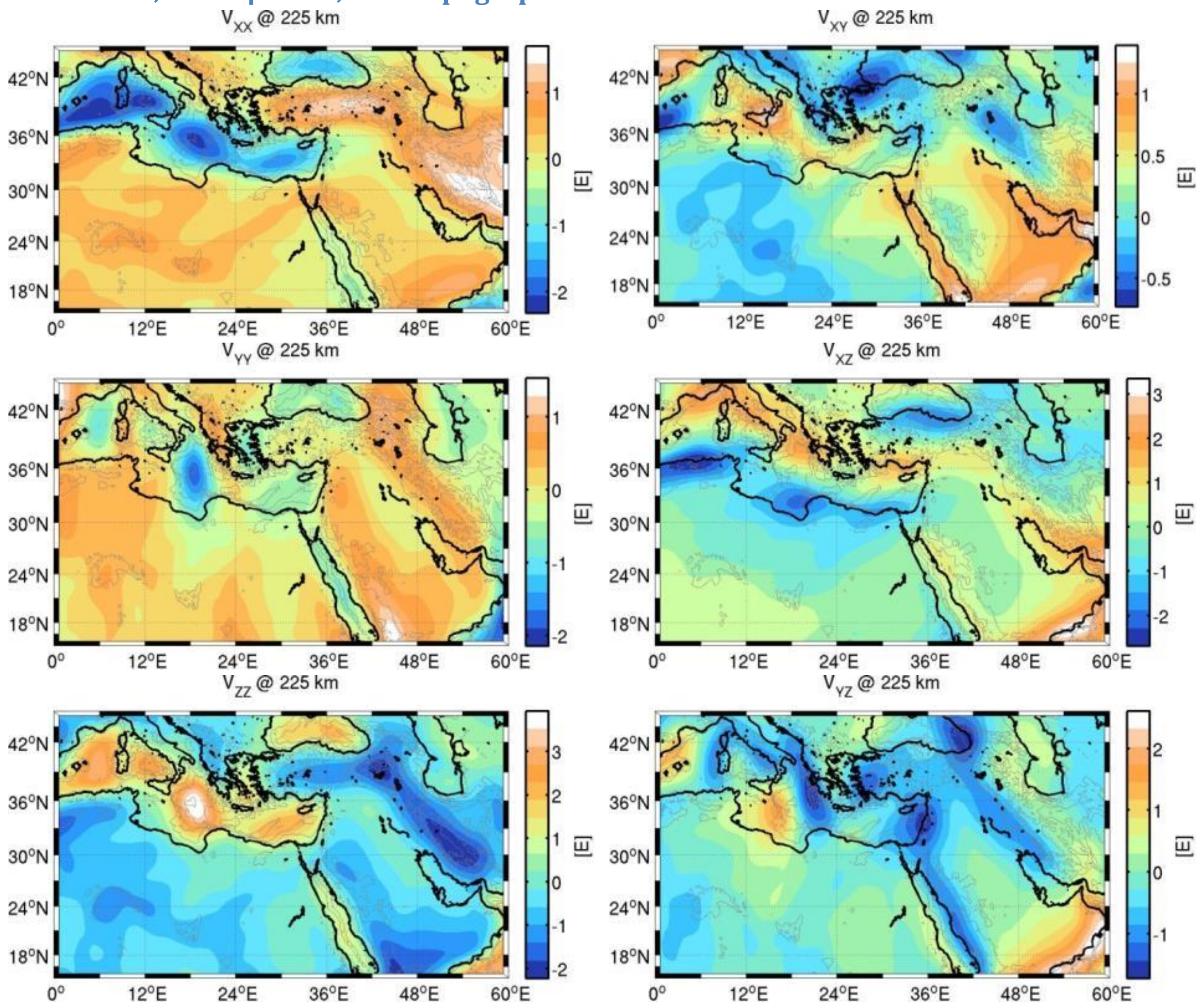

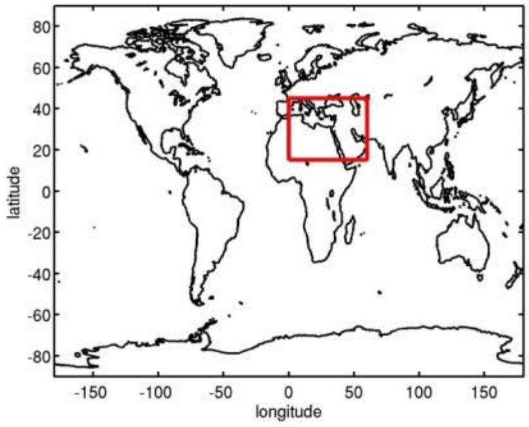

60° ≤ λ ≤ 120°, 15° ≤ φ ≤ 45°, with topographic reduction

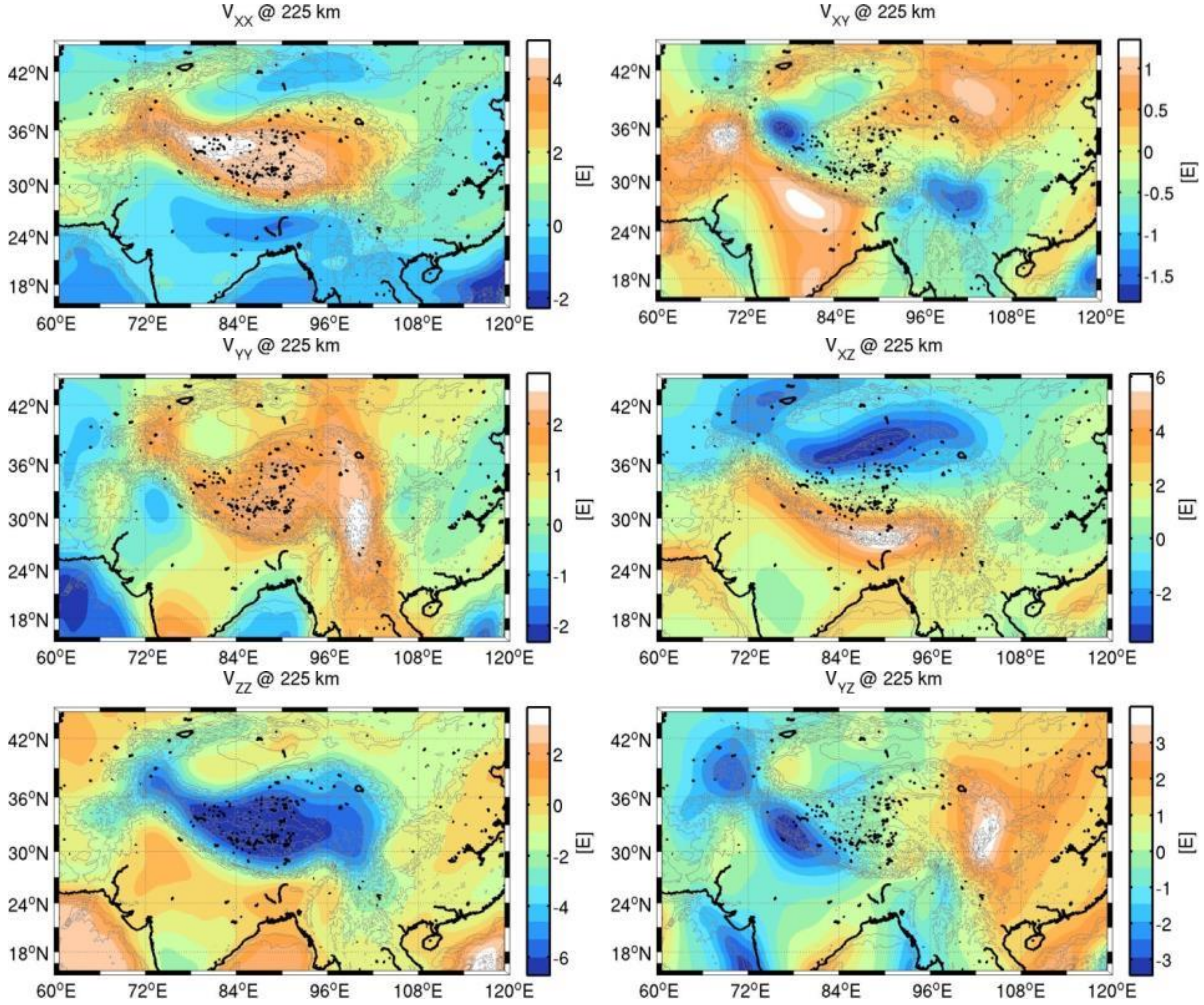

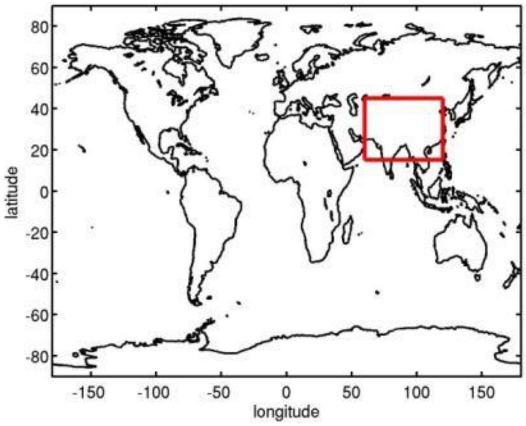

120° ≤ λ ≤ 180°, 15° ≤ φ ≤ 45°, with topographic reduction

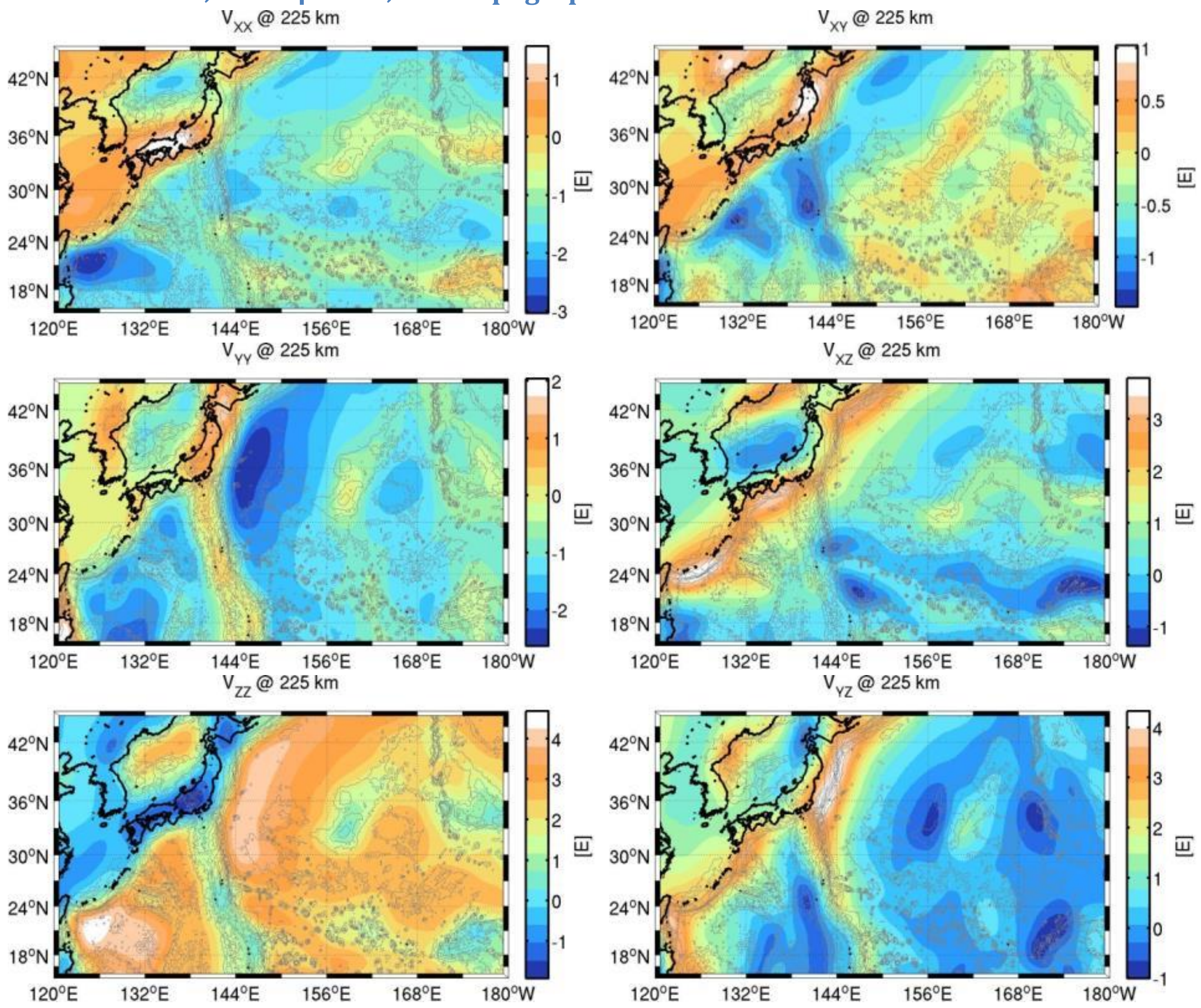

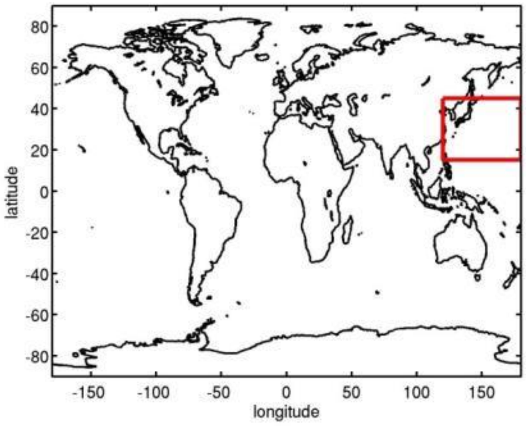

-180° ≤ λ ≤ -120°, 45° ≤ φ ≤ 75°, with topographic reduction

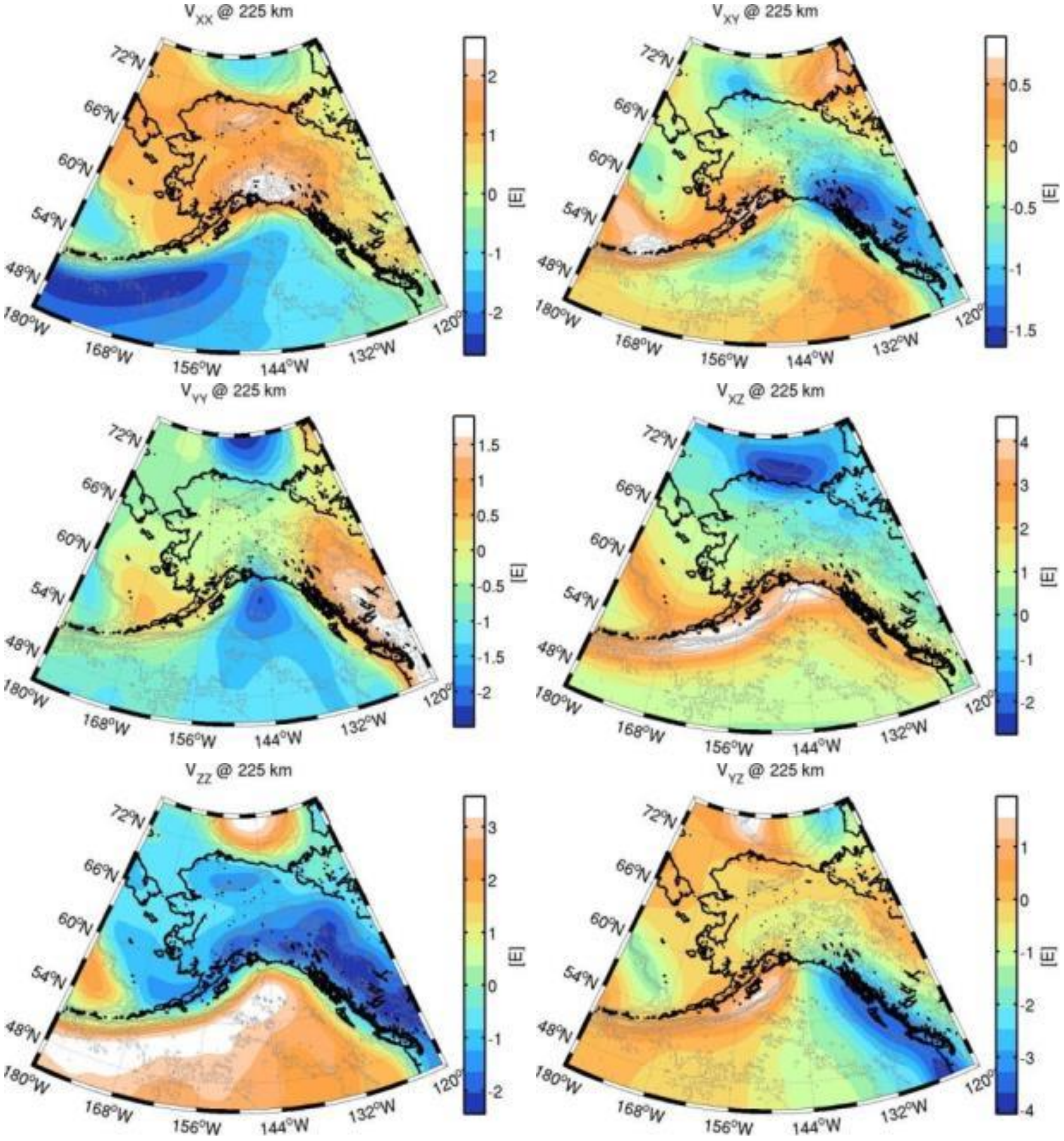

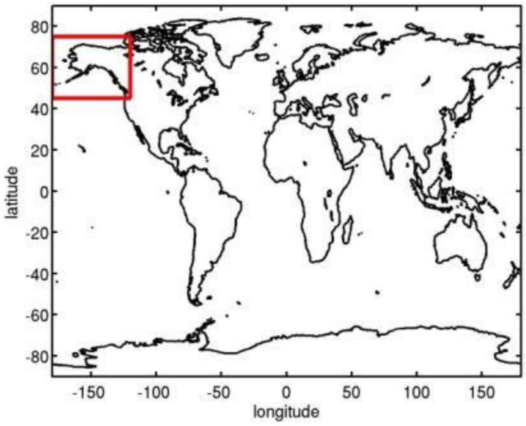

-120° ≤ λ ≤ -60°, 45° ≤ φ ≤ 75°, with topographic reduction

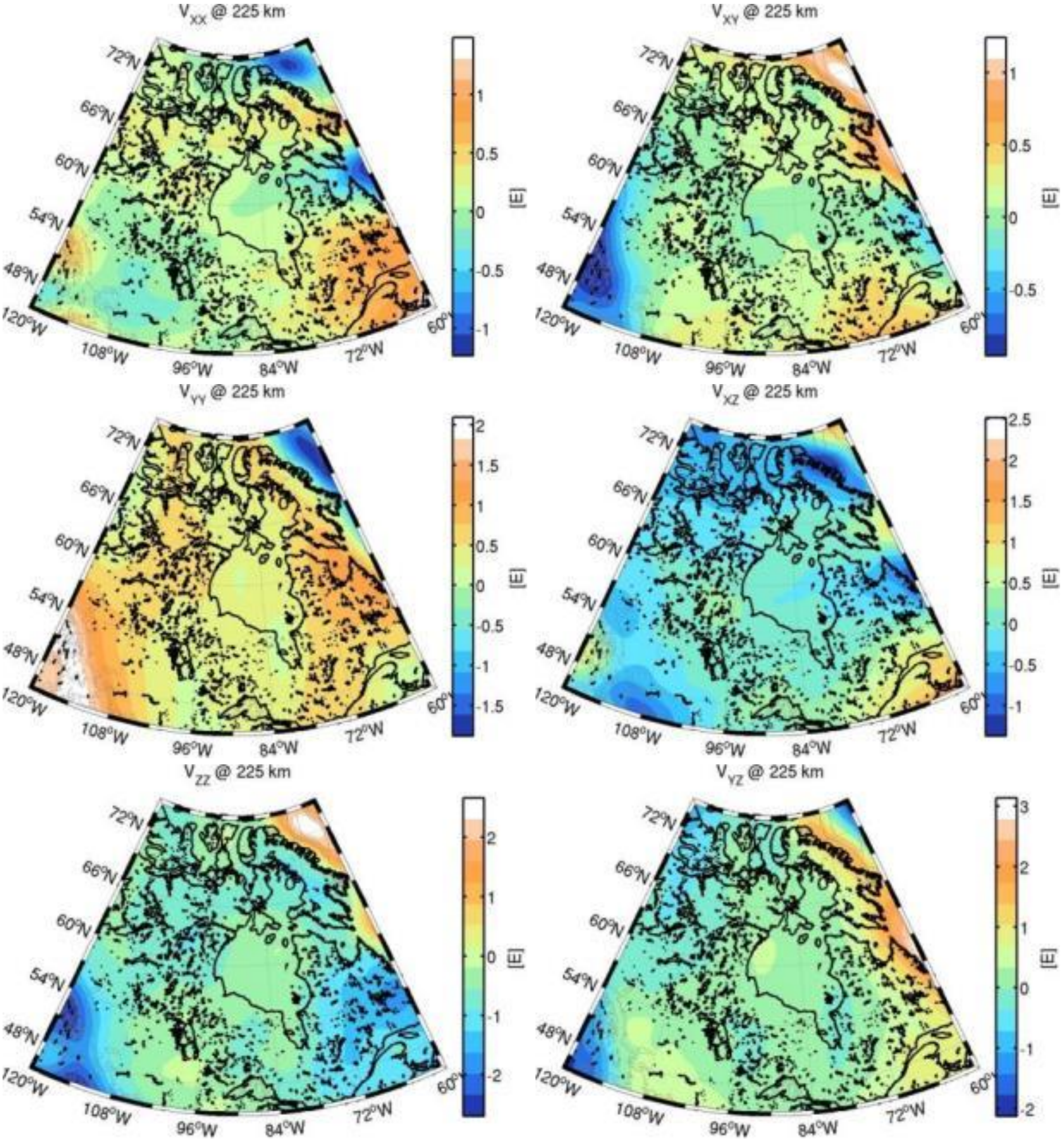

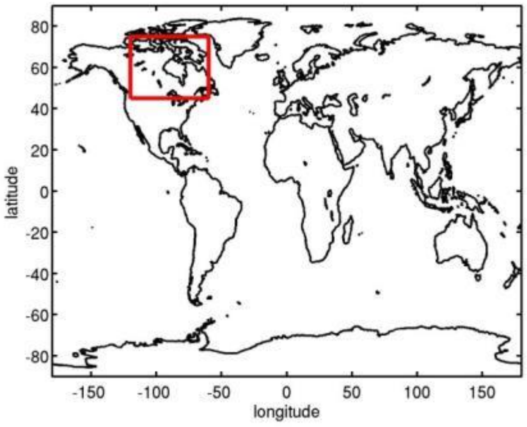

-60° ≤ λ ≤ 0°, 45° ≤ φ ≤ 75°, with topographic reduction

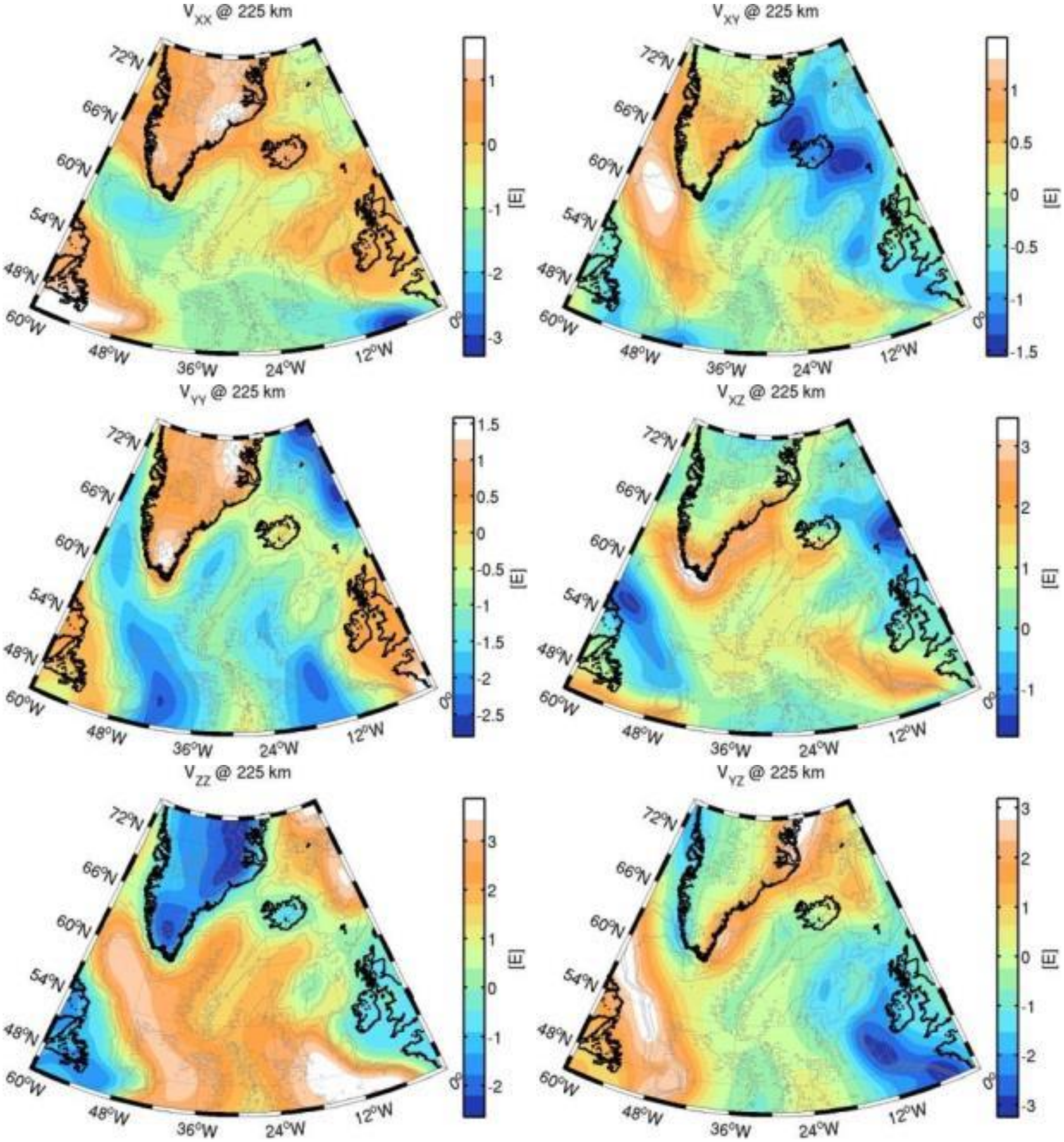

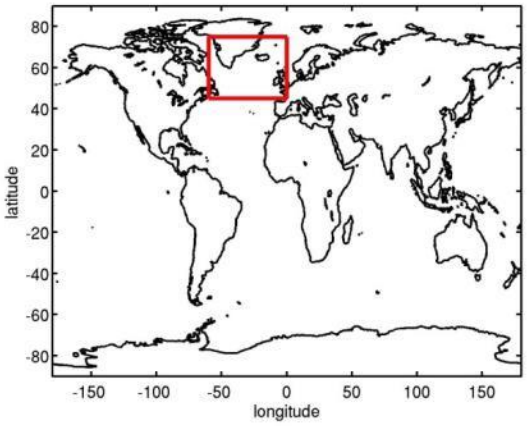

0° ≤ λ ≤ 60°, 45° ≤ φ ≤ 75°, with topographic reduction

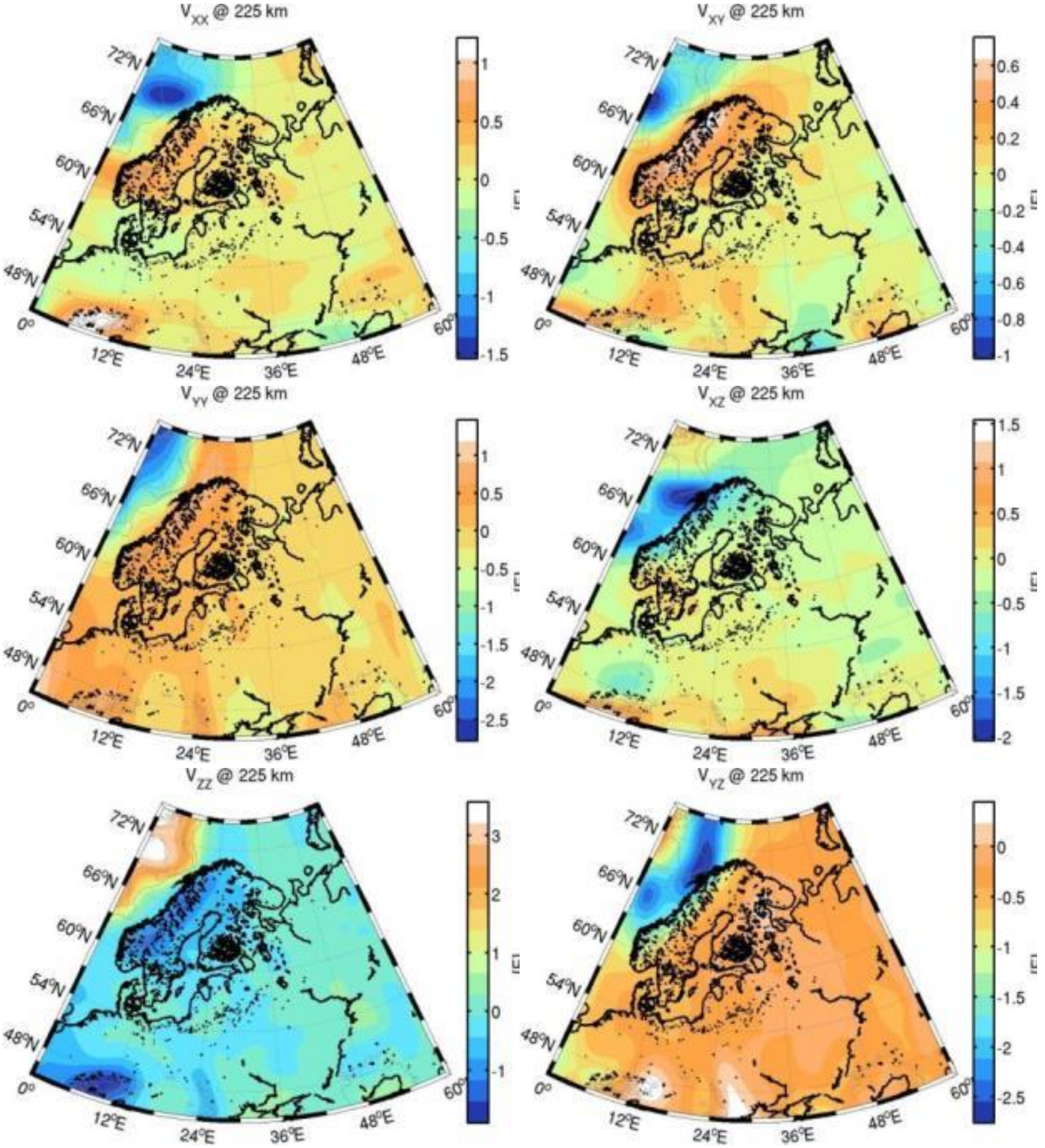

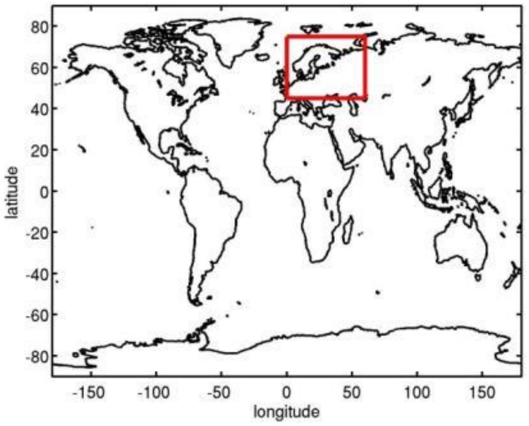

60° ≤ λ ≤ 120°, 45° ≤ φ ≤ 75°, with topographic reduction

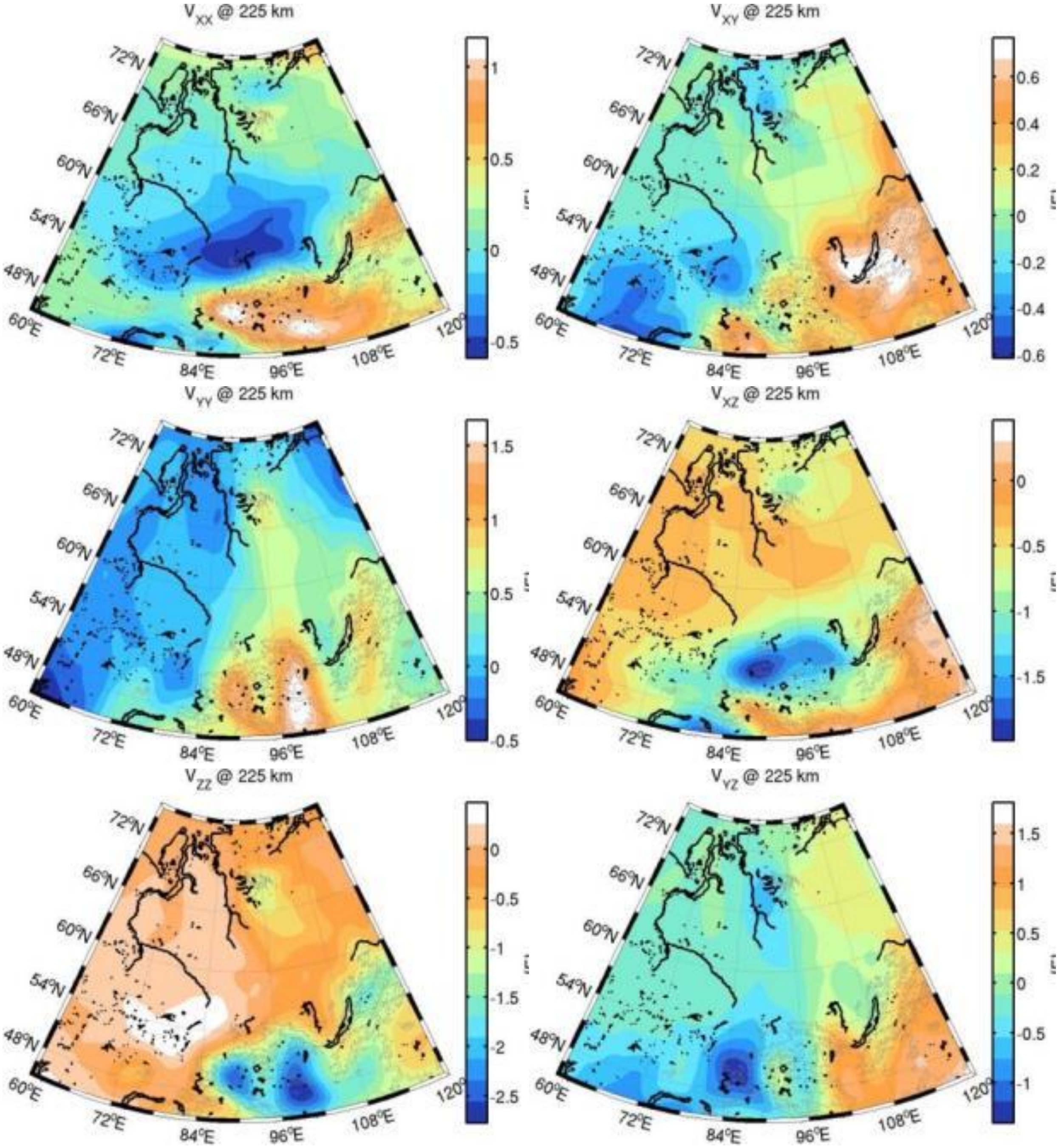

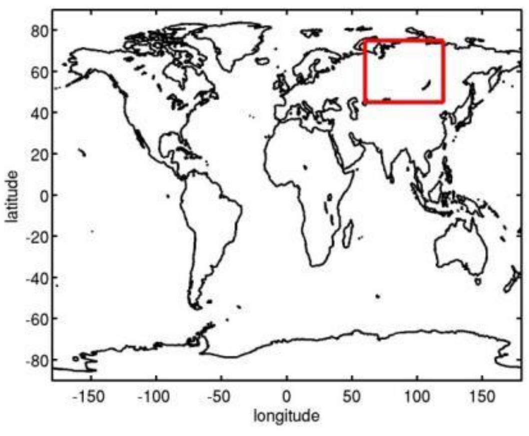

120° ≤ λ ≤ 180°, 45° ≤ φ ≤ 75°, with topographic reduction

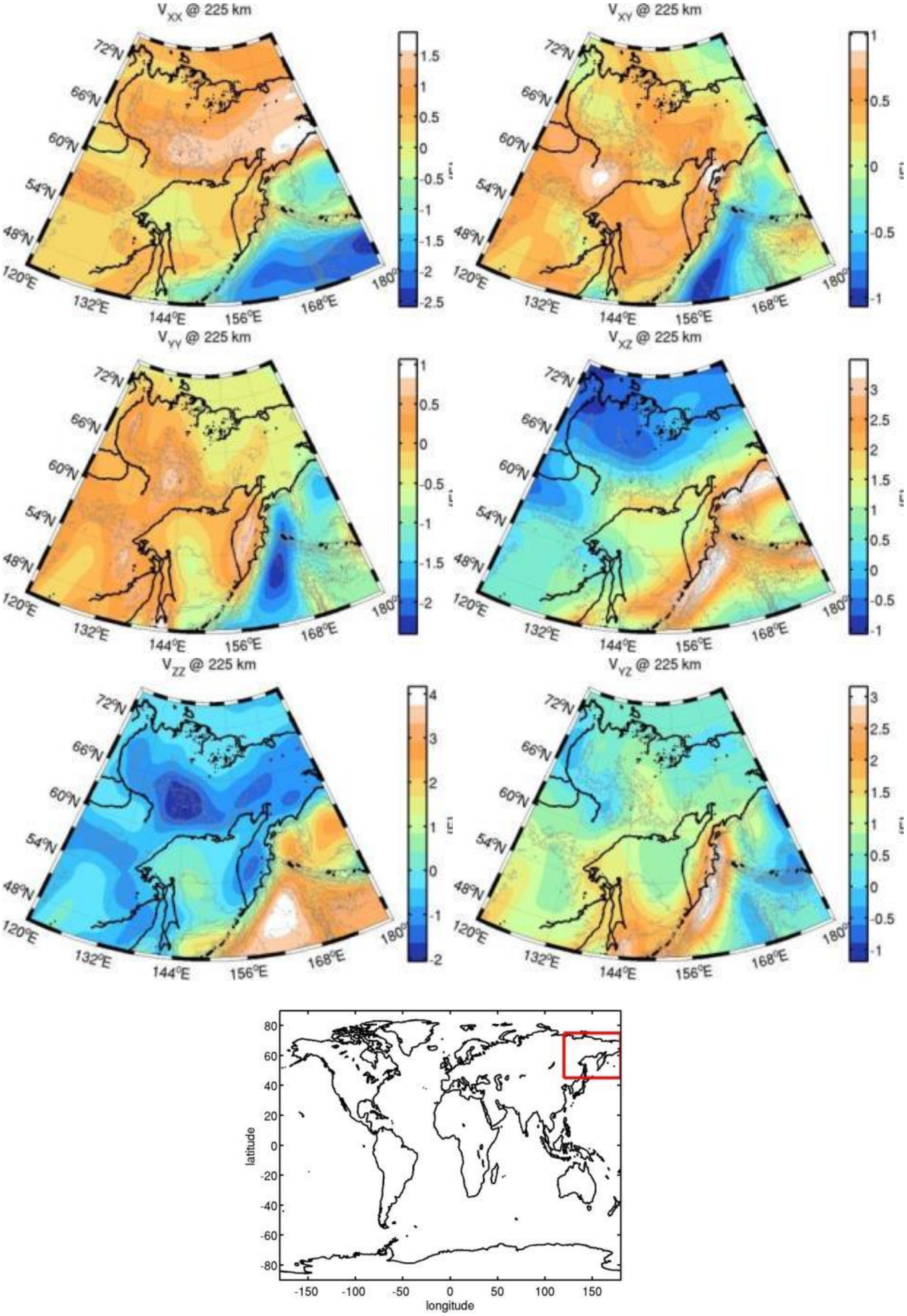

North Pole, with topographic reduction

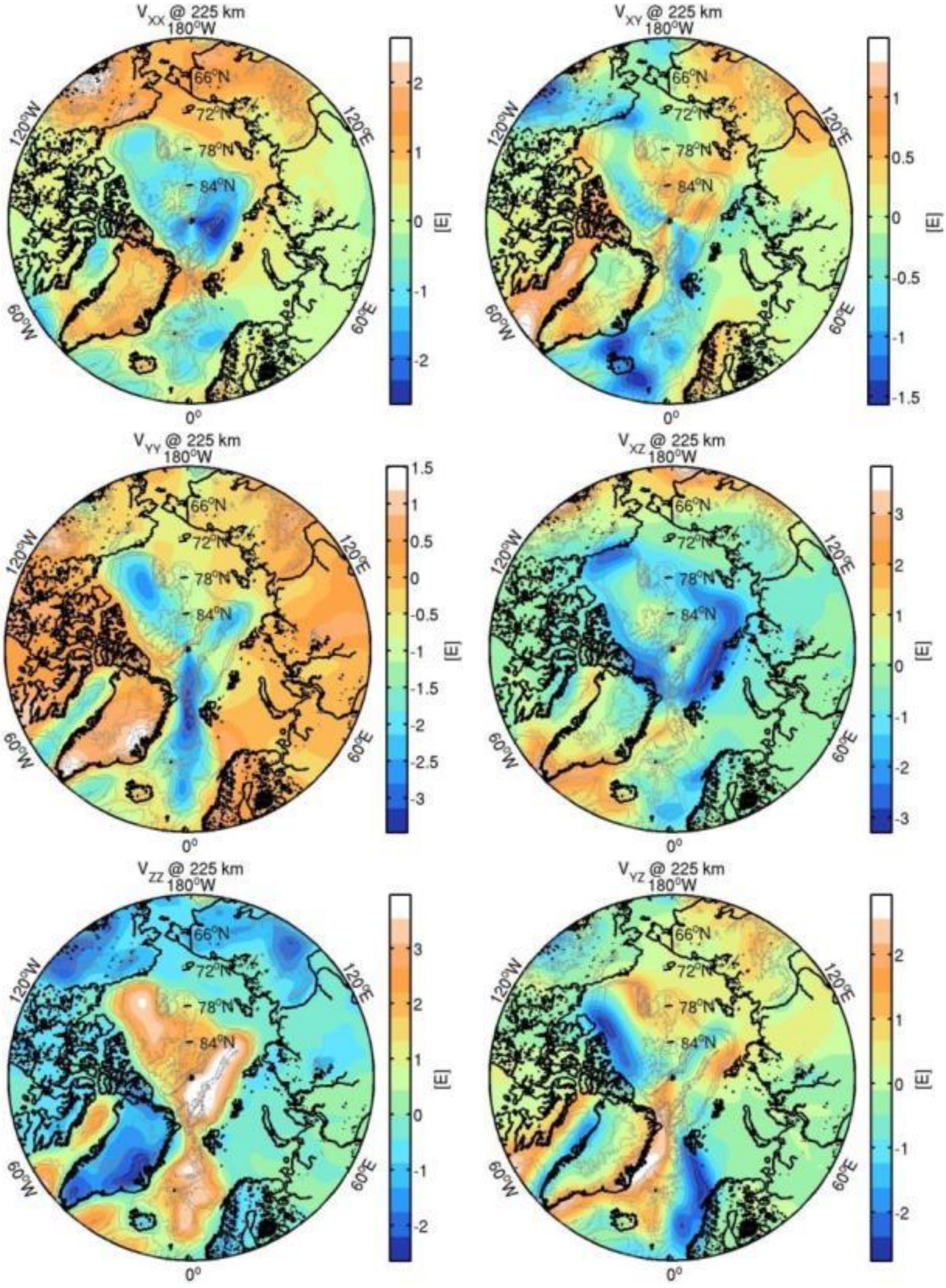

South Pole, with topographic reduction

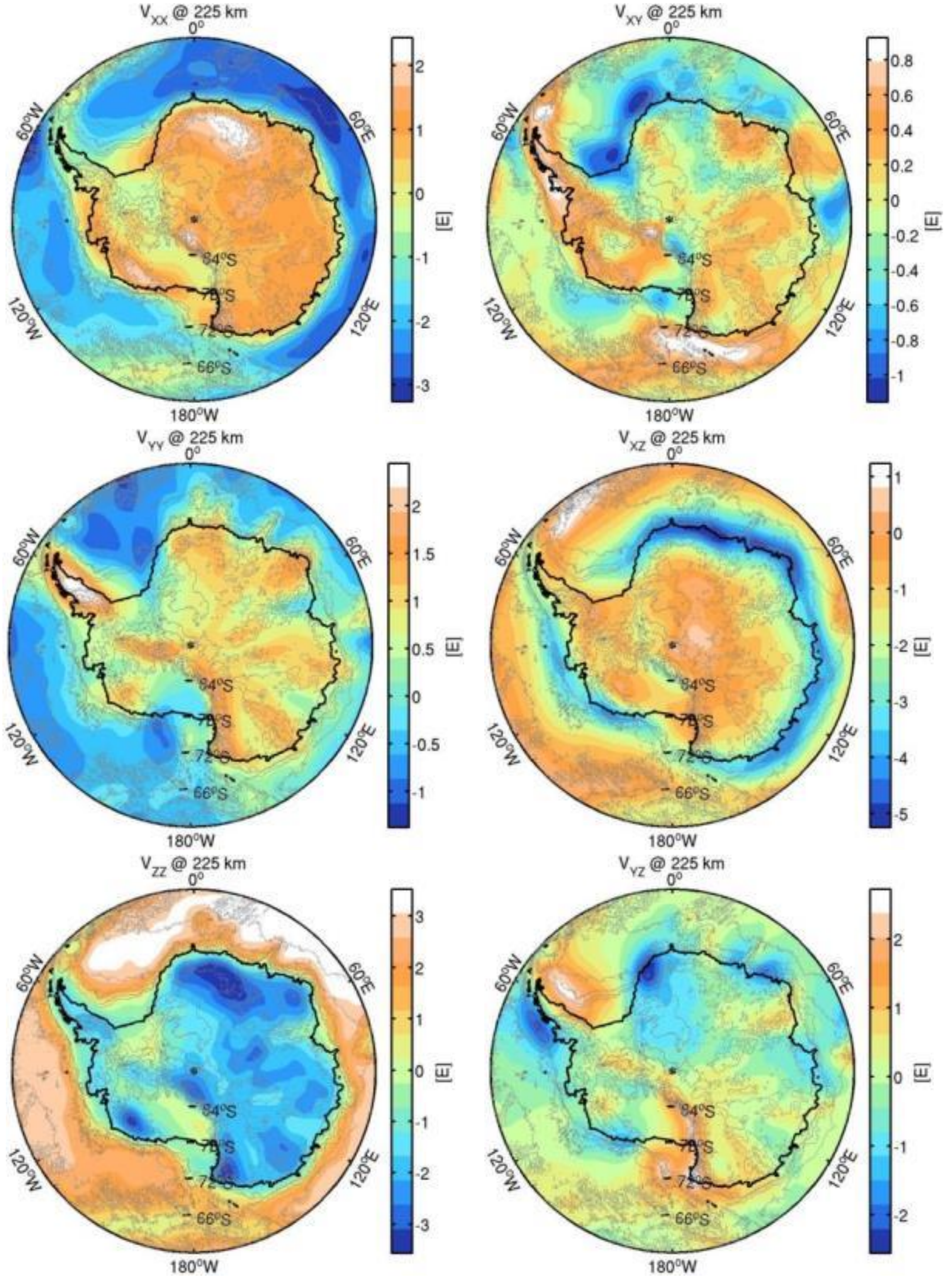

Supplement: Supplementary Information [file srep21050-s1.pdf]
